# Draft genome sequence of the rubber tree *Hevea brasiliensis*

**DOI:** 10.1186/1471-2164-14-75

**Published:** 2013-02-02

**Authors:** Ahmad Yamin Abdul Rahman, Abhilash O Usharraj, Biswapriya B Misra, Gincy P Thottathil, Kandakumar Jayasekaran, Yun Feng, Shaobin Hou, Su Yean Ong, Fui Ling Ng, Ling Sze Lee, Hock Siew Tan, Muhd Khairul Luqman Muhd Sakaff, Beng Soon Teh, Bee Feong Khoo, Siti Suriawati Badai, Nurohaida Ab Aziz, Anton Yuryev, Bjarne Knudsen, Alexandre Dionne-Laporte, Nokuthula P Mchunu, Qingyi Yu, Brennick J Langston, Tracey Allen K Freitas, Aaron G Young, Rui Chen, Lei Wang, Nazalan Najimudin, Jennifer A Saito, Maqsudul Alam

**Affiliations:** 1 Centre for Chemical Biology, Universiti Sains Malaysia, Penang, Malaysia; 2 TEDA School of Biological Sciences and Biotechnology, Nankai University, Tianjin, China; 3 Advanced Studies in Genomics, Proteomics and Bioinformatics, University of Hawaii, Honolulu, Hawaii, USA; 4 Ariadne Genomics Inc., Rockville, Maryland, USA; 5 CLC bio, Aarhus, Denmark; 6 Department of Biotechnology and Food Technology, Durban University of Technology, Durban, South Africa; 7 AgriLife Research Center, Department of Plant Pathology and Microbiology, Texas A&M University System, Weslaco, Texas, USA; 8 Department of Microbiology, University of Hawaii, Honolulu, Hawaii, USA; 9 Current address: Centre of Excellence in Neuromics of Université de Montréal, Centre Hospitalier de l'Université de Montréal Research Center, Montréal, Québec, Canada; 10 Current address: Bioscience Division, Los Alamos National Laboratory, Los Alamos, New Mexico, USA

**Keywords:** *Hevea brasiliensis*, Euphorbiaceae, Natural rubber, Genome

## Abstract

**Background:**

*Hevea brasiliensis,* a member of the Euphorbiaceae family, is the major commercial source of natural rubber (NR). NR is a latex polymer with high elasticity, flexibility, and resilience that has played a critical role in the world economy since 1876.

**Results:**

Here, we report the draft genome sequence of *H. brasiliensis*. The assembly spans ~1.1 Gb of the estimated 2.15 Gb haploid genome. Overall, ~78% of the genome was identified as repetitive DNA. Gene prediction shows 68,955 gene models, of which 12.7% are unique to *Hevea*. Most of the key genes associated with rubber biosynthesis, rubberwood formation, disease resistance, and allergenicity have been identified.

**Conclusions:**

The knowledge gained from this genome sequence will aid in the future development of high-yielding clones to keep up with the ever increasing need for natural rubber.

## Background

Rubber is an indispensable commodity used in the manufacture of over 50,000 products worldwide [1]. Approximately 2,500 plant species synthesize rubber [2], but *Hevea brasiliensis* (Willd.) Muell.-Arg., also known as Pará rubber tree, is the primary commercial source for natural rubber (NR) production. This member of the Euphorbiaceae family originated from the Amazon Basin, and it was not until the nineteenth century that it significantly began to be commercially exploited and its domestic cultivation was established outside of South America. Today, plantations are mainly found in the tropical regions of Asia, Africa, and Latin America. Rubber trees start yielding latex after reaching 5–7 years of maturity and have a productive lifespan of 25–30 years. According to the International Rubber Study Group (http://www.rubberstudy.com), global production of NR reached nearly 11 million tons in 2011 with Asia accounting for about 93% of the supply. The demand for rubber (natural and synthetic) has steadily risen over the past century and is expected to continue to increase.

NR is a latex polymer with high elasticity, flexibility, resilience, impact resistance, and efficient heat dispersion [2]. These properties make NR difficult to be replaced by synthetic rubber in many applications, such as medical gloves and heavy-duty tires for aircrafts and trucks. NR consists of 94% *cis*-1,4-polyisoprene and 6% proteins and fatty acids [3]. *Cis*-1,4-polyisoprene biopolymers are made up of C5 monomeric isopentenyl diphosphate (IPP) units and are formed by sequential condensation on the surface of rubber particles. The rubber chain elongation is catalyzed by *cis*-prenyltransferases (CPTs), known as rubber polymerases [4]. The molecular weight of the resulting polymer is an important determinant of rubber quality. Only a few plants produce large amounts of high quality NR (molecular weight > 1 million daltons), including *H. brasiliensis* and the potential alternative rubber crops *Parthenium argentatum* (guayule) and *Taraxacum koksaghyz* (Russian dandelion) [5].

In addition to NR, rubber trees are used as a source of timber, once their latex productivity is no longer economically viable. Rubberwood has become a major timber export of Southeast Asia [1]. Its natural light color and excellent physical properties make it suitable for flooring and household furniture. Owing to the value of this product, several superior latex-timber clones have been developed.

Some of the issues concerning the rubber industry include pathogen attack and allergenicity. Fungal diseases, such as South American Leaf Blight (SALB; caused by *Microcyclus ulei*) and leaf fall caused by *Colletotrichum, Oidium*, and *Corynespora*, are major threats to rubber production [1]. In the mid-1930s, SALB collapsed the rubber industry in Brazil. Asian plantations have not been hit by this disease yet, but an outbreak in the region could have devastating effects. The allergenicity of NR is an issue which continues to be a global medical concern for those repeatedly exposed to latex-containing products (e.g., gloves). These allergies are triggered by certain proteins present in *Hevea*-derived NR. In recent years, guayule has emerged as a source of hypoallergenic latex [2].

Difficulties with conventional breeding along with limited genome-based information have impeded efficient crop improvement of *H. brasiliensis*. Marker assisted selection can improve the efficiency of breeding by enabling the direct selection of targeted genotypes. Analysis of genetic linkage among markers and identification of the genetic locations of desirable phenotypes would further improve the selection accuracy. A recent surge in high-throughput sequencing efforts [6-12] has enhanced the genetic resources available for *H. brasiliensis*. However, whole-genome information is still lacking. While most of the studies have focused on transcriptome analysis, the non-coding regions of the genome are also essential for understanding the regulatory elements controlling gene expression, as well as for the development of a more comprehensive set of molecular markers. Here, we report the draft genome of *H. brasiliensis*, which provides a platform to help accelerate the future improvement of this economically important crop.

## Results and discussion

### Genome sequencing and annotation

We sequenced the genome of *H. brasiliensis* clone RRIM 600, a high yielding clone developed by the Rubber Research Institute of Malaysia (parentage: Tjir 1 × PB 86). The rubber tree genome is distributed over 18 pairs of chromosomes [13], with the haploid genome estimated to be ~2.15 Gb by Feulgen microdensitometry [14]. We used a whole-genome shotgun (WGS) approach to generate ~43× coverage of sequence data from the Roche/454, Illumina, and SOLiD platforms (in Additional file 1: Table S1). Newbler [15] was chosen as the assembler for the final assembly since the majority of the sequencing data came from the Roche/454 platform with relatively longer read length, especially for single end reads [16]. Repeat motif identification on preliminary assemblies, removal of repeat-matching raw sequencing reads, and stringent assembling parameters were applied. The final genome assembly, based on only 27.86 Gb data or ~13× coverage after filtering repeat-matching reads (in Additional file 1: Table S1), resulted in scaffolds totaling 1,119 Mb with an N50 of 2,972 bp (Table 1). We anchored 143 scaffolds and the associated 1,325 genes onto the 18 *H. brasiliensis* linkage groups based on 154 microsatellite markers [17] (in Additional file 2: Figure S1). Within the mapped scaffolds, 74 additional reported markers were also identified (in Additional file 1: Table S2). Most of the markers were located in the intergenic regions.

**Table 1:** **Assembly and annotation statistics for the *H. brasiliensis *genome**

	
Number of scaffolds	608,017
N50 length scaffolds (bp)	2,972
N50 count scaffolds	23,685
Largest scaffold (bp)	531,465
Smallest scaffold (bp)	484
Average scaffold length (bp)	1,840
Number of contigs	1,223,364
Minimum length of contig (bp)	200
GC content of contig (%)	34.17
Repeats length contig (%)	72.01
Number of predicted genes	68,955
Mean gene length (bp)	1,332
Mean predicted ORF length (bp)	696
Longest gene (bp)	15,597
Shortest gene (bp)	162
Highest number of exons/gene	35
Mean exon length (bp)	238
Mean intron length (bp)	332

We have exclusively used next-generation sequencing technologies for WGS assembly of the rubber tree genome, and only a few other plant genomes have taken a similar approach. The strawberry genome was sequenced using a similar combination of Roche/454, Illumina, and SOLiD reads (39× coverage) as in this study, but with a nearly 9× smaller genome (240 Mb) and considerably lower proportion of repetitive DNA (22%), much larger contigs/scaffolds could be achieved in that case [18]. On the other hand, the largest scaffolds we assembled (largest = 531.5 kb) were comparable with those of the cannabis genome (largest = 565.9 kb), which was assembled from Illumina and Roche/454 data [19]. The major challenge of assembling the rubber tree genome was due to its highly repetitive content. This was also a difficulty for the barley genome (5.1 Gb, 84% repetitive DNA), as the WGS assembly based on Illumina short reads resulted in relatively small contigs (N50 = 1,425 bp) [20]. However, when combined with a BAC-based physical map and high-resolution genetic map, a highly structured chromosome-level framework was produced. Efforts such as incorporating a physical map or other methods to provide long-range linking information will be the next step for improving the rubber tree draft genome assembly.

Using RepeatModeler and RepeatMasker, 72.01% of the assembly was identified as repetitive DNA (excluding low complexity regions and RNA genes). This is estimated to represent ~78% of the genome, similar to that of maize (85%) [21] and barley (84%) [20]. Long terminal repeat (LTR) retrotransposons are the dominant class of transposable elements (46.15% of total repeats), of which the Gypsy-type (38.20%) and Copia-type (7.38%) are the most abundant (in Additional file 1: Table S3). Less than 2% of the total repeat elements are DNA transposons. A major part of the repeat elements (50.24%) could not be associated with any known families.

Combining the evidences derived from several *ab initio* gene prediction programs along with transcriptome and protein alignments, EVidenceModeler (EVM) [22] predicted 68,955 gene models from the masked assembly (in Additional file 1: Table S4). The average gene, exon, and intron lengths are 1,332 bp, 238 bp, and 332 bp, respectively (Table 1). Of the 137,540 expressed sequence tags (ESTs) and assembled transcripts available for *H. brasiliensis*, 95.4% are represented in the genome (in Additional file 1: Table S5). To provide additional support for gene model prediction and validation, we generated leaf transcriptome sequences (1,085 Mb using Roche/454 and 4.89 Gb using Illumina), which were *de novo* assembled into 73,060 contigs (in Additional file 1: Table S6). Over 99% of these contigs and 81% of the Roche/454 transcriptome reads aligned to the genome assembly. These results indicate that the draft assembly represents a large proportion of the gene space.

Protein sequences from the final gene predictions were annotated through different databases, including the NCBI non-redundant database, SwissProt [23], InterPro [24], and KEGG [25] (in Additional file 1: Table S7). Eukaryotic orthologous groups (KOG) [26] analysis revealed a significantly higher number of proteins in the ‘signal transduction mechanisms’ (5,216), ‘posttranslational modifications, protein turnover, chaperones’ (2,886), and ‘carbohydrate transport and metabolism’ (1,665) categories (in Additional file 1: Table S8). In addition, leucine-rich repeats (LRR) are the most abundant Pfam [27] domain represented in the genome (in Additional file 1: Table S9). Among the gene models, 6.7% are predicted to have signal peptides, with the majority being plastidial and extracellular targeted (in Additional file 1: Table S10).

Other than protein-coding genes, we identified 729 tRNA genes including 12 suppressor (Sup) tRNAs, 32 pseudogenes, and 4 with undetermined function (in Additional file 1: Table S11). Clustering of tRNA genes was noticed and interestingly, all Sup tRNA genes were clustered into 2 scaffolds (9 in scaffold 134351 and 3 in scaffold 134362). We also identified 5S (113 copies), 5.8S (18 copies), 18S (11 copies), and 28S (21 copies) rRNA genes in the assembly.

### Phylogeny and lineage-specific genes

**Figure 1:**
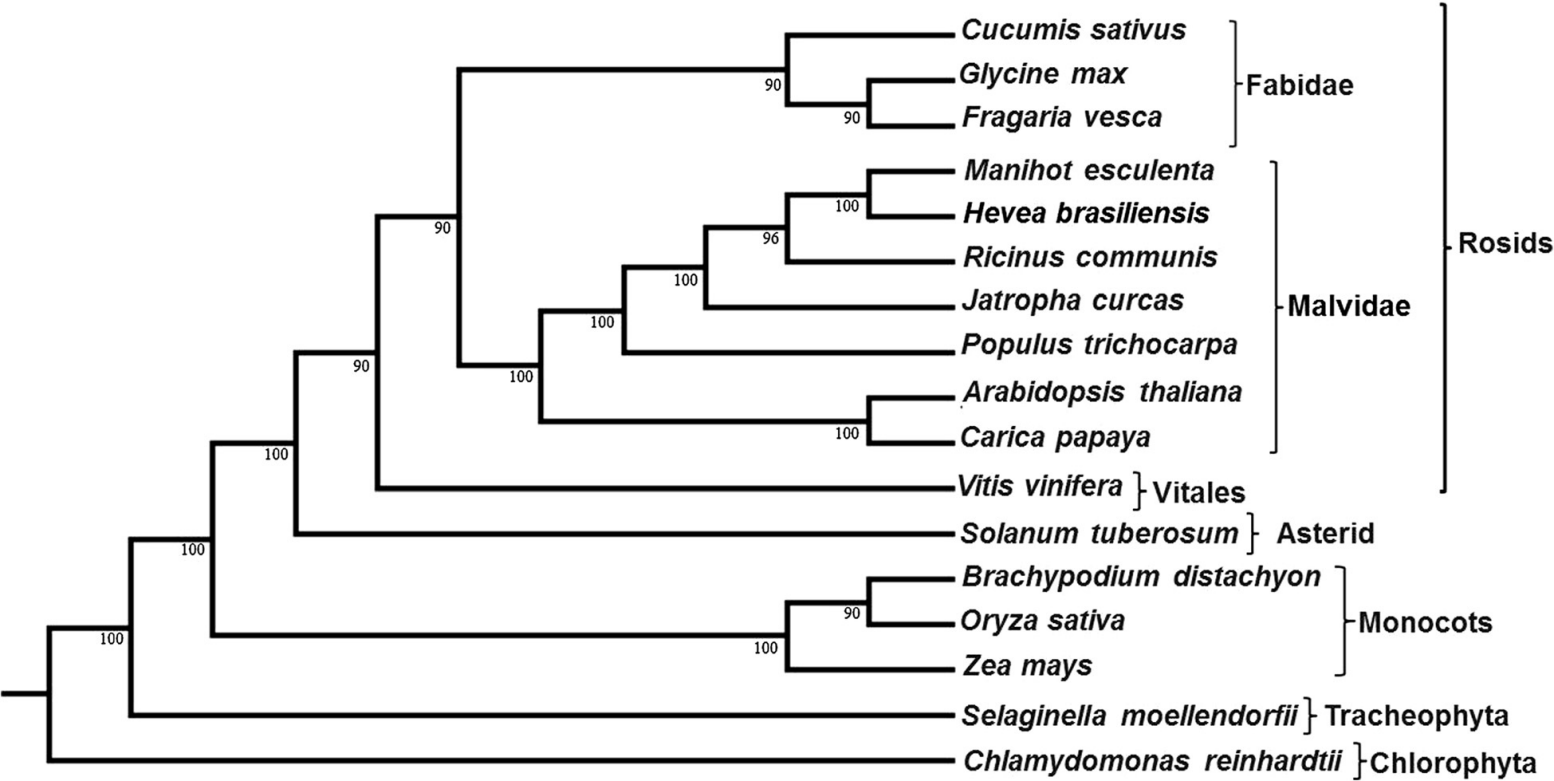
**Maximum likelihood phylogeny unveiling the taxonomic position of *H. brasiliensis*.** A phylogenetic tree was constructed using 144 orthologous single-copy gene clusters distributed across 17 species for which the genome sequences are available. The tree was constructed by the Maximum likelihood method using PhyML employing SPR and NNI for best tree improvement features. The analysis revealed the position of *H. brasiliensis* to be in Malvidae with more relatedness to *M. esculenta*. Bootstrapping procedures were applied over random 100 replicates and 7 seeds and the values are shown at nodes.

Phylogenetic analysis using 144 single copy orthologous clusters from 17 sequenced plant genomes shows that *H. brasiliensis* shares the closest ancestry with *Manihot esculenta* (Figure 1), consistent with the placement based on chloroplast genes [28]. Outside the Euphorbiaceae, the closest sequenced genome is of *Populus. trichocarpa*. In agreement with the angiosperm phylogeny derived from 154 nuclear genes [18], our analysis reveals that Malpighiales (includes Salicoid member *Populus* and the Euphorbiaceae members) shares a common ancestry with other members in Malvidae.

**Figure 2:**
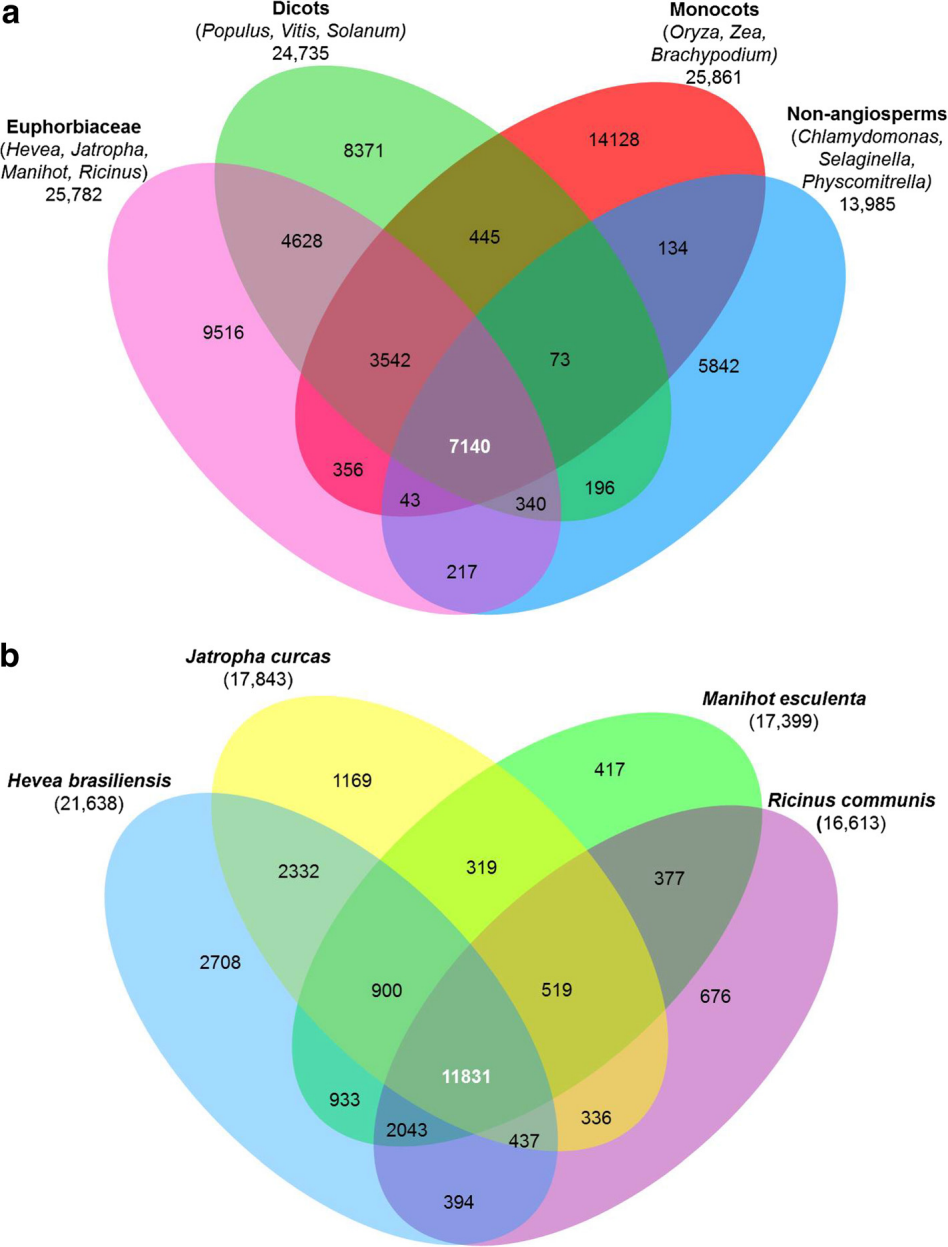
**Venn diagrams showing the distribution of unique and shared gene families.** OrthoMCL was used to identify gene clusters across 13 plant species (**a**) as well as between the four sequenced Euphorbiaceae members (**b**).

We compared 13 representative plant genomes (grouped into Euphorbiaceae, dicots, monocots, and lower plants) and found that a core gene set of 7,140 clusters are common to all groups, while 9,516 are unique to the Euphorbiaceae (Figure 2a). Comparison of the four sequenced Euphorbiaceae genomes (*Jatropha, Ricinus, Manihot*, and *Hevea*) indicated that 2,708 clusters comprising 8,748 genes are *Hevea* specific (Figure 2b). We were able to assign 526 Gene Ontology (GO) [29] categories (in Additional file 1: Table S12), 266 InterPro domains (in Additional file 1: Table S13), and 267 Pfam domains (in Additional file 1: Table S14) to these *Hevea* specific genes. The most abundant InterPro and Pfam domains belong to LRR and protein kinases. KOG analyses revealed that majority of the genes are associated with signal transduction, cytoskeleton, and posttranslational modification (in Additional file 1: Table S15).

### Rubber biosynthesis

**Figure 3:**
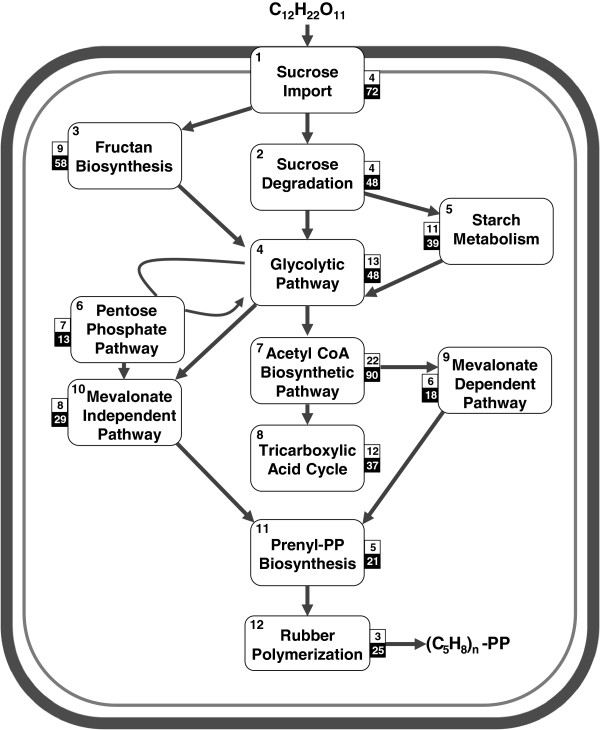
**Schematic representation of the metabolic pathway leading to natural rubber biosynthesis.** Import of sucrose until biosynthesis of rubber involves 12 sub-metabolic pathways represented in the large boxes. The number of enzymes and associated proteins in each individual pathway is shown in small white boxes and the number of orthologs in *Hevea* in the grey boxes. The detailed pathway is shown in Additional file 2: Figure S2.

Rubber biosynthesis involves fixation of carbon in the leaf, loading and transportation of the assimilates, specialized metabolic processes driving the precursors for biosynthesis, and storage of polyisoprenes in the laticifer. Sucrose provides the carbon skeleton and energy supply for rubber biosynthesis, with laticifers serving as its strong sink [30]. We reconstructed the entire metabolic pathway of rubber biosynthesis in *H. brasiliensis* (in Additional file 2: Figure S2). The carbon assimilatory mechanism of rubber biosynthesis consists of 12 distinct sub-metabolic pathways (Figure 3), represented by 417 genes (in Additional file 1: Table S16). We validated the expression of these genes from leaf and/or latex cDNA pools, detecting at least one isoform for the majority of the gene families (in Additional file 1: Table S16). We also made a comparison with the ESTs obtained from the rubber-producing bark tissue of guayule [31]. We found that 360 of the *H. brasiliensis* genes were represented in the guayule ESTs, of which 205 showed more than 70% sequence identity with the best match (in Additional file 2: Table S17).

It has been shown that sucrose transporters and their expression patterns are directly related to tapping and rubber production [32]. Sucrose and monosaccharides are imported into the laticifer cytosol via sucrose (SUT) and monosaccharide (MT) transporters which are encoded by 7 and 30 genes, respectively. β-fructofuranosidase and fructan β-fructosidase convert sucrose into monosaccharides, and the high number of genes (31) found in *H. brasiliensis* indicates the importance of this function for rubber biosynthesis. Excess sucrose is stored as fructan and starch which can later be used as a carbon source for rubber biosynthesis. Fructan metabolism consists of 9 enzymatic steps (encoded by 54 genes), while starch metabolism involves 11 reactions (encoded by 38 genes). Carbon is directed through glycolysis (encoded by 52 genes), alternative pentose phosphate pathway (encoded by 14 genes), and acetyl CoA biosynthetic pathway (encoded by 120 genes) to produce intermediate substrates for the biosynthesis of rubber precursors.

Isoprenoid precursors for rubber biosynthesis are provided by the cytosolic mevalonate dependent (MVA) pathway in the form of IPP [33]. The plastidic mevalonate independent (MEP) pathway is also suggested to contribute IPP for rubber biosynthesis [34]. Recently, ^13^C-labelled studies on *Hevea* seedlings suggested that the MEP pathway contributes IPP for carotenoid biosynthesis, but not for rubber biosynthesis [35]. However, expression analysis on MVA and MEP pathway genes suggest that the MEP pathway can be an alternate provider of IPP in mature rubber trees or in clones which do not produce a large amount of carotenoid [8]. In the *H. brasiliensis* genome, we identified 18 genes encoding enzymes for the MVA pathway and 29 for the MEP pathway. For the initiation of rubber biosynthesis, a priming allylic diphosphate (farnesyl diphosphate, geranylgeranyl diphosphate, or undecaprenyl diphosphate) is needed [36]. The biosynthetic pathway leading to these compounds involves 5 enzymatic steps encoded by 21 genes in the assembly.

**Figure 4:**
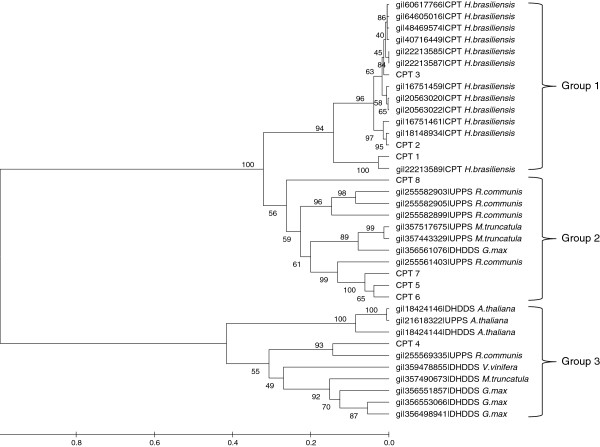
**Phylogenetic analysis of plant CPTs.** The evolutionary history was inferred by the Maximum likelihood method using MEGA5.05 [38]. All positions containing gaps and missing data were eliminated. CPT, *cis*-prenyltransferase; UPPS, undecaprenyl pyrophosphate synthase; and DHDDS, dehydrodolichyl diphosphate synthase. Bootstrapping values (100 replicates) are shown on branches.

Rubber polymerases, involved in the polymerization of isoprenoids, belong to the family of CPTs [37]. We identified eight CPTs from the genome (designated as CPT 1–8) which are divided into three groups according to evolutionary relationships (Figure 4). We found that *H. brasiliensis* CPTs in groups 2 and 3 are homologous to other plant CPTs (undecaprenyl pyrophosphate synthase and dehydrodolichyl diphosphate synthase) which are responsible for the elongation of short-chain C5-isoprenes (C_55_ – C_120_). Group 1, comprising CPT 1–3, is specific to *H. brasiliensis* and members belonging to this group were proven to catalyze the formation of long chain C5-isoprenes [4]. Only CPT 4 (group 3) has introns and it shares the least homology with others. Small rubber particle protein (SRPP) and rubber elongation factor (REF) are two other key proteins involved in rubber biosynthesis [5] and are represented by 10 and 12 genes, respectively, in our assembly.

### Rubberwood

Matured rubber trees that have reached the end of their latex-producing cycle are used as a source of timber for the manufacture of furniture and other products. Wood quality is associated with several lignocellulose biosynthesis genes [39], and we identified 127 genes in *H. brasiliensis* (in Additional file 1: Table S18). There are 36 cellulose synthase (CesA)-coding genes compared with 10 in *Arabidopsis*[40] and 18 in *Populus*[41]. Genes associated with hemicellulose biosynthesis have been identified in *H. brasiliensis* and is similar to *Populus* in having more α-L-fucosidases over α-L-fucosyltransferases [42]. Lignin, a heteropolymer of monolignols, determines the texture and hardness of the wood. The complete set of genes involved in monolignol biosynthesis (in Additional file 2: Figure S3) showed the highest similarity with *Populus* genes. In comparison with the *Populus* genome, caffeic acid O-methyl transferase (COMT) and cinnamyl alcohol dehydrogenase (CAD) showed noticeable difference in their numbers (COMT: 10 in *Hevea*, 41 in *Populus*; CAD: 5 in *Hevea*, 24 in *Populus*). This is probably related to the hardness of *Populus* wood [39] compared with rubberwood. The genes involved in transport, storage, and mobilization of monolignols and its final polymerization into lignin have also been identified.

### Disease resistance

Rubber trees are highly susceptible to fungal diseases, so the identification of disease resistance genes is one of the major focuses of rubber tree research. Hypersensitive response (HR) is the early defense response that causes necrosis and cell death to restrict the growth of the pathogen. Plant signaling molecules, salicylic and jasmonic acids, play a critical role in activating systemic acquired resistance (SAR) and induce certain pathogenesis-related (PR) proteins [43]. The nucleotide-binding site (NBS)-coding R gene family is the largest group of disease resistance genes in plants [44]. In *H. brasiliensis* we identified 618 members in this family, comparable to *Oryza sativa*, which are divided into 6 sub-classes: toll-interleukin-like receptor (TIR)-NBS, coiled-coil (CC)-NBS, NBS, TIR-NBS-LRR, CC-NBS-LRR, and NBS-LRR (in Additional file 1: Table S19). The majority were those without LRR domains, in contrast with other plants where the LRR-containing classes are typically more abundant. We also identified 147 PR and 96 early defense (SAR and HR) associated genes in the assembly (in Additional file 1: Tables S20 and S21). All these disease resistance genes were distributed in 665 scaffolds, and NBS-coding genes were often found to be in clusters (*e.g*., 9 NBS-LRR genes in scaffold 409956). In addition, we have reconstructed the SAR and HR signaling pathways for *H. brasiliensis* (in Additional file 2: Figures S4 and S5). The overall information can be potentially exploited for the biotic stress management of the plant.

### Latex allergens

Allergy to natural rubber latex (NRL) is one of the major global medical concerns. There are 14 internationally recognized NRL allergens, known as Hevb 1 to Hevb 14 (http://www.allergen.org). These are encoded by 100 genes in *H. brasiliensis* (in Additional file 1: Table S22). Most of the allergens are stress and defense-related proteins highly abundant in the latex [45]. Of the major allergens causing sensitization to NRL, Hevb 6 (hevein) is encoded by 16 genes whereas Hevb 5 is only a single copy gene. Hevb 1 (REF) and Hevb 3 (SRPP), associated with rubber particle, are represented by 12 and 10 genes, respectively. Hevb 4 (lecithinase homolog) with 5 genes and Hevb 13 (esterase) with 9 genes are also known as glycoallergens. There are 6, 4, and 2 genes coding for the cross-reactivity proteins Hevb 8 (profilin), Hevb 9 (enolase), and Hevb 10 (manganese superoxide dismutase), respectively. Defense-related allergens Hevb 2 (β-1,3-glucanase) and Hevb 11 (chitinase) are with 11 genes each. Domain analysis of Hevb 11 shows the presence of an 18–23 amino acid long signal peptide which confers the systemic wounding response in plants [46]. Other than the aforementioned latex allergens, 4 types of non-latex allergens (pollen allergen, α-expansin, β-expansin, and isoflavone reductase) were also identified in *H. brasiliensis* (in Additional file 1: Table S23).

### Transcription factors

The *H. brasiliensis* genome contains ~6000 transcription factors distributed in 50 major families (in Additional file 1: Table S24). Transcription factors account for 8.5% of gene models in *H. brasiliensis*. The bHLH, MYB, C3H, G2-like, and WRKY families are overrepresented. bHLH, the largest transcription factor family in most plants, is represented by 752 members. MYB, a diverse family of transcription factors that co-interacts with the bHLH family to regulate secondary metabolism [47] as well as biotic and abiotic stress, has 570 members. The C3H family, involved in floral development, embryogenesis, wintering and leaf senescence [48], is represented by 470 members followed by G2-like (461; photosynthetic regulation) [49] and WRKY (445; immune responses) [50]. MADS-box genes encoding homeotic floral transcription factors are divided into 5 groups, Mα, Mβ, Mγ, Mδ (or MIKC*), and MIKC^c^[51], and are represented by 112 members. There are 79 Type II MADS-box (Mδ and MIKC^c^ groups) genes in *H. brasiliensis* while the number is 54–67 in *Arabidopsis*, *Populus*, and *Oryza*. In contrast, only 33 Type I MADS-box (Mα, Mβ, and Mγ groups) genes are in *H. brasiliensis* compared to 29–94 present in the other 3 species. Only 12.5% (14 out of 112) MADS-box genes in *H. brasiliensis* were clustered compared to 47% in the *C. papaya* genome [52].

### Phytohormone biosynthesis and signaling

Phytohormone biosynthetic and signaling-related genes are well represented in *H. brasiliensis* (in Additional file 1: Table S25; in Additional file 2: Figures S6, S7, S8, S9, S10, S11, S12, S13, S14, S15, S16, S17). Angiosperms dedicate a larger proportion of their genomes to auxin signaling, as evident by 12 gene families. However, in *H. brasiliensis* a significant reduction in the number is observed for some of the auxin gene family members compared to other plants, especially for SAUR and IAA repressors. GA-20-oxidase, a key regulatory enzyme in gibberellin biosynthesis, has 5 orthologs in *H. brasiliensis* compared to one in *Ricinu*s and *Oryza.* The ethylene-responsive element binding factor (ERF) proteins are overrepresented (246 orthologs) in *H. brasiliensis*, compared to other plants. The increased number of ERF transcription factors may be involved in the ethylene-dependent processes specific to *H. brasiliensis*. Oxophytodienoic acid reductase, important in the jasmonic acid biosynthesis pathway, is encoded by 13 genes. Nitric oxide synthase, involved in the biosynthesis of nitric oxide as a defense mechanism, is a highly conserved single copy gene in *Arabidopsis*, *Ricinus*, *Populus*, and *Oryza*, whereas in *H. brasiliensis* there are 4 copies.

### Light signaling and circadian clock related genes

Light signaling pathways and circadian clocks are interconnected and have profound effects on the plant’s physiology. Light is one of the most important environmental signals processed by the circadian clock to synchronize appropriate timing of physiological events [53]. Expansion in the number of genes involved in photoperception and circadian rhythm is observed in *H. brasiliensis* (154), compared to *Populus* (77) and *Arabidopsis* (66) (in Additional file 1: Table S26). These results indicate the intense involvement of environmental signals in regulating the physiology of the rubber tree.

### F-box proteins

F-box proteins are part of the Skp1p-cullin-F-box protein complex involved in the ubiquitin/26S proteasome pathway responsible for the selective degradation of proteins [54]. They are characterized by a conserved F-box domain (40–50 amino acids) at the N-terminus [54] and are reported to be involved in the regulation of various developmental processes in plants such as leaf senescence, flowering, branching, phytochrome and phytohormone signaling, circadian rhythms, and self-incompatibility [55]. In *H. brasiliensis* there are 655 F-box genes, compared to 315 in *V. vinifera*, 198 in *C. papaya*, 425 in *P. trichocarpa,* 897 in *A. thaliana*, and 971 in *O. sativa*[56]. This is interesting and contrary to the belief that the F-box gene family is expanded in herbaceous annuals compared to woody perennials [55].

### Carotenoids

Carotenoids have a pivotal role in light harvesting, photoprotection, photomorphogenesis, lipid peroxidation, and a vast array of plant developmental processes [57]. Carotenoids are found in nearly all types of plastids including the Frey-Wyssling particles of rubber latex, imparting yellowish color to the latex of some clones. Although the role of carotenoids in the latex is not well-defined, it could be a competing sink for IPP in the laticifers. IPP from the MEP pathway is proposed to be utilized for *cis*-polyisoprene synthesis in clones having low carotenoid content in the latex [8]. It is observed that the genes for the carotenoid biosynthetic pathway in *H. brasiliensis* (48) underwent an expansion compared to the *A. thaliana* genome (28) (in Additional file 1: Table S27; in Additional file 2: Figure S18). Phytoene synthase and phytoene desaturase, the enzymes catalyzing the initial committed steps of carotenoid biosynthesis, are highly expanded in *Hevea* with 5 and 9 genes, compared to single copies in *Arabidopsis.* The overall observations indicate more efficient carotenoid biosynthetic machinery in *Hevea*, possibly with diverse functions.

## Conclusions

Given the pivotal roles of NR production and sustainability, this draft genome sequence is an invaluable resource added to the spurge family. It will facilitate and accelerate the genetic improvement of *H. brasiliensis* through molecular breeding and exploitation of genetic resources. We observed the occurrence of a higher percentage (~78%) of repeat elements which could be attributed to the increased rate of non-homologous recombination and exon shuffling [58], thereby reducing the consistency in the genetic purity of the progeny. The high percentage of repeat elements together with a lack of chromosome level information is the major hurdle in assembling the whole *H. brasiliensis* genome. The genome information together with the characterization of all available molecular markers linked to the desired genes will facilitate NR production by means of trait dependent molecular breeding. Alongside tree genome sequences available from *Populus*, *Eucalyptus*, and the herbaceous model *Arabidopsis*, rubber research would specifically get assistance in the key areas of latex production, wood development, disease resistance, and allergenicity.

## Methods

### Genome sequencing and assembly

High quality chromosomal DNA was extracted from young leaves of *H. brasiliensis* RRIM 600. Shotgun and paired-end (PE) libraries were prepared following the manufacturer’s instructions. High quality reads were generated by Illumina (200 bp and 500 bp PE), Roche/454 (shotgun, 8 kb PE, 20 kb PE), and SOLiD (2 kb PE) sequencers.

Preliminary genome assemblies were generated by two assemblers designed for *de novo* assembly of next-generation sequencing data [59], the CLC Workbench assembler (CLC bio, Denmark) and the Newbler assembler (version 2.3), with different input data content and assembling strategies (in Additional file 1: Table S1). Basic contigs of the CLC assembly were made from the de Bruijn graph of the quality trimmed Illumina 200 bp reads. All quality trimmed Illumina reads, Roche/454 reads, and SOLiD reads were used to connect basic contigs of the CLC assembly. Assembling parameter for the Newbler assembly was set as: large or complex genome, reads limited to one contig, minimum overlap length 50 bp, minimum overlap identity 95%. Contigs with length of at least 200 bp in each preliminary assembly were retained for further analyses.

RepeatModeler (version 1.0.4) [60] was applied on two preliminary assemblies with default parameters and extracted 2,323 repeat modules from the CLC assembly and 1,520 repeat modules from the Newbler assembly. Repeat libraries from two preliminary assemblies were screened for possible gene family related sequences through BLASTX searches on unclassified repeats against NR, KEGG, and TrEMBL [61] protein databases with E-value cutoff of 10^-5^, and were combined into a *H. brasiliensis* specific repeat library that contains 3,771 repeats. Occurrence frequency of repeats were used as criterion to screen the *H. brasiliensis* repeat library, and repeats appearing more than 100 times in each preliminary assembly were retained and combined into a *H. brasiliensis* high frequency repeat library as the input for RepeatMasker (version 3.2.9) [62]. This was used to identify and mask repeat regions in the Newbler generated preliminary assembly, with low complexity regions and RNAs not masked. The repeat masked preliminary Newbler assembly served as template to screen repeat associated sequencing reads produced by the Illumina platform. Before the screening process, the Illumina reads had undergone quality control and reads with all positions of quality value at least 25, with read length of 100 bp for the 200 bp library, read length of 85 bp for the first direction and 75 bp for the second direction for the 500 bp library, were retained. The beginning 50 bp of each quality screened Illumina read were used to align to the Newbler assembly by BOWTIE (version 0.12.7) with allowed mismatch positions of no more than 3. Read pairs with both reads mapped to repeat regions, or unpaired reads mapped to repeat regions, were excluded from the read data set. Quality control on sequencing reads produced by the SOLiD platform started with mapping the SOLiD reads to an earlier version of the CLC assembly generated from SOAP [63]-corrected Illumina 200 bp reads, allowing 2 errors of any kind (color space, single nucleotide difference, or indel). All the SOLiD read pairs where both reads matched, the corresponding reference sequences was cut out and used as a read pair. Paired SOLiD reads with length of at least 50 bp were retained in the read data set.

Final genome assembly was generated by the Newbler assembler on all Roche/454 reads, selected Illumina reads (paired and unpaired reads for the 200 bp library, paired reads for the 500 bp library), and SOLiD reads (in Additional file 1: Table S1). Repeat libraries from other plant species were obtained from the TIGR plant repeat databases and the TIGR maize repeat database, and ribosomal DNA sequences were removed from these databases. The *H. brasiliensis* specific repeat library and TIGR plant repeat libraries were combined as the input repeat library in RepeatMasker to identify and mask repeat regions in contigs of the final genome assembly, with low complexity regions and RNAs not masked. The first 50 bp of each of the genome sequencing reads were aligned to the final genome assembly by BOWTIE (version 0.12.7) [64] and transcriptome sequencing reads produced by the Roche/454 platform were aligned to the final genome assembly by TopHat (version 1.1.4) [65] for assessment of assembly completeness and coverage of coding regions.

To identify contigs with organellar origin, the assembly was searched by BLASTN against the *H. brasiliensis* chloroplast genome sequence and by BLASTX against proteins from organelle genomes of the Fabales as well as chloroplast genomes of *H. brasiliensis*, *J. curcas*, and *M. esculenta*, and mitochondrial genomes of *R. communis*, *Citrullus lanatus*, and *Cucurbita pepo*. Contigs originating from bacterial contamination were identified by screening against GenBank and removed from the final assembly.

### Transcriptome sequencing

Total RNA was isolated from *H. brasiliensis* leaves and libraries were prepared and sequenced according to the manufacturer’s protocols (Illumina and Roche/454). The initial transcriptome assembly was generated by assembling the Illumina reads using the CLC Workbench assembler. Contigs from the Illumina transcriptome assembly were cut into short fragments of at most 1999 bp and were combined with the Roche/454 transcriptome sequencing reads as the input of the Newbler assembler optimized for EST data. Contigs of the transcriptome assembly were annotated by BLASTX searches with E-value cutoff of 10^-5^ against the NR protein database to test transcript completeness and diversity.

### Genome annotation

Gene space annotation of the final masked genome assembly was conducted through EVidenceModeler (EVM; version r03062010) incorporating combined evidences derived from transcriptome alignments, protein alignments, and *ab initio* gene predictions. Contigs from the rubber tree transcriptome assembly were aligned to the genome by PASA (version r09162010) [66] and Exonerate (version 2.2.0) [67]. Plant assembled unique transcripts (PUTs) obtained from PlantGDB [68] were aligned to the genome by GMAP (version 20100727) [69]. Plant protein sequences from genome sequencing projects obtained from the PlantGDB database were aligned to the genome using AAT (version 1.52) [70] and BLAT (version 34) [71]. Contigs from the rubber tree transcriptome assembly was used as training set for training *ab initio* gene prediction software Fgenesh [72]. The rubber tree PASA transcriptome alignment assembly was used as training set for *ab initio* gene prediction softwares Augustus (version 2.5) [73], GlimmerHMM (version 3.0.1) [74], and SNAP (version 2010-07-28) [75]. *Ab initio* gene prediction softwares GeneMarkHMME (version 3) [76] trained with *Arabidopsis thaliana* and Geneid (version 1.4.4) [77] trained with *Cucumis melo* were included into the gene prediction process. Estimation of weights of evidences was performed using EVM with contigs of the rubber tree transcriptome assembly as criterion. In consideration of the general expectation on software performance, weight estimates, and availability of rubber tree specific training, weights of evidences were manually set for the masked assembly as: rubber tree transcriptome assembly, PASA 1, Exonerate 0.5; plant PUTs, GMAP 0.2; plant proteins, AAT 0.2, BLAT 0.2; *ab initio* gene predictions, Fgenesh 0.6, Augustus 0.5, SNAP 0.3, GlimmerHMM 0.3, Geneid 0.2, GeneMarkHMME 0.2.

To ensure quality and refined annotation, several criteria of validation and manual curation were set on top of the common procedure for functional annotation. Protein sequences were functionally annotated through BLASTP searches with E-value cutoff of 10^-5^ against Swiss-Prot, TrEMBL, PlantGDB, UniRef100, NCBI non-redundant database, STRING (version 8.3) [78], and KEGG GENES. Function associated with the putative ORFs was screened with cutoff at least 70% length coverage and 70% similarity. Those that eluded the second stage were further scanned for domain detection by InterPro, PANTHER [79], PRINTS [80], PROSITE [81] patterns, Pfam, and SMART [82] and further curated by alignment against known well-annotated sequence templates such as *A. thaliana* and *R. communis*. Functional annotation was further classified through best reciprocal ortholog match against the curated plant specific database using Pathway Studio (Ariadne Genomics Inc.). EC assignment was obtained using Pathway Studio functional class and KEGG orthologs assessment. KOG assignment was extracted from BLASTP hits of STRING and GENES. GO assignment was extracted from searching results of the InterPro database. Manual curation was performed by comparison of the proteins to PlantRefSeq, KEGG, Swiss-Prot, and InterPro. More than 10,000 proteins were curated with their respective functions. The comparison of identity percentage, bit-score, and length coverage together with rest of the analysis were used for *in silico* designation of the putative function of a specific ORF.

tRNAscan-SE v.1.23 was used with relaxed settings for EufindtRNA (Int Cutoff = −32.1) to identify tRNA genes in the assembly [83]. rRNA genes were identified by aligning the 5S, 5.8S, 18S, and 28S rRNA from *Arabidopsis* and *Oryza* against the assembly using BLASTN 2.2.24 (at least 80% coverage, 50% identity) [84]. Signal peptides in the assembly were identified with SignalP 4.0 [85].

### Identification and Annotation of gene families in *H. brasiliensis*

Genes related to rubber biosynthesis, lignocellulose biosynthesis, systemic acquired resistance, hypersensitive response, pathogenesis related proteins, allergens, transcription factors, phytohormone metabolism and signaling, circadian clock and light signaling, F-box, and carotenoid biosynthesis were identified using CLC software with appropriate template sequences. The identified genes were annotated by BLASTX search of PlantGDB, UniProtKB/TrEMBL, and Plant_refseq protein database with an E-value < 10^-5^.

### Identification and Annotation of NBS-LRR gene families

*H. brasiliensis* proteins with coverage of more than 90% of *A. thaliana* NBS-LRR proteins extracted from PlantGDB, NCBI, and TAIR were sorted based on BLASTP with E-value < 10^-5^, with further confirmations from NCBI Conserved Domain Database domain hits. Annotated gene models from the *H. brasiliensis* assembly were scanned and searched for Pfam, InterPro, and HMMPanther IDs corresponding to the respective motifs, as follows: TIR [PF01582; IPR000157], NBS [PF0931; IPR002182], TIR-NBS [PF01582, PF00931; IPR000157, IPR002182], NBS-LRR [PF0931, PF00560, PF07723, PF07725; PTHR23155:SF236], TIR-NBS-LRR [PF01582, PF0931, PF00560, PF07723, PF07725; IPR000157, IPR002182, IPR001611, IPR011713; PTHR23155:SF300], CC-NBS-LRR [PTHR23155:SF231] and the three types of LRR as LRR_1 [PF00560; IPR001611], LRR_2 [PF07723] and LRR_3 [PF07725; IPR011713]. The presence of coiled-coil (CC) domains was discovered by running through the COILS program [86]. Upon pooling, manual verification and inspection of truncated hits, 618 NBS-LRR genes were identified in *H. brasiliensis*.

### Comparison of rubber biosynthesis-related genes with guayule ESTs

The *H. brasiliensis* genes related to rubber biosynthesis were translated to protein sequences and used as query for TBLASTN analysis against the *P. argentatum* ESTs [GenBank:GW775573–GW787311]. Results were filtered with E-value cutoff of 10^-5^.

### Pathway reconstruction

Metabolic pathways were reconstructed with Pathway Studio software (Ariadne Genomics Inc.) based on Resnet-Plant 3.0 database and Metabolic Pathway Databases (MPW) [87]. Resnet-Plant 3.0 database from Ariadne Genomics contains a collection of 303 metabolic pathways imported from AraCyc 4.0. Pathways are represented as a collection of functional classes (enzymes) and a set of corresponding chemical reactions. Every functional class in the database can contain an unlimited number of protein members encoding corresponding enzymatic activity. Usually a set of members includes paralogs of catalytic and regulatory subunits necessary to perform enzymatic activity.

Manual population of functional classes by protein members represents the initial reconstruction of metabolic pathways in Pathway Studio. The process is equivalent of closing gaps in a metabolic network. After annotation of proteins in Resnet-Plant 3.0 database with rubber genome identifiers and deleting non-rubber proteins, 311 functional classes did not have any members. We used TBLASTN against assembled DNA sequences of the rubber genome to manually identify proteins missed by automatic annotation by orthologs identified with best reciprocal hit method from BLASTP results. The typical workflow for closing gaps in the rubber metabolic network involved downloading protein sequences that could perform the missing enzymatic activity either from *Arabidopsis* or other plant or bacterial genome and then using it as query for TBLASTN. Both GenBank and UniProt were used as sources for protein sequences. Additional pathways present only in RiceCyc or PoplarCyc were identified by comparison of pathway names with those in AraCyc. Pathways missing in AraCyc were added manually to the *Hevea* database in Pathway Studio. MPW was also used in the reconstruction of the rubber biosynthesis pathway.

### Anchoring scaffolds into the linkage map

Based on the published linkage map [17], scaffolds were anchored and oriented into 18 linkage groups. Sequences for 154 microsatellite markers were obtained from public databases. Respective scaffolds were identified by BLAST analysis of the markers against all scaffolds. Gene models were identified and anchored into the corresponding position in the linkage group. When more than one marker was present in the scaffold, genes could be anchored in the correct orientation and in others, the orientation was uncertain. Additional markers located in the scaffolds were identified by BLAST analysis of the whole scaffolds against GenBank.

### Analysis of unique and shared gene clusters

The OrthoMCL pipeline [88] was used to identify and estimate the number of paralogous and orthologous gene clusters within Euphorbiaceae and across various plant groups. Standard settings (BLASTP, E-value < 10^-5^) were used to compute the all-against-all similarities.

### Phylogenetic analysis

A phylogenetic tree was constructed with 17 sequenced genomes (*Chlamydomonas reinhardtii*, *Selaginella moellendorffii*, *Zea mays*, *Oryza sativa*, *Brachypodium distachyon*, *Solanum tuberosum*, *Vitis vinifera*, *Carica papaya*, *A. thaliana*, *P. trichocarpa*, *J. curcas*, *R. communis*, *H. brasiliensis*, *M. esculenta*, *Fragaria vesca*, *Gycine max*, and *Cucumis sativus*). Protein sequences were subjected to all-versus-all BLAST with E-value cutoff of 10^-5^. From the BLAST result, percentage identity was calculated. Inparalogs, orthologs, and co-orthologs were identified using OrthoMCL. Potential inparalog pairs were determined by finding all pairs of proteins within a species that have mutual hits that are better or equal to all of those proteins' hits to proteins in other species. All potential ortholog pairs were determined by finding all pairs of proteins across two species that have hits as good as or better than any other hits between these proteins and other proteins in those species. Potential co-ortholog pairs were determined by finding all pairs of proteins across two species that are connected through orthology and inparology. Each group of proteins with its all inparalogs and orthologs were clustered by MCL program, which generated 57,250 clusters. Clusters which did not have all 17 members were rejected, which yielded 1,364 clusters. They were further filtered by selecting only clusters having single copy in at least 14 out of the 17 plants selected, which finally yielded 144 clusters. Sequences were aligned with ClustalX with gap opening = 10 and gap extension = 0.1 gonnet series matrix. Gblocks was used to extract the conserved blocks in the alignment. From the Gblocks output, various software for phylogenetic tree were used according to maximum likelihood using PhyML [89] with tree improvement method using best of SPR and NNI. Bootstrapping procedure was applied over random 100 replicates and 7 seeds.

### Wet-lab validation of genes

Total RNA was isolated from the young leaves and latex of *H. brasiliensis* RRIM 600 using the RNeasy Mini Kit (Qiagen) according to the manufacturer’s instructions. First-strand cDNA was synthesized using the SuperScript VILO cDNA Synthesis Kit (Invitrogen). The genes were amplified from the cDNA by PCR using gene specific primers. The purified PCR products were cloned into either the pCR4Blunt-TOPO (Invitrogen) or pJET1.2/blunt (Fermentas) vectors and sequenced. This was performed for genes related to rubber biosynthesis, lignin biosynthesis, disease resitance, allergens, transcription factors, and phytohormone biosynthesis.

### Data accessibility

This Whole Genome Shotgun project has been deposited at DDBJ/EMBL/GenBank under the accession [GenBank:AJJZ00000000]. The version described in this paper is the first version, [GenBank:AJJZ01000000]. Assembled transcripts have been deposited in the NCBI Transcriptome Shotgun Assembly database under accession numbers [GenBank:JT914190–JT981478]. The Rubber Genome Browser is available at [90].

## Abbreviations

CAD: Cinnamyl alcohol dehydrogenase; CC: Coiled-coil; CesA: Cellulose synthase; COMT: Caffeic acid O-methyl transferase; CPT: *Cis*-prenyltransferase; DHDDS: Dehydrodolichyl diphosphate synthase; ERF: Ethylene-responsive element binding factor; EST: Expressed sequence tag; EVM: EVidenceModeler; GO: Gene Ontology; HR: Hypersensitive response; IPP: Isopentenyl diphosphate; KOG: Eukaryotic orthologous groups; LRR: Leucine-rich repeat; LTR: Long terminal repeat; MPW: Metabolic Pathway Database; MT: Monosaccharide transporter; MYA: Million years ago; NBS: Nucleotide-binding site; NR: Natural rubber; NRL: Natural rubber latex; PE: Paired-end; PR: Pathogenesis-related; PUTs: Plant assembled unique transcripts; REF: Rubber elongation factor; SALB: South American Leaf Blight; SAR: Systemic acquired resistance; SRPP: Small rubber particle protein; Sup: Suppressor; SUT: Sucrose transporter; TIR: Toll-interleukin-like receptor; UPPS: Undecaprenyl pyrophosphate synthase; WGS: Whole-genome shotgun.

## Competing interests

The authors declare that they have no competing interests.

## Authors’ contributions

MA and NN conceived the project. JAS, NN, and MA managed the project. LW managed part of the raw data generation. SH, AGY, and RC performed raw data generation. YF and BK worked on genome assembly. YF, AYAR, and AD-L conducted genome annotation. AYAR, LSL, HST, MKLMS, and BST performed manual curation. AY, SYO, FLN, BFK, and TAKF performed pathway analysis. AOU, BBM, GPT, KJ, AYAR, QY, and BJL worked on genome analysis and comparative genomics. AYAR and AD-L constructed the genome browser. AOU, BBM, GPT, KJ, SSB, NAA, and NPM carried out wet-lab validation of pathway-specific genes. AOU, BBM, GPT, KJ, YF, JAS, and MA wrote the manuscript. All authors read and approved the final manuscript.

## Supplementary Material

Additional file 1: Table S1 Construction of genomic libraries, generation and filtering of sequencing data used for genomic assembly. **Table S2.** Scaffolds showing the associated genes anchored on to the respective linkage groups based on the reported molecular markers. **Table S3.** Main classes of repeat elements in the *H. brasiliensis* genome assembly. **Table S4.** Summary statistics of gene models predicted by seven programs. **Table S5.** Comparison of publicly available *H. brasiliensis* transcripts with the genome. **Table S6.** General features of the transcriptome assembly. **Table S7.** Functional annotation of predicted proteins for *H. brasiliensis*. **Table S8.** Comparison of KOG functions across various sequenced plant genomes. **Table S9.** Pfam domains in the *H. brasiliensis* genome. **Table S10.** Predicted subcellular localization of *H. brasiliensis* gene models based on SignalP 3.0 analysis. **Table S11.** tRNA types found in the *H. brasiliensis* genome. **Table S12.** Gene Ontology (GO) analysis of *Hevea* specific genes. **Table S13.** InterPro domains within the *Hevea* specific lineage. **Table S14.** Pfam domains within the *Hevea* specific lineage. **Table S15.** KOG analysis of *Hevea* specific genes. **Table S16.** Rubber biosynthesis related genes in the *H. brasiliensis* genome. **Table S17.** Rubber biosynthesis related genes of *H. brasiliensis* in comparison to *Parthenium argentatum* (guayule) ESTs. **Table S18.** Lignocellulose biosynthetic genes of *H. brasiliensis* in comparison to other sequenced genomes. **Table S19.** Putative NBS-coding R genes of *H. brasiliensis* in comparsion to other sequenced genomes. **Table S20.** Pathogenesis-related proteins of *H. brasiliensis* in comparison with other genomes. Table S21. Systemic acquired resistance (SAR) and hypersensitive response (HR) related genes found in the *H. brasiliensis* genome. **Table S22.** Latex allergens in the *H. brasiliensis* genome. **Table S23.** Non-latex allergens in the *H. brasiliensis* genome. **Table S24.** Transcription factors present in *H. brasiliensis* in comparison to other sequenced plant genomes. **Table S25.** Genes involved in phytohormone metabolism, signaling and regulatory events represented in the *H. brasiliensis* genome. **Table S26.** Circadian clock and light signaling gene families from *Hevea* in comparison to *Populus* and *Arabidopsis*. **Table S27.** Major genes involved in carotenoid biosynthesis in *H. brasiliensis* and *A. thaliana*. Click here for file

Additional file 2: Figure S1 Linkage map of *H. brasiliensis* showing 18 linkage groups with 143 anchored scaffolds corresponding to the reported 154 microsatellite markers. **Figure S2.** Complete network of rubber biosynthesis in *H. brasiliensis*. **Figure S3.** Lignin biosynthesis. **Figure S4.** Systemic acquired resistance pathway. **Figure S5.** Hypersensitive response. **Figure S6.** Auxin biosynthesis. **Figure S7.** Auxin signaling pathway. **Figure S8.** Zeatin biosynthesis. **Figure S9.** Cytokinin signaling pathway. **Figure S10.** Gibberellin biosynthesis. **Figure S11.** Gibberellin signaling pathway. **Figure S12.** Ethylene biosynthesis. **Figure S13.** Ethylene signaling pathway. **Figure S14.** Brassinosteroid biosynthesis. **Figure S15.** Brassinosteroid signaling pathway. **Figure S16.** Jasmonic acid biosynthesis. **Figure S17.** Salicylic acid biosynthesis. **Figure S18.** Carotenoid biosynthesis. Click here for file
