# Supplementary material for: Draft genome sequence of the rubber tree Hevea brasiliensis
Source: BMC Genomics. 2013 Feb 2;14:75. doi: 10.1186/1471-2164-14-75 (PMC3575267; doi:10.1186/1471-2164-14-75)
Supplement: Additional file 1: Table S1 — Construction of genomic libraries, generation and filtering of sequencing data used for genomic assembly. Table S2. Scaffolds showing the associated genes anchored on to the respective linkage groups based on the reported molecular markers. Table S3. Main classes of repeat elements in the H. brasiliensis genome assembly. Table S4. Summary statistics of gene models predicted by seven programs. Table S5. Comparison of publicly available H. brasiliensis transcripts with the genome. Table S6. General features of the transcriptome assembly. Table S7. Functional annotation of predicted proteins for H. brasiliensis. Table S8. Comparison of KOG functions across various sequenced plant genomes. Table S9. Pfam domains in the H. brasiliensis genome. Table S10. Predicted subcellular localization of H. brasiliensis gene models based on SignalP 3.0 analysis. Table S11. tRNA types found in the H. brasiliensis genome. Table S12. Gene Ontology (GO) analysis of Hevea specific genes. Table S13. InterPro domains within the Hevea specific lineage. Table S14. Pfam domains within the Hevea specific lineage. Table S15. KOG analysis of Hevea specific genes. Table S16. Rubber biosynthesis related genes in the H. brasiliensis genome. Table S17. Rubber biosynthesis related genes of H. brasiliensis in comparison to Parthenium argentatum (guayule) ESTs. Table S18. Lignocellulose biosynthetic genes of H. brasiliensis in comparison to other sequenced genomes. Table S19. Putative NBS-coding R genes of H. brasiliensis in comparsion to other sequenced genomes. Table S20. Pathogenesis-related proteins of H. brasiliensis in comparison with other genomes. Table S21. Systemic acquired resistance (SAR) and hypersensitive response (HR) related genes found in the H. brasiliensis genome. Table S22. Latex allergens in the H. brasiliensis genome. Table S23. Non-latex allergens in the H. brasiliensis genome. Table S24. Transcription factors present in H. brasiliensis in comparison to other sequenced plant genom [file 1471-2164-14-75-S1.pdf]

**Table S1.** Construction of genomic libraries, generation and filtering of sequencing data used for genomic assembly

| Genome sequencing platform | Library | Average length of read (bp) | Total length (Gb) | CLC preliminary assembly | Newbler preliminary assembly | Final assembly |
|---|---|---|---|---|---|---|
| Illumina | fragment size 200 bp; paired end | 100 | 44.2 | building basic contigs; connecting contigs | not used | not used |
| Illumina (filtered)* | fragment size 200 bp; paired end | 100 | 5.33 | not used | not used | building contigs; constructing scaffolds |
| Illumina (filtered)* | fragment size 200 bp; single end | 100 | 2.05 | not used | not used | building contigs |
| Illumina | fragment size 500 bp; paired end | 100 | 26.9 | extending contigs; connecting contigs | not used | not used |
| Illumina (filtered)* | fragment size 500 bp; paired end | 80 | 2.30 | not used | not used | building contigs; constructing scaffolds |
| Roche/454 | shotgun; single end | 316 | 8.33 | extending contigs | building contigs | building contigs |
| Roche/454 | fragment size 8 kb; paired end | 158 | 5.69 | extending contigs; connecting contigs | building contigs; constructing scaffolds | building contigs; constructing scaffolds |
| Roche/454 | fragment size 8 kb; single end | 235 | 2.50 | extending contigs | building contigs | building contigs |
| Roche/454 | fragment size 20kb; paired end | 148 | 0.987 | not used | building contigs; constructing scaffolds | building contigs; constructing scaffolds |
| Roche/454 | fragment size 20kb; single end | 229 | 0.587 | not used | building contigs | building contigs |
| SOLiD | fragment size 2 kb; paired end | 41 | 3.81 | connecting contigs | not used | not used |
| SOLiD (filtered)* | fragment size 2 kb; paired end | 50 | 0.0918 | not used | not used | building contigs; constructing scaffolds |

*The Illumina reads were filtered by removing the reads matching repetitive regions, whereas the SOLiD reads were filtered by keeping only the paired reads that were at least 50 bp in length.

**Table S2.** Scaffolds showing the associated genes anchored on to the respective linkage groups based on the reported molecular markers

| | Linkage map-g1 |
|---|---|
| **Scaffold 445443** | |
| | Serine-threonine protein kinase |
| | Conserved hypothetical protein |
| | Conserved hypothetical protein |
| | Conserved hypothetical protein |
| | Conserved hypothetical protein |
| | Conserved hypothetical protein |
| | Pyruvate kinase, putative |
| | Chitinase |
| | Hevamine |
| | Hypothetical protein |
| | Chitinase |
| | Conserved hypothetical protein |
| | Hypothetical protein |
| | Conserved hypothetical protein |
| | Protein dimerization, putative |
| | ATP binding protein, putative |
| | Conserved hypothetical protein |
| | Ubiquitin conjugating enzyme 2 (E2) |
| | Ethylene-responsive transcription factor, putative |
| | Conserved hypothetical protein |
| | Mitochondrial carrier protein, putative |
| | Conserved hypothetical protein |
| | Tetrahydrofolylpolyglutamate synthase, putative |
| | DNA binding protein, putative |
| | WRKY transcription factor, putative |
| | **mHbCIRT9** |
| | **mHbCIRT319** |
| | RNA polymerase sigma factor rpoD, putative |
| | Conserved hypothetical protein |
| **Scaffold 365857** | |
| | Nuclear movement protein nudc, putative |
| | Hypothetical protein |
| | Hypothetical protein |
| | Polygalacturonase non-catalytic subunit AroGP3 precursor, putative |
| | Predicted protein |
| | **mHbCIRA274/380** |
| | Cap binding protein, putative |
| | Cap binding protein, putative |
| | Hypothetical protein |
| | Hypothetical protein |
| | Thump domain protein, putative |

| | |
|---|---|
| | Thump domain protein, putative |
| | Hexokinase, putative |
| | Hexokinase, putative |
| | Leucine-rich repeat containing protein, putative |
| **Scaffold 180784** | |
| | Trithorax, putative |
| | Hypothetical protein |
| | Hypothetical protein |
| | Hypothetical protein |
| | ATP binding protein, putative |
| | Splicing factor 3a, putative |
| | Glutamate synthase, putative |
| | Glutamate synthase, putative |
| | Hypothetical protein |
| | Conserved hypothetical protein |
| | Receptor serine/threonine kinase, putative |
| | Hypothetical protein |
| | Brassinosteroid Insensitive 1-associated receptor kinase 1 precursor, putative |
| | Receptor serine/threonine kinase, putative |
| | Receptor serine/threonine kinase, putative |
| | Hypothetical protein |
| | Conserved hypothetical protein |
| | Brassinosteroid Insensitive 1-associated receptor kinase 1 precursor, putative |
| | Receptor serine/threonine kinase, putative |
| | Receptor serine/threonine kinase, putative |
| | Hypothetical protein |
| | **mHbCIRA134** |
| | Conserved hypothetical protein |
| | Conserved hypothetical protein |
| **Scaffold 08981** | |
| | **hmct22** |
| | **hmct21** |
| | Elongation factor 1-beta, putative |
| | ATP binding protein, putative |
| | ATP binding protein, putative |
| | ATP binding protein, putative |
| | Hypothetical protein |
| | Conserved oligomeric Golgi complex component, putative |
| | Conserved oligomeric Golgi complex component, putative |
| | Polcalcin Jun o, putative |
| | Cycloeucalenol cycloisomerase, putative |
| | Cycloeucalenol cycloisomerase, putative |
| | Leucine-rich repeat protein, putative |
| | Nucleolar protein nop56, putative |
| | Adenylate kinase 1, putative |
| | Aspartyl aminopeptidase, putative |

| | |
|---|---|
| | Predicted protein |
| | **mHbCIRA2684** |
| | Conserved hypothetical protein |
| **Scaffold 175245** | |
| | **mHbCIRTAs2455** |
| **Scaffold 125722** | |
| | **mHbCIRTAs2557** |
| | Hypothetical protein |
| **Scaffold 416894** | |
| | Conserved hypothetical protein |
| | Hypothetical protein |
| | Hypothetical protein |
| | **mHbCIRA200** |
| **Scaffold 412180** | |
| | Conserved hypothetical protein |
| | Conserved hypothetical protein |
| | Conserved hypothetical protein |
| | Glutamate decarboxylase, putative |
| | **mHbCIRA 2748** |
| | 1-aminocyclopropane-1-carboxylate deaminase, putative |
| | 1-aminocyclopropane-1-carboxylate deaminase, putative |
| | Conserved hypothetical protein |
| | Homeodomain-leucine zipper protein hd4 |
| | Organic anion transporter, putative |
| | Organic anion transporter, putative |
| | **Linkage map-g2** |
| **Scaffold 416254** | |
| | Protein P21, putative |
| | Conserved hypothetical protein |
| | RNA polymerase II ctd phosphatase, putative |
| | Predicted protein |
| | Conserved hypothetical protein |
| | Predicted protein |
| | RNA binding protein, putative |
| | RNA binding protein, putative |
| | Hypothetical protein |
| | Serine carboxypeptidase, putative |
| | Predicted protein |
| | Pentatricopeptide repeat-containing protein, putative |
| | Pentatricopeptide repeat-containing protein, putative |
| | Conserved hypothetical protein |
| | Predicted protein |
| | ATP binding protein, putative |
| | ATP binding protein, putative |
| | **mHbCIRA 2348** |
| | Predicted protein |

| | |
|---|---|
| | Conserved hypothetical protein |
| | Histone-lysine n-methyltransferase, suvh, putative |
| | Conserved hypothetical protein |
| | RNA-binding protein, putative |
| | Hypothetical protein |
| | Conserved hypothetical protein |
| | AP2 domain transcription factor RAP2.3, putative |
| | AP2 domain transcription factor RAP2.3, putative |
| | Zinc finger protein, putative |
| | Predicted protein |
| | Ubiquinol-cytochrome C reductase iron-sulfur subunit, putative |
| | Aspartate semialdehyde dehydrogenase, putative |
| | Predicted protein |
| | Transaldolase, putative |
| | Cytochrome P450, putative |
| | Predicted protein |
| | Pentatricopeptide repeat-containing protein, putative |
| | Predicted protein |
| | Predicted protein |
| | Transferase, transferring glycosyl groups, putative |
| | Conserved hypothetical protein |
| | Conserved hypothetical protein |
| | 40S ribosomal protein S27, putative |
| | Diphosphoinositol polyphosphate phosphohydrolase, putative |
| **Scaffold 272663** | |
| | Cationic amino acid transporter, putative |
| | Cationic amino acid transporter, putative |
| | BTB and MATH domain-containing protein, putative |
| | BTB and MATH domain-containing protein, putative |
| | Conserved hypothetical protein |
| | Conserved hypothetical protein |
| | Sterol regulatory element-binding protein site 2 protease, putative |
| | Set domain protein, putative |
| | LOB domain-containing protein, putative |
| | Histone ubiquitination proteins group (HUPB902) |
| | Conserved hypothetical protein |
| | Membrin, putative |
| | HSP90 co-chaperone, putative |
| | Conserved hypothetical protein |
| | BTB/POZ domain-containing protein KCTD9, putative |
| | BTB/POZ domain-containing protein KCTD9, putative |
| | Serine/threonine-protein kinase ripk4, putative |
| | Vitamin-b12 independent methionine synthase |
| | 5-methyltetrahydropteroyltriglutamate |
| | Predicted protein |
| | Early nodulin 55-2 precursor, putative |

| | |
|---|---|
| | Early nodulin 55-2 precursor, putative |
| | Early nodulin 55-2 precursor, putative |
| | Early nodulin 55-2 precursor, putative |
| | Mavicyanin, putative |
| | Predicted protein |
| | Conserved hypothetical protein |
| | Annexin, putative |
| | Predicted protein |
| | Conserved hypothetical protein |
| | **gSSR096** |
| | Conserved hypothetical protein |
| | Conserved hypothetical protein |
| | Conserved hypothetical protein |
| | Hydrolase, hydrolyzing O-glycosyl compounds, putative |
| | Hydrolase, hydrolyzing O-glycosyl compounds, putative |
| | ATECP63, putative |
| | Syntaxin, putative |
| | Syntaxin, putative |
| | Predicted protein |
| | **hmct4** |
| | Conserved hypothetical protein |
| | Catalytic, putative |
| | Importin beta-3, putative |
| | **mHbCIRA2534** |
| | Importin beta-3, putative |
| | Conserved hypothetical protein |
| | Hypothetical protein |
| | Tubulin alpha chain, putative |
| | Eukaryotic translation initiation factor 4e type, putative |
| | Predicted protein |
| | Conserved hypothetical protein |
| | Conserved hypothetical protein |
| | Adenylsulfate kinase, putative |
| **Scaffold 335127** | |
| | AMP dependent ligase, putative |
| | AMP dependent ligase, putative |
| | Conserved hypothetical protein |
| | **mHbCIRA 2368** |
| | Transcription regulator, putative |
| | Transferase, putative |
| | Transferase, putative |
| | 3'-N-debenzoyl-2'-deoxytaxol N-benzoyltransferase, putative |
| | Predicted protein |
| **Scaffold 586014** | |
| | **mHbCIRA2734** |

| | |
|---|---|
| **Scaffold 59282** | |
| | **mHbCIRA2510** |
| | Hypothetical protein |
| | Expressed protein, putative |
| | Blue copper protein precursor, putative |
| | Conserved hypothetical protein |
| | Hypothetical protein |
| | 26S proteasome regulatory subunit S3, putative |
| | Predicted protein |
| | Predicted protein |
| | Predicted protein |
| | Predicted protein |
| | Hypothetical protein |
| **Scaffold 421682** | |
| | **mHbCIRT2140** |
| | DNA-binding protein MNB1B, putative |
| | Protein binding protein, putative |
| | Conserved hypothetical protein |
| | Predicted protein |
| | Vacuolar cation/proton exchanger 1a, putative |
| | Conserved hypothetical protein |
| | *Hevea brasiliensis* isolate SSH47 sequence |
| | Predicted protein |
| | Conserved hypothetical protein |
| | Ubiquitin-protein ligase, putative |
| | Ethylene-inducible protein (ER1) |
| | Symplekin, putative |
| | **hmct40** |
| | Predicted protein |
| **Scaffold 349088** | |
| | Hypothetical protein |
| | Predicted protein |
| | **mHbCIRA2381** |
| | **mHbCIRA2326** |
| | Conserved hypothetical protein |
| | Conserved hypothetical protein |
| **Scaffold 222405** | |
| | **mHbCIRt283** |
| | **Linkage map-g3** |
| **Scaffold 174498** | |
| | DNA-directed RNA polymerase, putative |
| | **mHbCIRA278** |
| **Scaffold 453132** | |
| | Predicted protein |
| | Hypothetical protein |
| | DNA-repair protein xrcc1, putative |

| | |
|---|---|
| | NMDA receptor-regulated protein, putative |
| | NMDA receptor-regulated protein, putative |
| | Ring finger protein, putative |
| | Cysteine protease, putative |
| | Cysteine protease, putative |
| | Hypothetical protein |
| | Myristoyl-acyl carrier protein thioesterase, chloroplast precursor, putative |
| | Myristoyl-acyl carrier protein thioesterase, chloroplast precursor, putative |
| | ATP-dependent transporter, putative |
| | Auxin-responsive protein IAA1, putative |
| | Hypothetical protein |
| | Hypothetical protein |
| | Predicted protein |
| | Glutathione S-transferase omega |
| | Oxalate/formate antiporter |
| | Aspartate aminotransferase, putative |
| | **mHbCIRTAs2558** |
| | Aspartate aminotransferase, putative |
| | Katanin p60 ATPase-containing subunit, putative |
| | **gHbCIRA2415** |
| | Katanin p60 ATPase-containing subunit |
| **Scaffold 263548** | |
| | **gHbCIRA2516** |
| | **mHbCIRA156** |
| | Transportin-3, putative |
| **Scaffold 93599** | |
| | Predicted protein |
| | Electron transporter, putative |
| | **hmct21** |
| | **hmct22** |
| | **mHbCIRA2485** |
| | Hypothetical Protein |
| **Scaffold 305576** | |
| | **hmct22** |
| | **hmct21** |
| | **mHbCIRTAs2697** |
| **Scaffold 198002** | |
| | Conserved Hypothetical Protein |
| | **mHbCIRBAC123d05** |
| | **hmct43** |
| | Conserved hypothetical protein |
| | Conserved hypothetical protein |
| **Scaffold 181532** | |
| | **mHbCIRA2264** |
| | Calreticulin-3-like |

| | |
|---|---|
| **Scaffold 170728** | |
| | Transcription factor RF2a, putative |
| | Electron transfer flavoprotein-ubiquinone oxidoreductase |
| | **mHbCIRA2707** |
| | Electron transfer flavoprotein-ubiquinone oxidoreductase, putative |
| | Predicted protein strictosidine synthase, putative |
| **Scaffold 197224** | |
| | Protein binding protein, putative |
| | Predicted protein |
| | Phospholipid:diacylglycerol acyltransferase 2 (PDAT2) |
| | RNA splicing protein mrs2, mitochondrial, putative |
| | **mHbCIRA2749** |
| | srpk, putative |
| | **Linkage map-g4** |
| **Scaffold 131572** | |
| | Ethylene-responsive element binding protein 1 (EREBP1) |
| | FAD NAD binding oxidoreductases, putative |
| | AP2/ERF domain-containing transcription factor (ERF43) |
| | Serine-threonine protein kinase, plant-type, putative |
| | **mHbCIRA2706** |
| | Predicted protein |
| | Auxin-repressed protein-like protein ARP1 |
| | Ring finger protein, putative |
| **Scaffold 138950** | |
| | Receptor protein kinase CLAVATA1 precursor, putative |
| | Conserved hypothetical protein |
| | **mHbCIRTA2157** |
| | Conserved hypothetical protein |
| | **mHbCIRT2091** |
| | Conserved hypothetical protein |
| | Protein binding protein, putative |
| | Predicted protein |
| | Predicted protein |
| **Scaffold 541370** | |
| | **mHbCIRA2766** |
| **Scaffold 430947** | |
| | DNA repair protein reca, putative |
| | DNA repair protein reca, putative |
| | Nucleosome assembly protein, putative |
| | Hypothetical protein |
| | Regulatory protein NPR1, putative |
| | Hypothetical Protein |
| | Calmodulin binding protein, putative |
| | **mHbCIRA385** |
| | Hypothetical Protein |
| | Casein kinase, putative |

| | |
|---|---|
| | Conserved hypothetical protein |
| | Diacylglycerol acyltransferase 1 (DGAT-1) |
| | Dead box ATP-dependent RNA helicase, putative |
| | n-myristoyl transferase, putative |
| | DNA binding protein, putative |
| | Lycopene epsilon cyclase, chloroplast precursor, putative |
| | Lycopene epsilon cyclase, chloroplast precursor, putative |
| | Predicted protein |
| | Conserved hypothetical protein |
| **Scaffold 287934** | |
| | S-receptor kinase, putative |
| | L-ascorbate peroxidase 1, cytosolic, putative |
| | Conserved hypothetical protein |
| | grr1, plant, putative |
| | Conserved hypothetical protein |
| | Phopholipase d alpha, putative |
| | **Hbtnr-50** |
| | DNA binding protein, putative |
| | Multidrug resistance pump, putative |
| | Beta-glucosidase, putative |
| | RNA-dependent RNA polymerase, putative |
| | Xenotropic and polytropic murine leukemia virus receptor ids-4, putative |
| | Conserved hypothetical protein |
| | Predicted protein |
| | Conserved hypothetical protein |
| | Conserved hypothetical protein |
| | Triose phosphate chloroplast precursor, putative |
| | **mbHCIRTAs2277** |
| | ATPase n2b, putative |
| | Predicted protein |
| | ADP,ATP carrier protein, putative |
| | ADP,ATP carrier protein, putative |
| | Conserved hypothetical protein |
| | Cellulose synthase A catalytic subunit 6 [UDP-forming], putative |
| | **Hbtnr-75** |
| | **Hbtnr-101** |
| | Conserved hypothetical protein |
| | Conserved hypothetical protein |
| | Hypothetical protein |
| | Protein With Unknown Function |
| | tso1, putative |
| | Acyl-protein thioesterase, putative |
| | **mHbCIRt93** |
| | Peptidyl-prolyl cis-trans isomerase, putative |
| | Ammonium transporter, putative |
| | DNA (cytosine-5)-methyltransferase, putative |

| | |
|---|---|
| | Arabidopsis lyrata subsp. lyrata hypothetical protein |
| | Hypothetical protein |
| | Predicted Protein |
| | ran, putative |
| | Conserved hypothetical protein |
| | Ubiquinol-cytochrome C reductase complex 14 kda protein |
| | Potassium channel AKT6, putative |
| | Boron transporter, putative |
| | Conserved hypothetical protein |
| | WD-repeat protein, putative |
| | Conserved hypothetical protein |
| | Conserved hypothetical protein |
| | Conserved hypothetical protein |
| | Anthocyanidin synthase (ANS1) |
| | Glycerate dehydrogenase, putative |
| | **Linkage map-g5** |
| **Scaffold 123369** | |
| | Arginine/Serine-rich splicing factor, putative |
| | CCD1, putative |
| | FAD NAD binding oxidoreductases, putative |
| | Kinase, putative |
| | Kinase, putative |
| | Conserved hypothetical protein |
| | Predicted protein |
| | Ubiquitin-protein ligase, putative |
| | ORMDL, putative |
| | Predicted protein |
| | WD-repeat protein, putative |
| | **mHbCIRa460** |
| | Predicted protein (MYB201) |
| | Predicted protein |
| | Laccase, putative |
| | Laccase, putative |
| | Conserved hypothetical protein |
| | Beta-fructofuranosidase, putative |
| | Beta-fructofuranosidase, putative |
| | Beta-fructofuranosidase, putative |
| | Conserved hypothetical protein |
| | Conserved hypothetical protein |
| | Auxin response factor, putative |
| | Importin-alpha re-exporter, putative |
| | AP2/ERF domain-containing transcription factor (DREB56) |
| | Conserved hypothetical protein |
| | DNA binding protein, putative |
| | Predicted protein |
| | Flavonoid 3-hydroxylase, putative |

| | |
|---|---|
| | **gHbCIRA2737** |
| | Aspartic proteinase nepenthesin-1 precursor, putative |
| | Ubiquitin ligase SINAT3, putative |
| | Ubiquitin ligase SINAT3, putative |
| | Dynamin, putative |
| | Conserved hypothetical protein |
| | Conserved hypothetical protein |
| | Conserved hypothetical protein |
| | Dopamine beta-monooxygenase, putative |
| | Eukaryotic translation initiation factor 3 subunit, putative |
| | Eukaryotic translation initiation factor 3 subunit, putative |
| | **gHbCIRT2636** |
| | Trithorax, putative |
| | RING-H2 finger protein ATL3K, putative |
| | RING-H2 finger protein ATL3K, putative |
| | **mbHCIRTAs 2559** |
| | Hypothetical protein |
| | Beclin-1, putative |
| | Predicted protein |
| | Hypothetical protein |
| | Cyclic nucleotide-gated ion channel, putative |
| | Predicted protein |
| | Dead box ATP-dependent RNA helicase, putative |
| | Hypothetical protein |
| | Triacylglycerol lipase, putative |
| | Conserved hypothetical protein |
| | Pentatricopeptide repeat-containing protein, putative |
| | Chitinase, putative |
| | Chitinase, putative |
| | Histone H3 (HTR904) |
| **Scaffold 274716** | |
| | Pectinesterase-2 precursor, putative |
| | Pectinesterase, putative |
| | Pentatricopeptide repeat-containing protein, putative |
| | Carboxypeptidase regulatory region-containing protein, putative |
| | Carboxypeptidase regulatory region-containing protein, putative |
| | Conserved hypothetical protein |
| | Hypothetical protein |
| | Conserved hypothetical protein |
| | **mHbCIRTAs2186** |
| | Kinase, putative |
| | Enzyme inhibitor, putative |
| | Protein binding protein, putative |
| | Predicted protein |
| | WRKY transcription factor 3 |

| | |
|---|---|
| **Scaffold 127481** | |
| | **mHbCIRA36** |
| **Scaffold 297292** | |
| | **mHbCIRA 2745** |
| | Carboxy-terminal kinesin, putative |
| | Carboxy-terminal kinesin, putative |
| | **gHbCIRTAs2724** |
| **Scaffold 129112** | |
| | **mHbCIRA2509** |
| | Serine-threonine protein kinase, plant-type, putative |
| **Scaffold 25532** | |
| | **mHbCIRA2518** |
| **Scaffold 53705** | |
| | Methylcobalamin:com methyltransferase, putative |
| | **mHbCIRT2603** |
| | **Linkage map-g6** |
| **Scaffold 227539** | |
| | **mHbCIRa296** |
| | ATP binding protein, putative |
| **Scaffold 588071** | |
| | Hypothetical protein |
| | Type II inositol 5-phosphatase, putative |
| | Beta-hexosaminidase, putative |
| | Predicted protein |
| | **mHbCIRa 367** |
| | Pentatricopeptide repeat-containing protein, putative |
| | Pentatricopeptide repeat-containing protein, putative |
| | Serine-threonine protein kinase, plant-type, putative |
| | Endo-1,4-beta-glucanase, putative |
| | Latex cyanogenic beta glucosidase |
| | Latex cyanogenic beta glucosidase |
| | Type II inositol 5-phosphatase, putative |
| | Predicted protein |
| | Pentatricopeptide repeat-containing protein, putative |
| | Type II inositol 5-phosphatase, putative |
| | Cohesin subunit rad21, putative |
| | **mHbCIRa2391** |
| | Splicing factor U2AF-associated protein, putative |
| | Latex cyanogenic beta glucosidase |
| | Type II inositol 5-phosphatase, putative |
| | Predicted protein |
| | Beta-hexosaminidase, putative |
| | Acetolactate synthase, putative |
| | Leucine zipper-ef-hand containing transmembrane protein, putative |
| | Cohesin subunit rad21, putative |

| | |
|---|---|
| **Scaffold 163591** | |
| | Signal recognition particle receptor subunit beta, putative |
| | Conserved hypothetical protein |
| | Conserved hypothetical protein |
| | Axial regulator YABBY4, putative |
| | Hypothetical protein |
| | ATP binding protein, putative |
| | Nucleic acid binding protein, putative |
| | Conserved hypothetical protein |
| | Conserved hypothetical protein |
| | Pyroglutamyl-peptidase I, putative |
| | Pentatricopeptide repeat-containing protein, putative |
| | Polygalacturonase precursor, putative |
| | **mHbCIRa 170** |
| | Polygalacturonase, putative |
| | Phosphatidylinositol 3-kinase class, putative |
| | Phosphatidylinositol 3-kinase class, putative |
| | Phosphatidylinositol 3-kinase class, putative |
| **Scaffold 228549** | |
| | Predicted protein (MYB) |
| | Predicted protein |
| | **mHbCIRA2498** |
| | ATATH13, putative |
| | Conserved hypothetical protein |
| | Conserved hypothetical protein |
| | Nuclease, putative |
| | Nucleoside transporter, putative |
| | Conserved hypothetical protein |
| | Conserved hypothetical protein |
| | Predicted protein |
| | Splicing endonuclease positive effector sen1, putative |
| | Conserved hypothetical protein |
| | Conserved hypothetical protein |
| | Glutaredoxin S12 |
| | Monooxygenase, putative |
| | Monooxygenase, putative |
| | Myosin XI, putative |
| **Scaffold 186880** | |
| | **mHbCIRA2398** |
| **Scaffold 187443** | |
| | **mHbCIRA2511** |
| **Scaffold 114815** | |
| | Structural constituent of cell wall, putative |
| | Predicted protein |
| | Magnesium-dependent phosphatase 1-like |
| | ATP binding protein, putative |

| | |
|---|---|
| | Hypothetical protein |
| | Conserved hypothetical protein |
| | Conserved hypothetical protein |
| | Conserved hypothetical protein |
| | Conserved hypothetical protein |
| | Conserved hypothetical protein |
| | ATP binding protein, putative |
| | Cysteine-type endopeptidase, putative |
| | **mHbCIRA2406** |
| | ATP binding protein, putative |
| | ATP binding protein, putative |
| | Conserved hypothetical protein |
| | Hypothetical protein |
| | Conserved hypothetical protein |
| | **Linkage map-g7** |
| **Scaffold 122409** | |
| | Serine/threonine protein kinase, putative |
| | Ubiquitin-protein ligase, putative |
| | **mHbCIRTAs2743** |
| | Protein G10, putative |
| | ATP-binding cassette transporter, putative |
| | ATP-binding cassette transporter, putative |
| | Starch branching enzyme II, putative |
| | Starch branching enzyme II, putative |
| | **gHbCIRA2758** |
| | Conserved hypothetical protein |
| | Predicted protein |
| **Scaffold 224310** | |
| | **mHbCIRA2708** |
| **Scaffold 146047** | |
| | tRNA (guanine-n(7)-)-methyltransferase, putative |
| | Remorin, putative |
| | Pentatricopeptide repeat-containing protein, putative |
| | GTP-binding protein typa/bipa, putative |
| | **mHbCIRA2429** |
| | Predicted protein |
| | Calcium-dependent protein kinase (CDPK1) |
| **Scaffold 238809** | |
| | Conserved hypothetical protein |
| | Villin 1-4, putative |
| | **g15mHbCIRA295** |
| | Predicted protein |
| | Insulin-degrading enzyme, putative |
| **Scaffold 416082** | |
| | **gHbCIRA2763** |

| | |
|---|---|
| | Signal peptidase I, putative |
| | **hmct21** |
| **Scaffold 463202** | |
| | Predicted protein |
| | Proliferation-associated 2g4, putative |
| | Proliferation-associated 2g4, putative |
| | **mHbCIRA2686** |
| | Conserved hypothetical protein |
| | Glutaredoxin, grx, putative |
| | Glutaredoxin, grx, putative |
| **Scaffold 519782** | |
| | **mHbCIRT2115** |
| **Scaffold 93102** | |
| | Phospholipase C 3 precursor, putative |
| | Conserved hypothetical protein |
| | Protein binding protein, putative |
| | **mHbCIRA2762** |
| | Deoxyribonuclease, putative |
| | DNA-binding protein smubp-2, putative |
| | Conserved hypothetical protein |
| | Transcription regulator, putative |
| | **Linkage map-g8** |
| **Scaffold 231182** | |
| | **mHbCIRA2442** |
| | Conserved hypothetical protein |
| | Probable membrane-associated kinase regulator 1-like |
| **Scaffold 161238** | |
| | Ubiquitin-associated uba/ubx domain-containing protein, putative |
| | TBC1 domain family member |
| | DUF246 Domain-containing protein At1g04910-like |
| | Conserved hypothetical protein |
| | Pentatricopeptide repeat-containing protein, putative |
| | Anthranilate N-benzoyltransferase protein, putative |
| | Arp2/3 complex 20 kd subunit, putative |
| | Histidine kinase-, DNA gyrase B-, and HSP90-like ATPase family protein (NOV) |
| | **mHbCIRa251** |
| | Histidine kinase-, DNA gyrase B-, and HSP90-like ATPase family protein (NOV) |
| | Alkaline phytoceramidase, putative |
| | Methionine-tRNA synthetase, putative |
| | Beta-galactosidase, putative |
| | Conserved hypothetical protein |
| | Ubiquitin-protein ligase, putative |
| | Conserved hypothetical protein |
| | Non-imprinted in Prader-Willi/Angelman syndrome region protein, putative |
| | Conserved hypothetical protein |
| | Chaperone protein dnaJ 20, chloroplast precursor, putative |

| | |
|---|---|
| | **hmct40** |
| **Scaffold 417274** | |
| | Chromatin remodeling complex subunit (CHR952) |
| | **mHbCIRT73** |
| | Mom(plant), putative |
| | Hypothetical protein |
| | Mom(plant), putative |
| | Conserved hypothetical protein |
| | Glycosyltransferase, cazy family GT8 |
| | Conserved hypothetical protein |
| **Scaffold 354160** | |
| | Nuclear inhibitor of protein phosphatase-1, putative |
| | Nuclear inhibitor of protein phosphatase-1, putative |
| | **mHbCIRT686** |
| | Nuclear inhibitor of protein phosphatase-1, putative |
| **Scaffold 235226** | |
| | Calcium ion binding protein, putative |
| | Glutamate receptor 2 plant, putative |
| | Hypothetical protein |
| | Glutamate receptor 2 plant, putative |
| | Predicted protein |
| | Predicted protein |
| | Homogentisate 1,2-dioxygenase, putative |
| | Hypothetical protein |
| | Ring finger protein, putative |
| | **mHbCIRT2542** |
| | RNA exonuclease, putative |
| **Scaffold 328348** | |
| | Processing protein PRP40, putative |
| | **mHbCIRT2635** |
| | Processing protein PRP40, putative |
| **Scaffold 136822** | |
| | Predicted protein |
| | **mHbCIRa120** |
| | Hypothetical protein |
| **Scaffold 488663** | |
| | **mHbCIRT730** |
| | Phospholipid:diacylglycerol acyltransferase 1-2 (PDAT1-2) |
| **Scaffold 412100** | |
| | Transcription factor, putative |
| | Transcription factor, putative |
| | Transcription factor, putative |
| | **M630** |
| | ATP-dependent clp protease ATP-binding subunit clpx, putative |
| | ATP-dependent clp protease ATP-binding subunit clpx, putative |
| | Transcription factor, putative |

| | |
|---|---|
| | Conserved hypothetical protein |
| | Predicted protein |
| | Nitrate transporter, putative |
| | Nitrate transporter, putative |
| | CDK-activating kinase |
| | cak1, putative |
| | Sugar transporter, putative |
| | Predicted protein |
| | Transcription factor, putative |
| | 3-hydroxyacyl-CoA dehyrogenase, putative |
| | Conserved hypothetical protein |
| | Multidrug resistance pump, putative |
| | Multidrug resistance pump, putative |
| | Conserved hypothetical protein |
| | Aspartate aminotransferase, putative |
| | Predicted protein |
| | Hypothetical protein |
| | Cytoplasmic dynein light chain, putative |
| | Conserved hypothetical protein |
| | Hypothetical protein |
| | Pentatricopeptide repeat-containing protein, putative |
| | **Linkage map-g9** |
| **Scaffold 89544** | |
| | **mHbCIRTA2244** |
| | **hmct40** |
| | **mHbCIRA2298** |
| | DNA binding protein, putative |
| | Homeobox protein, putative |
| **Scaffold 124084** | |
| | Brassinosteroid Insensitive 1-associated receptor kinase 1 precursor, putative |
| | **mHbCIRA2744** |
| **Scaffold 139710** | |
| | Dolichyl-diphosphooligosaccharide--protein glycosyltransferase, putative |
| | tRNA delta(2)-isopentenylpyrophosphate transferase, putative |
| | Lipid binding protein, putative |
| | Lysophosphatidic acid acyltransferase, putative |
| | Conserved hypothetical protein |
| | Conserved hypothetical protein |
| | Multidrug resistance pump, putative |
| | Multidrug resistance pump, putative |
| | 4-hydroxy-3-methylbut-2-enyl diphosphate reductase |
| | NAD dependent epimerase/dehydratase, putative |
| | NAD dependent epimerase/dehydratase, putative |
| | S-adenosylmethionine-dependent methyltransferase, putative |
| | S-adenosylmethionine-dependent methyltransferase, putative |
| | **mHbCIRa104** |

| | |
|---|---|
| | Protein ariadne-1, putative |
| | Conserved hypothetical protein |
| | Non-symbiotic hemoglobin, putative |
| | Non-symbiotic hemoglobin, putative |
| | Conserved hypothetical protein |
| | Hypothetical protein |
| **Scaffold 199300** | |
| | **mHbCIRA2689** |
| **Scaffold 412552** | |
| | Predicted protein |
| | Transcriptional regulator SUPERMAN, putative |
| | **mHbCIRTAs2178** |
| | Transcriptional regulator SUPERMAN, putative |
| | Ethylene receptor, putative |
| | DNA binding protein, putative |
| | Protein transporter, putative |
| | Hypothetical protein |
| | DNA repair helicase rad5,16, putative |
| | DNA repair helicase rad5,16, putative |
| | Conserved hypothetical protein |
| | Conserved hypothetical protein |
| | Desacetoxyvindoline 4-hydroxylase, putative |
| | Outward rectifying K+ channel (KCO1) |
| | 1-aminocyclopropane-1-carboxylate oxidase homolog 12-like |
| | Desacetoxyvindoline 4-hydroxylase, putative |
| | Conserved hypothetical protein |
| | Auxin-induced protein 5NG4, putative |
| | DNA binding protein, putative |
| | Predicted protein |
| **Scaffold 411125** | |
| | ATP binding protein, putative |
| | ATP binding protein, putative |
| | **gHbCIRA2522** |
| | **mHbCIRA2432** |
| | Eukaryotic translation initiation factor 2c, putative |
| | Eukaryotic translation initiation factor 2c, putative |
| **Scaffold 128180** | |
| | ATP binding protein, putative |
| | Conserved hypothetical protein |
| | Conserved hypothetical protein |
| | Zinc finger protein, putative |
| | Predicted protein |
| | Lipase |
| | Predicted protein |
| | Helicase, putative |
| | Receptor serine-threonine protein kinase, putative |

| | |
|---|---|
| | Receptor serine-threonine protein kinase, putative |
| | Glutaredoxin, grx, putative |
| | Acyl-coa binding protein, putative |
| | Anaphase promoting complex subunit, putative |
| | Anaphase promoting complex subunit, putative |
| | Predicted protein |
| | Tonneau, putative |
| | Serine/threonine protein kinase, putative |
| | Spo0B-associated GTP-binding protein, putative |
| | DNA binding protein, putative |
| | Nucleic acid binding protein, putative |
| | Always early, putative |
| | **mHbCIRA2682** |
| | Always early, putative |
| | AMP-activated protein kinase, gamma regulatory subunit, putative |
| | Hypothetical protein |
| | Hypothetical protein |
| | Pentatricopeptide repeat-containing protein, putative |
| | Flavonol synthase/flavanone 3-hydroxylase, putative |
| | Cullin-1, putative |
| | Plastid CUL1 (CUL1) |
| | **Linkage map-g10** |
| **Scaffold 121193** | |
| | **mHbCIRA2408** |
| **Scaffold 431381** | |
| | SAUR family protein (SAUR23) |
| | Calcium-dependent protein kinase, putative |
| | Calcium-dependent protein kinase, putative |
| | Calcium-dependent protein kinase, putative |
| | Conserved hypothetical protein |
| | **mHbCIRA2767** |
| | Conserved hypothetical protein |
| | **gHbCIRA2510** |
| | Predicted protein |
| | Predicted protein |
| | Conserved hypothetical protein |
| | Predicted protein |
| | **hmac3** |
| **Scaffold 601618** | |
| | **Hbtnr-46** |
| | **Hbtnr-91** |
| | Cytochrome P450, putative |
| | Cytochrome P450, putative |
| | Cytochrome P450, putative |
| | Heat shock protein binding protein |
| | Predicted protein |

| | |
|---|---|
| | **gHbCIRA2317** |
| | Amino acid binding protein, putative |
| | **mHbCIRA2320** |
| **Scaffold 106428** | |
| | **mHbCIRA2425** |
| **Scaffold 297920** | |
| | Protein ABIL2, putative |
| | Hypothetical protein |
| | Hypothetical protein |
| | Predicted protein |
| | Cdk8, putative |
| | Transcription factor, putative |
| | **mHbCIRT2096** |
| **Scaffold 410607** | |
| | Transducin/WD40 domain-containing protein-like protein (AT4G14310) |
| | Structural molecule, putative |
| | **mHbCIRa214** |
| | Kinesin heavy chain, putative |
| | Desiccation-associated protein (MTR_2g077860) |
| | Glycolipid transfer protein, putative |
| | Conserved hypothetical protein |
| | 60S ribosomal protein L14, putative |
| | Protein phosphatase 2c, putative |
| | Conserved hypothetical protein |
| | Conserved hypothetical protein |
| | Kinesin light chain, putative |
| | Conserved hypothetical protein |
| | **mHbCIRT2668** |
| **Scaffold 151685** | |
| | **mHbCIRA2316** |
| | WRKY transcription factor, putative |
| **Scaffold 168316** | |
| | Sodium-bile acid cotransporter, putative |
| | **mHbCIRA2388** |
| **Scaffold 237846** | |
| | Vam6/Vps39-like protein-like |
| | Conserved hypothetical protein |
| | Conserved hypothetical protein |
| | Protein FAM96B, putative |
| | Transporter, putative |
| | Chitin-inducible gibberellin-responsive protein, putative |
| | Succinate dehydrogenase, putative |
| | Steroid 22-alpha hydroxylase (DWF4) |
| | Steroid 22-alpha hydroxylase (DWF4) |
| | Hypothetical protein |
| | ISWI chromatin remodeling complex ATPase ISW1, putative |

| | |
|---|---|
| | Conserved hypothetical protein |
| | U1 small nuclear ribonucleoprotein 70 kDa, putative |
| | maf protein, putative |
| | Somatic embryogenesis receptor kinase, putative |
| | Somatic embryogenesis receptor kinase, putative |
| | Somatic embryogenesis receptor kinase, putative |
| | Somatic embryogenesis receptor kinase, putative |
| | Conserved hypothetical protein |
| | Acidic leucine-rich nuclear phosphoprotein 32-related protein-like |
| | Sugar transporter, putative |
| | **mHbCIRA2702** |
| | Predicted protein |
| | **hmct40** |
| | Sugar transporter, putative |
| | Conserved hypothetical protein |
| | Glutamyl-trna synthetase 1, 2, putative |
| | TPR repeat-containing thioredoxin TTL1-like |
| | Tetratricopetide-repeat thioredoxin-like 4 protein (TTL4) |
| **Scaffold 415719** | |
| | ATP-binding cassette transporter, putative |
| | Conserved hypothetical protein |
| | Vacuolar cation/proton exchanger 1a, putative |
| | NudC domain-containing protein, putative |
| | Kinase, putative |
| | ATP binding protein, putative |
| | Conserved hypothetical protein |
| | Cyclophilin, putative |
| | Ubiquitin-protein ligase, putative |
| | Pyridoxin biosynthesis protein PDX1, putative |
| | 1-phosphatidylinositol-4,5-bisphosphate phosphodiesterase, putative |
| | 1-phosphatidylinositol-4,5-bisphosphate phosphodiesterase, putative |
| | 1-phosphatidylinositol-4,5-bisphosphate phosphodiesterase, putative |
| | 1-phosphatidylinositol-4,5-bisphosphate phosphodiesterase, putative |
| | Transcription factor, putative |
| | Conserved hypothetical protein |
| | Omega-3 fatty acid desaturase (Fad3) |
| | Omega-3 fatty acid desaturase, endoplasmic reticulum, putative |
| | Hypothetical protein |
| | Selt-like protein-like |
| | Serine-threonine protein kinase, plant-type, putative |
| | Tonoplast intrinsic protein, putative |
| | Predicted protein |
| | Alpha-galactosidase/alpha-n-acetylgalactosaminidase, putative |
| | Pentatricopeptide repeat-containing protein, putative |
| | Conserved hypothetical protein |
| | Putative integral membrane protein conserved region |

| | |
|---|---|
| | Conserved hypothetical protein |
| | Conserved hypothetical protein |
| | ATP-binding cassette transporter, putative |
| | Histone deacetylase, putative |
| | Histone deacetylase, putative |
| | Protein transport protein sec23, putative |
| | Protein transport protein sec23, putative |
| | **mHbCIRTAs2196** |
| | Protein transport protein sec23, putative |
| | Taz protein, putative |
| | Calmodulin binding protein, putative |
| | Gibberellin 3-beta-dioxygenase, putative |
| | Serine-threonine protein kinase, plant-type, putative |
| | Conserved hypothetical protein |
| | Cucumisin precursor, putative |
| | Cucumisin precursor, putative |
| | Conserved hypothetical protein |
| | Type 2 diacylglycerol acyltransferase (DGAT2) |
| | Type 2 diacylglycerol acyltransferase (DGAT2) |
| | FAR1-RELATED SEQUENCE 5-like |
| | FAR1-RELATED SEQUENCE 5-like |
| | Auxin:hydrogen symporter, putative |
| | Auxin:hydrogen symporter, putative |
| **Scaffold 172714** | |
| | Histidine-containing phosphotransfer protein, putative |
| | Serine-threonine protein kinase, plant-type, putative |
| | Pentatricopeptide repeat-containing protein, putative |
| | Bsu-protein phosphatase, putative |
| | Stromal antigen, putative |
| | Stromal antigen, putative |
| | NPL4-like protein 1-like |
| | GTPase activating protein, putative |
| | Conserved hypothetical protein |
| | RNA binding protein, putative |
| | U1 small nuclear ribonucleoprotein C, putative |
| | **mHbCIRA157** |
| | U1 small nuclear ribonucleoprotein C, putative |
| | F-box/kelch-repeat protein (MTR_2g015760) |
| | Conserved hypothetical protein |
| | Short chain alcohol dehydrogenase, putative |
| | Shikimate dehydrogenase, putative |
| | Predicted protein |
| | Transcription factor, putative |
| | Transcription factor, putative |
| | Serine endopeptidase degp2, putative |
| | Monooxygenase, putative |

| | |
|---|---|
| | Predicted protein |
| | Conserved hypothetical protein |
| **Scaffold 284371** | |
| | **mHbCIRA2392** |
| | Big map kinase/bmk, putative |
| **Scaffold 429519** | |
| | Hypothetical protein |
| | DNA binding protein, putative |
| | Ubiquitin-conjugating enzyme h, putative |
| | Chromo domain protein, putative |
| | Latex beta-cyanoalanine synthase |
| | Conserved hypothetical protein |
| | Hypothetical protein |
| | Conserved hypothetical protein |
| | Cytochrome p450, putative |
| | Sialin, putative |
| | Sialin, putative |
| | Ubiquitin-protein ligase, putative |
| | Minor histocompatibility antigen H13, putative |
| | Receptor protein kinase, putative |
| | Pentatricopeptide repeat-containing protein, putative |
| | Receptor protein kinase, putative |
| | Receptor protein kinase, putative |
| | Conserved hypothetical protein |
| | Conserved hypothetical protein |
| | Conserved hypothetical protein |
| | CRP, putative |
| | **mHbCIRA2730** |
| | Rop guanine nucleotide exchange factor, putative |
| | Pentatricopeptide repeat-containing protein, putative |
| | Pentatricopeptide repeat-containing protein, putative |
| | Gibberellin 20-oxidase, putative |
| | Transcription factor, putative |
| | Beta-amylase, putative |
| | Beta-amylase, putative |
| | Calmodulin-binding heat-shock protein, putative |
| | 60S ribosomal protein L36e, putative |
| | Acyl-CoA thioesterase, putative |
| | Protein AFR, putative |
| | Conserved hypothetical protein |
| | Hypothetical protein |
| | DMT1 gene for chloroplast-resident DNA methyltransferase |
| | Receptor protein kinase, putative |
| | DNA binding protein, putative |
| | Kiwellin, putative |
| | Kiwellin, putative |

| | |
|---|---|
| | Synaptotagmin, putative |
| | Actin depolymerizing factor, putative |
| | KDEL motif-containing protein 1 precursor, putative |
| | KDEL motif-containing protein 1 precursor, putative |
| | Double-stranded RNA-specific adenosine deaminase |
| | tRNA-specific adenosine deaminase 1-like |
| | Remorin, putative |
| | R2R3-MYB transcription factor, putative |
| | **gHbCIRA2741** |
| | Dead box ATP-dependent RNA helicase, putative |
| | Acetyl-coenzyme A carboxylase carboxyl transferase alpha, putative |
| | Conserved hypothetical protein |
| | **Linkage map-g11** |
| **Scaffold 492213** | |
| | **mHbCIRBAC83E19** |
| **Scaffold 201737** | |
| | Calmodulin binding protein, putative |
| | **mHbCIRTAs2260** |
| **Scaffold 280583** | |
| | **mHbCIRa268** |
| **Scaffold 158826** | |
| | **mHbCIRA2736** |
| | fkbp-rapamycin associated protein, putative |
| **Scaffold 45077** | |
| | ADP-ribosylation factor, putative |
| | **mHbCIRA2536** |
| | Transcription factor, putative |
| **Scaffold 25873** | |
| | **mHbCIRA297** |
| **Scaffold 438547** | |
| | **mHbCIRa2535** |
| | Glycoprotein 3-alpha-l-fucosyltransferase A, putative |
| | Molybdopterin-binding, putative |
| | Molybdopterin-binding, putative |
| | **Linkage map-g12** |
| **Scaffold 130216** | |
| | Hedgehog receptor, putative |
| | **mHbCIRA2484** |
| **Scaffold 451604** | |
| | SAB, putative |
| | SAB, putative |
| | SAB, putative |
| | SAB, putative |
| | SAB, putative |
| | GATA transcription factor, putative |

| | |
|---|---|
| | GATA transcription factor, putative |
| | UDP-glucosyltransferase, putative |
| | UDP-glucosyltransferase, putative |
| | Conserved hypothetical protein |
| | Conserved hypothetical protein |
| | Conserved hypothetical protein |
| | Conserved hypothetical protein |
| | Conserved hypothetical protein |
| | map3k delta-1 protein kinase, putative |
| | Predicted protein |
| | Conserved hypothetical protein |
| | Protein VERNALIZATION-INSENSITIVE, putative |
| | Protein VERNALIZATION-INSENSITIVE, putative |
| | Cytochrome P450, putative |
| | Cytochrome P450, putative |
| | DNA binding protein, putative |
| | DNA binding protein, putative |
| | Argininosuccinate lyase, putative |
| | Argininosuccinate lyase, putative |
| | **mHbCIRT2085** |
| | Conserved hypothetical protein |
| | Conserved hypothetical protein |
| | Conserved hypothetical protein |
| | Conserved hypothetical protein |
| **Scaffold 114620** | |
| | **mHbCIRTAs2698** |
| | Mitogen-activated protein kinase kinase kinase |
| **Scaffold 224948** | |
| | Hydrolase, putative |
| | Disease resistance protein RPP13, putative |
| | **mHbCIRT2588** |
| | Serine/threonine-protein kinase cx32, putative |
| | Dead box ATP-dependent RNA helicase, putative |
| | SAUR family protein (SAUR28) |
| **Scaffold 185123** | |
| | **mHbCIRA2715** |
| **Scaffold 193057** | |
| | Pentatricopeptide repeat-containing protein, putative |
| | Protein phosphatase methylesterase, putative |
| | Protein phosphatase methylesterase, putative |
| | Phosphoprotein phosphatase |
| | Cytochrome P450, putative |
| | Cytochrome P450, putative |
| | Tubulin-specific chaperone E, putative |
| | Tubulin-specific chaperone E, putative |
| | Conserved hypothetical protein |

| | |
|---|---|
| | Conserved hypothetical protein |
| | Conserved hypothetical protein |
| | Conserved hypothetical protein |
| | Conserved hypothetical protein |
| | **mHbCIRA2405** |
| | Ubiquitin ligase protein, lrsam1, putative |
| | Hypothetical protein |
| | Chlorophyll A/B binding protein, putative |
| | **Linkage map-g13** |
| **Scaffold 323928** | |
| | Conserved hypothetical protein |
| | Serine-threonine protein kinase, plant-type, putative |
| | DNA-binding protein MNB1B, putative |
| | Hypothetical protein |
| | DNA-binding protein MNB1B, putative |
| | Conserved hypothetical protein |
| | Predicted protein |
| | DNA repair helicase rad5,16, putative |
| | Aspartic proteinase nepenthesin-1 precursor, putative |
| | Aspartic proteinase nepenthesin-1 precursor, putative |
| | Protein arginine n-methyltransferase, putative |
| | Protein arginine n-methyltransferase, putative |
| | Mitochondrial import receptor subunit tom40, putative |
| | Mitochondrial import receptor subunit tom40, putative |
| | Reticulocalbin-2 precursor, putative |
| | **mHbCIRa140** |
| | Reticulocalbin-2 precursor, putative |
| | DNA binding protein, putative |
| | DNA binding protein, putative |
| | Conserved hypothetical protein |
| | Conserved hypothetical protein |
| | Glucose regulated repressor protein, putative |
| | Protein-s isoprenylcysteine O-methyltransferase, putative |
| | Conserved hypothetical protein |
| | Transmembrane protein 53-like |
| | Glycerol-3-phosphate dehydrogenase, putative |
| | Cytokinin dehydrogenase, putative |
| | Cytokinin dehydrogenase, putative |
| | Silene conica chromosome 76 mitochondrion sequence |
| | Glycine max protein IQ-DOMAIN 14-like |
| | N-acetyltransferase, putative |
| | Conserved hypothetical protein |
| | Hypothetical protein |
| | Hypothetical protein |
| | Nucleotide pyrophosphatase/phosphodiesterase, putative |
| | Transcription factor, putative |

| | |
|---|---|
| | Predicted protein |
| | Disease resistance response protein, putative |
| | Conserved hypothetical protein |
| | Conserved hypothetical protein |
| | Conserved hypothetical protein |
| | Conserved hypothetical protein |
| | Short chain alcohol dehydrogenase, putative |
| | Predicted protein |
| | O-methyltransferase, putative |
| | CC-NBS-LRR resistance protein |
| **Scaffold 123832** | |
| | Conserved hypothetical protein |
| | Nucleoporin NUP53, putative |
| | Hypothetical protein |
| | Conserved hypothetical protein |
| | Cell division cycle protein 27 homolog B-like |
| | Predicted protein |
| | DNA binding protein, putative |
| | DNA binding protein, putative |
| | **mHbCIRA2394** |
| | Conserved hypothetical protein |
| | Hypothetical protein |
| | Casein kinase, putative |
| | Casein kinase, putative |
| | Conserved hypothetical protein |
| | Conserved hypothetical protein |
| **Scaffold 207081** | |
| | Katanin p60 ATPase-containing subunit, putative |
| | **mHbCIRA2415** |
| | Katanin p60 ATPase-containing subunit, putative |
| **Scaffold 411742** | |
| | **mHbCIRA2523** |
| | Hypothetical protein |
| **Scaffold 411971** | |
| | Ethylene-insensitive3 protein, putative |
| | **mHbCIRA2508** |
| | **hmct21** |
| **Scaffold 411167** | |
| | Phenylalanine ammonia-lyase, putative |
| | Hypothetical protein |
| | Carbonic anhydrase, putative |
| | Conserved hypothetical protein |
| | **mHbCIRA2757** |
| | Bel1 homeotic protein, putative |
| | Conserved hypothetical protein |

| | |
|---|---|
| **Scaffold 148311** | |
| | **mHbCIRTAs2456** |
| | **Linkage map-g14** |
| **Scaffold 416127** | |
| | Predicted protein |
| | **mHbCIRa282** |
| **Scaffold 501411** | |
| | **mHbCIRA2435** |
| **Scaffold 156035** | |
| | Conserved hypothetical protein |
| | Hypothetical protein |
| | Protein binding protein, putative |
| | Conserved hypothetical protein |
| | Glycine-rich RNA-binding protein, putative |
| | Hypothetical protein |
| | Hypothetical protein |
| | Phosphoserine phosphatase, putative |
| | ATP synthase f1, gamma subunit, putative |
| | Mitochondrial carrier protein, putative |
| | Hypothetical protein |
| | Histone deacetylase hda1, putative |
| | **mHbCIRA2545** |
| | **hmct21** |
| | Hypothetical protein |
| | Hydrolase, hydrolyzing O-glycosyl compounds, putative |
| **Scaffold 126947** | |
| | Alpha-n-acetylglucosaminidase, putative |
| | Alpha-n-acetylglucosaminidase, putative |
| | Betaine-aldehyde dehydrogenase |
| | Betaine-aldehyde dehydrogenase |
| | Mannose-1-phosphate guanyltransferase, putative |
| | Pentatricopeptide repeat-containing protein, putative |
| | Cell division cycle, putative |
| | Two-component sensor protein histidine protein kinase, putative |
| | Actin binding protein, putative |
| | Lipase, putative |
| | Conserved hypothetical protein |
| | Microtubule-associated protein TORTIFOLIA1, putative |
| | Conserved hypothetical protein |
| | DNA binding protein, putative |
| | *Hevea brasiliensis* isolate SSH16 |
| | Conserved hypothetical protein |
| | **mHbCIRA2423** |
| | Predicted protein |
| | Hypothetical protein |
| | Hypothetical protein |

| | |
|---|---|
| | Hypothetical protein |
| | Predicted protein |
| | 5-formyltetrahydrofolate cyclo-ligase, putative |
| | 5-formyltetrahydrofolate cyclo-ligase, putative |
| | Rhicadhesin receptor precursor, putative |
| | DNA binding protein, putative |
| | **GTR20** |
| | Conserved hypothetical protein |
| | R2R3-MYB transcription factor, putative |
| | Galactosyltransferase, putative |
| | Predicted protein |
| | Predicted protein |
| | ATP binding protein, putative |
| | ATP binding protein, putative |
| | ATP binding protein, putative |
| | tRNA ligase, putative |
| | tRNA ligase, putative |
| | tRNA ligase, putative |
| | HIPL1 protein precursor, putative |
| | Epstein-Barr nuclear antigen, putative |
| | Predicted protein |
| | Conserved hypothetical protein |
| | Conserved hypothetical protein |
| | LOB domain-containing protein, putative |
| | Conserved hypothetical protein |
| | **ACR24** |
| **Scaffold 409802** | |
| | Brassinosteroid insensitive 1-associated receptor kinase 1 precursor |
| | Protein bem46, putative |
| | Protein tipD, putative |
| | Conserved hypothetical protein |
| | Cysteine protease, putative |
| | Hypothetical protein |
| | Predicted protein |
| | Conserved hypothetical protein |
| | Predicted protein |
| | Nucleic acid binding protein, putative |
| | Nucleic acid binding protein, putative |
| | Nucleic acid binding protein, putative |
| | Predicted protein |
| | Transferase, transferring glycosyl groups, putative |
| | Transferase, transferring glycosyl groups, putative |
| | Esterase precursor, putative |
| | Ubiquitin-protein ligase, putative |
| | Elongator complex protein, putative |
| | Predicted protein |

| | |
|---|---|
| | Sentrin/sumo-specific protease, putative |
| | Predicted protein |
| | Ubiquinone biosynthesis protein coq-8, putative |
| | Hypothetical protein |
| | Nucleoside diphosphate kinase, putative |
| | DELLA protein RGL1, putative |
| | Anthranilate N-benzoyltransferase protein, putative |
| | Anthranilate N-benzoyltransferase protein, putative |
| | Conserved hypothetical protein |
| | Glyceraldehyde 3-phosphate dehydrogenase, putative |
| | Conserved hypothetical protein |
| | Conserved hypothetical protein |
| | Predicted protein |
| | Bromodomain-containing protein, putative |
| | Bromodomain-containing protein, putative |
| | Predicted protein |
| | Conserved hypothetical protein |
| | **mHbCIRA2504** |
| | AMP dependent CoA ligase, putative |
| **Scaffold 120605** | |
| | **mHbCIRTA2452** |
| | Conserved hypothetical protein |
| | **Linkage map-g15** |
| **Scaffold 169399** | |
| | Hypothetical protein |
| | Sodium-bile acid cotransporter, putative |
| | **mHbCIRa289** |
| | Bromodomain-containing protein, putative |
| | **mHbCIRA2391** |
| | Predicted protein |
| | Bromodomain-containing protein, putative |
| | Hypothetical protein |
| | Transmembrane receptor, putative |
| | Hypothetical protein |
| | Hypothetical protein |
| **Scaffold 134709** | |
| | Nucleic acid binding protein, putative |
| | Nucleic acid binding protein, putative |
| | Protein kinase APK1B, chloroplast precursor, putative |
| | Predicted protein |
| | **mHbCIRT2086** |
| | Hypothetical protein |
| **Scaffold 435366** | |
| | Conserved hypothetical protein |
| | Protein binding protein, putative |
| | Protein binding protein, putative |

| | |
|---|---|
| | Protein binding protein, putative |
| | Serine carboxypeptidase, putative |
| | **mHbCIRA2483** |
| | **mHbCIRA2391** |
| | **mHbCIRA314** |
| | Sterol regulatory element-binding protein site 2 protease, putative |
| **Scaffold 129826** | |
| | **mHbCIRTAs2709** |
| **Scaffold 43997** | |
| | NAD dehydrogenase, putative |
| | NAD dehydrogenase, putative |
| | Conserved hypothetical protein |
| | Predicted protein |
| | **mHbCIRA2391** |
| | Predicted protein |
| | Dihydrolipoamide acetyltransferase component of pyruvate dehydrogenase, putative |
| | **mHbCIRA2510** |
| | Dihydrolipoamide acetyltransferase component of pyruvate dehydrogenase, putative |
| | MTA/SAH nucleosidase, putative |
| | **hmct53** |
| | **Hbtnr-71** |
| | **mHbCIRt182** |
| | **gHbCIRA2685** |
| | 3-Phosphoinositide-dependent protein kinase-1, putative |
| | 3-Phosphoinositide-dependent protein kinase-1, putative |
| **Scaffold 42633** | |
| | Conserved hypothetical protein |
| | Lipid binding protein, putative |
| | Conserved hypothetical protein |
| | Predicted protein |
| | **mHbCIRA2697** |
| | **mHbCIRa390** |
| | **hmct21** |
| | **hmct22** |
| | Serine-threonine protein kinase, plant-type, putative |
| **Scaffold 254588** | |
| | Hypothetical protein |
| | Pentatricopeptide repeat-containing protein, putative |
| | Two-component system sensor histidine kinase/response regulator, putative |
| | Hypothetical protein |
| | Leucine-rich repeat-containing protein 2, lrrc2, putative |
| | Leucine-rich repeat-containing protein 2, lrrc2, putative |
| | Leucine-rich repeat-containing protein 2, lrrc2, putative |
| | ATP binding protein, putative |
| | Conserved hypothetical protein |
| | Conserved hypothetical protein |

| | |
|---|---|
| | Conserved hypothetical protein |
| | RING-H2 finger protein ATL3C, putative |
| | Transcription factor RF2b, putative |
| | Dihydropteroate synthase, putative |
| | Predicted protein |
| | Predicted protein |
| | **mHbCIRA2391** |
| | Hypothetical protein |
| | **mHbCIRTA2157** |
| | **hmct40** |
| | Hypothetical protein |
| | Hypothetical protein |
| | **mHbCIRa095** |
| | ATP binding protein, putative |
| | Mn-superoxide dismutase (SODMn) gene |
| | Conserved hypothetical protein |
| | Predicted protein |
| | Aspartic proteinase nepenthesin-1 precursor, putative |
| **Scaffold 119584** | |
| | Dead box ATP-dependent RNA helicase, putative |
| | Hypothetical protein |
| | Hypothetical protein |
| | **mHbCIRA2492** |
| | Predicted protein |
| | **mHbCIRA2492** |
| | **mHbCIRTA2157** |
| | **Linkage map-g16** |
| **Scaffold 154109** | |
| | Signal recognition particle receptor subunit beta, putative |
| | **mHbCIRTAs2225** |
| **Scaffold 134200** | |
| | **hmct40** |
| | Paramyosin, putative |
| | Cytochrome P450, putative |
| | Afc, putative |
| | Pentatricopeptide repeat-containing protein, putative |
| | Alpha-galactosidase/alpha-n-acetylgalactosaminidase, putative |
| | **mHbCIRA131** |
| | Alpha-L-fucosidase 2 precursor, putative |
| | Alpha-L-fucosidase 2 precursor, putative |
| | Protein phosphatase 2c, putative |
| | ATP binding protein, putative |
| | Conserved hypothetical protein |
| | Protein phosphatase 2c, putative |
| | Cytochrome P450, putative |

| | |
|---|---|
| | Serine/threonine-protein kinase bri1, putative |
| | ATP binding protein, putative |
| | Suppressor of ty, putative |
| | Predicted protein |
| | Cysteine desulfuration protein sufe, putative |
| | Cytochrome P450, putative |
| | Hypothetical protein |
| **Scaffold 251160** | |
| | Axial regulator YABBY4, putative |
| | **mHbCIRA2407** |
| **Scaffold 177599** | |
| | Hypothetical protein |
| | Heat shock protein 70 (HSP70)-interacting protein, putative |
| | Predicted protein |
| | Alcohol dehydrogenase, putative |
| | **mHbCIRA097** |
| | Nucleotide binding protein, putative |
| | Predicted protein |
| **Scaffold 158956** | |
| | **mHbCIRA2765** |
| | Ribonuclease z, chloroplast, putative |
| | Ribonuclease z, chloroplast, putative |
| **Scaffold 307979** | |
| | **Hbtnr-17** |
| | **mHbCIRA275** |
| | Beta-1,3-galactosyltransferase sqv-2, putative |
| **Scaffold 420218** | |
| | MADs box protein, putative |
| | **mHbCIRA2396** |
| **Scaffold 234640** | |
| | **mHbCIRA179** |
| | **Linkage map-g17** |
| **Scaffold 121986** | |
| | **mHbCIRTs2188** |
| **Scaffold 413909** | |
| | **gHbCIRTAs2720** |
| | Conserved hypothetical protein |
| | Conserved hypothetical protein |
| | Conserved hypothetical protein |
| | Glucan endo-1,3-beta-glucosidase precursor, putative |
| | **mHbCIRA2414** |
| | Hypothetical protein |
| | Conserved hypothetical protein |
| | Conserved hypothetical protein |
| | DUF26 domain-containing protein 1 precursor, putative |
| | Conserved hypothetical protein |

| | |
|---|---|
| | Nucleic acid binding protein, putative |
| | Nucleic acid binding protein, putative |
| | Calcium ion binding protein, putative |
| | Ubiquitin-conjugating enzyme, putative |
| | **hmac2** |
| | **hmct21** |
| | **gHbCIRA2511** |
| **Scaffold 418287** | |
| | **hmct12** |
| | **mHbCIRA2425** |
| | Beta-ketoacyl-ACP synthase I |
| | **mHbCIRa344** |
| | **mHbCIRa78** |
| | Conserved hypothetical protein |
| | Urease accessory protein ureG, putative |
| | DNA replication licensing factor MCM3, putative |
| | Beta-ketoacyl-ACP synthase I |
| **Scaffold 433878** | |
| | Pectinesterase (MTR_3g010770) |
| | Coatomer gamma subunit, putative |
| | **mHbCIRT140** |
| | Guanine nucleotide-binding protein beta, putative |
| | Glucan endo-1,3-beta-glucosidase precursor, putative |
| | **mHbCIRA2418-2** |
| | Adenylyl cyclase-associated protein, putative |
| | Cytochrome P450 3A27 putative |
| | Serine/threonine-protein kinase PBS1, putative |
| | Hypothetical protein |
| | Predicted protein |
| | 2-Hydroxyacid dehydrogenase, putative |
| | Conserved hypothetical protein |
| | Predicted protein |
| | NADH-ubiquinone oxidoreductase 1, chain, putative |
| | Phenylalanyl-trna synthetase beta chain, putative |
| | Phenylalanyl-trna synthetase beta chain, putative |
| | Conserved hypothetical protein |
| | lrr receptor protein kinase, putative |
| | Hypothetical protein |
| | DNA binding protein, putative |
| | Hypothetical protein |
| | Hypothetical protein |
| | Predicted protein |
| | Predicted protein |
| | Acyltransferase, putative |
| | Hypothetical protein |

| | |
|---|---|
| **Scaffold 133200** | |
| | **mHbCIRT2447** |
| **Scaffold 521115** | |
| | **mHbCIRA2391** |
| | Oligopeptide transporter, putative |
| | Cadmium-induced protein AS8, putative |
| | **mHbCIRA2387** |
| | Predicted protein |
| | **mHbCIRTA2157** |
| | **hmac3** |
| | **Linkage map-g18** |
| **Scaffold 122775** | |
| | Multidrug resistance protein ABC transporter family |
| | Multidrug resistance protein ABC transporter family |
| | Mitochondrial translational initiation factor, putative |
| | Ubiquitin ligase protein cop1, putative |
| | Ubiquitin ligase protein cop1, putative |
| | Predicted protein |
| | Predicted protein |
| | Hypothetical protein |
| | Ubiquitin-conjugating enzyme E2, putative |
| | Predicted protein |
| | Aldose 1-epimerase, putative |
| | Beta-1,3-galactosyltransferase sqv-2, putative |
| | 50S ribosomal protein L18, chloroplast precursor, putative |
| | **mHbCIRT2113** |
| | Predicted protein |
| **Scaffold 174767** | |
| | **mHbCIRA2197** |
| **Scaffold 108198** | |
| | Glutathione s-transferase, putative |
| | Conserved hypothetical protein |
| | Conserved hypothetical protein |
| | Conserved hypothetical protein |
| | Conserved hypothetical protein |
| | DNA binding protein, putative |
| | UTP-glucose-1-phosphate uridylyltransferase, putative |
| | UTP-glucose-1-phosphate uridylyltransferase, putative |
| | NADP-specific isocitrate dehydrogenase, putative |
| | Glutamate decarboxylase, putative |
| | Conserved hypothetical protein |
| | nnp-1 protein, putative |
| | Predicted protein |
| | Conserved hypothetical protein |
| | Conserved hypothetical protein |

| | |
|---|---|
| | Conserved hypothetical protein |
| | Transcription factor, putative |
| | Predicted protein |
| | Nonsense-mediated decay protein, putative |
| | **mHbCIRA2422** |
| | Importin beta-3, putative |
| | Oxidoreductase, putative |
| | Conserved hypothetical protein |
| | Zinc finger protein, putative |
| | Receptor protein kinase, putative |
| **Scaffold 155107** | |
| | Amine oxidase, putative |
| | O-linked n-acetylglucosamine transferase, ogt, putative |
| | O-linked n-acetylglucosamine transferase, ogt, putative |
| | N-acetylglucosamine kinase, putative |
| | N-acetylglucosamine kinase, putative |
| | **mHbCIRA463** |
| | Conserved hypothetical protein |
| | Serine/threonine-protein kinase PBS1, putative |
| | Serine/threonine-protein kinase PBS1, putative |
| | Small nucleolar RNA-associated protein 14 homolog A-like protein kinase, putative |
| | F-box/leucine rich repeat protein, putative |
| | F-box/leucine rich repeat protein, putative |
| | Transcription initiation factor TFIID subunit |
| | Conserved hypothetical protein |
| | Hypothetical protein |
| | Phosphoenolpyruvate carboxylase |
| **Scaffold 181819** | |
| | **mHbCIRA320** |
| **Scaffold 436803** | |
| | Protein with unknown function |
| | Transcription factor, putative |
| | Serine/threonine-protein kinase cx32, putative |
| | Serine/threonine-protein kinase cx32, putative |
| | Dihydrodipicolinate synthase, putative |
| | Nucellin, putative |
| | Disease resistance response protein, putative |
| | Suppressor of actin, putative |
| | **mHbCIRA2482** |
| | Catalase (cat) |
| | Hypothetical protein |
| | Plant ubiquilin, putative |
| | **SSR P2-D6-2** |
| | Cyclin B, putative |
| | Predicted protein |

| | |
|---|---|
| | GTP binding protein, putative |
| | GTP binding protein, putative |
| | Predicted protein |
| | Hypothetical protein |
| | Isocitrate dehydrogenase, putative |
| **Scaffold 155502** | |
| | Hypothetical protein |
| | **mHbCIRA2409** |
| **Scaffold 577425** | |
| | Sodium-dependent phosphate transport protein, putative |
| | **mHbCIRTAs2217** |
| | Cytochrome P450, putative |
| | Conserved hypothetical protein |
| | Predicted protein |
| | Josephin-2, putative |
| | Predicted protein |
| | Catalytic, putative |
| | Catalytic, putative |
| | Predicted protein |
| | Conserved hypothetical protein |
| | Conserved hypothetical protein |
| | Sugar transporter, putative |
| | Predicted protein |
| | Predicted protein |
| | DNA binding protein, putative |
| | phosphatidylinositol n-acetylglucosaminyltransferase subunit p, putative |
| | conserved hypothetical protein |
| | **mHbCIRA2391** |
| **Scaffold 410482** | |
| | **mHbCIRT373** |
| | Hypothetical protein |
| | **mHbCIRT2391** |
| | Serine-threonine protein kinase, plant-type, putative |
| | Serine-threonine protein kinase, plant-type, putative |
| | Serine-threonine protein kinase, plant-type, putative |
| | ATP binding protein, putative |
| **Scaffold 513701** | |
| | DNA mismatch repair protein mlh1, putative |
| | DNA mismatch repair protein mlh1, putative |
| | DNA mismatch repair protein mlh1, putative |
| | Dead box ATP-dependent RNA helicase, putative |
| | **ACR37** |
| | **mHbCIRT1067** |
| | Hydrolase, putative |
| | Hypothetical protein |
| | Prefoldin-related ke2-like protein |

| | |
|---|---|
| **Scaffold 128818** | |
| | Conserved hypothetical protein |
| | **mHbCIRA2439** |
| | Conserved hypothetical protein |
| | Conserved hypothetical protein |
| | Conserved hypothetical protein |
| | Serine-threonine protein kinase, plant-type, putative |
| **Scaffold 467142** | |
| | **mHbCIRTAs 2744** |
| | Aspartate kinase, putative |
| **Scaffold 336055** | |
| | **mHbCIRA288** |
| **Scaffold 308928** | |
| | **mHbCIRTAs 2271** |
| | Sterol desaturase, putative |

**Table S3.** Main classes of repeat elements in the *H. brasiliensis* genome assembly

| **Class** | **Types** | **Total repeats (%)** |
|---|---|---|
| **DNA transposons** | En-Spm | 0.61 |
| | MuDR | 0.45 |
| | hAT-Tag1 | 0.21 |
| | hAT-Ac | 0.14 |
| | hAT-Tip100 | 0.09 |
| | Helitron | 0.01 |
| | Others | 0.05 |
| **Retrotransposons** | LTR/Gypsy | 38.20 |
| | LTR/Copia | 7.38 |
| | LTR/Caulimovirus | 0.39 |
| | LINE/ RTE-BovB | 0.97 |
| | LINE/ L1 | 0.34 |
| | LINE/CR1 | 0.05 |
| | LINE/ R1 | 0.02 |
| | SINE | 0.01 |
| | Others | 0.18 |
| **Satellite** | | 0.02 |
| **Simple repeat** | | 0.64 |
| **Unknown** | | 50.24 |

**Table S4.** Summary statistics of gene models predicted by seven programs

| | **EVM** | **AUGUSTUS** | **FGENESH** | **GENEID** | **GENEMARK HMME** | **GLIMMER HMM** | **SNAP** |
|---|---|---|---|---|---|---|---|
| Number of models | 68,955 | 48,087 | 62,020 | 65,851 | 65,669 | 65,902 | 59,591 |
| Total number of bases | 47,978,108 | 41,396,159 | 47,897,431 | 39,074,868 | 44,307,210 | 30,116,191 | 41,503,482 |
| Average ORF length (bp) | 696 | 861 | 772 | 593 | 675 | 457 | 696 |

**Table S5.** Comparison of publicly available *H. brasiliensis* transcripts with the genome

| Resource | Accession No. | *Hevea* clone | No. Sequences | No. Aligned[a] | % Aligned |
|---|---|---|---|---|---|
| NCBI dbEST[b] | Various | Primarily (>90%) RRIM600 | 37,826 | 36,786 | 97.25 |
| BIOTEC, Thailand[c] | N/A | RRIM600 | 28,300 | 28,237 | 99.78 |
| NCBI GEO[d] | GSE26514 | RY7-33-97 | 48,768 | 45,640 | 93.59 |
| NCBI TSA[e] | JR344291-JR366936 | RY7-33-97 | 22,646 | 20,589 | 90.92 |
| **Total** | | | **137,540** | **131,252** | **95.43** |

[a]BLAST hits with E-value < $10^{-5}$ and 90% sequence identity cutoff
[b]Data available as of October 2012
[c]Isotig sequences from http://www4a.biotec.or.th/rubber/Download (ref. 6)
[d]Unigenes from ref. 7
[e]Assembled transcripts from ref. 10

**Table S6.** General features of the transcriptome assembly

| | |
|---|---|
| Number of contigs | 73,060 |
| Mean length of contigs (bp) | 1,173 |
| Length of the largest contig (bp) | 15,597 |
| Number of contigs aligned to the genome (>90% sequence identity) | 72,644 |
| Number of contigs with NR alignment | 55,154 |
| Number of unique GI accession IDs | 19,788 |
| Number of contigs with matching protein coverage of 80-100% | 18,571 |
| Number of unique GIs with matching protein coverage of 80-100% | 10,161 |
| Number of contigs with matching protein coverage of 50-80% | 13,198 |
| Number of unique GIs with matching protein coverage of 50-80% | 6,593 |

**Table S7.** Functional annotation of predicted proteins for *H. brasiliensis*

| Protein databases | Number of proteins |
|---|---|
| NR alignment | 52,968 |
| KEGG alignment | 52,825 |
| KOG/COG assignment | 33,472 |
| EC assignment | 16,779 |
| KO assignment | 28,488 |
| GO assignment | 26,816 |
| InterPro signature | 41,743 |

**Table S8.** Comparison of KOG functions across various sequenced plant genomes

| KOG Functions | *Hevea brasiliensis* | *Ricinus communis* | *Populus trichocarpa* | *Arabidopsis thaliana* | *Eucalyptus grandis* | *Oryza sativa* | *Zea mays* |
|---|---|---|---|---|---|---|---|
| **INFORMATION STORAGE AND PROCESSING** | | | | | | | |
| [J] Translation, ribosomal structure and biogenesis | 1236 | 663 | 1129 | 1068 | 1207 | 1109 | 2290 |
| [A] RNA processing and modification | 1067 | 547 | 999 | 940 | 1023 | 976 | 1814 |
| [K] Transcription | 1459 | 826 | 1619 | 1618 | 1608 | 1508 | 2419 |
| [L] Replication, recombination and repair | 661 | 314 | 654 | 504 | 595 | 486 | 853 |
| [B] Chromatin structure and dynamics | 377 | 183 | 348 | 349 | 323 | 284 | 578 |
| **CELLULAR PROCESSES AND SIGNALING** | | | | | | | |
| [D] Cell cycle control, cell division, chromosome partitioning | 627 | 300 | 537 | 565 | 593 | 495 | 969 |
| [Y] Nuclear structure | 122 | 40 | 80 | 72 | 60 | 62 | 128 |
| [V] Defense mechanisms | 274 | 136 | 261 | 180 | 334 | 280 | 370 |
| [T] Signal transduction mechanisms | 5216 | 1959 | 4288 | 2837 | 6806 | 4006 | 4857 |
| [M] Cell wall/membrane/envelope biogenesis | 464 | 202 | 366 | 288 | 478 | 365 | 589 |
| [N] Cell motility | 7 | 2 | 3 | 5 | 4 | 6 | 2 |
| [Z] Cytoskeleton | 1092 | 404 | 826 | 701 | 1046 | 697 | 1333 |
| [W] Extracellular structures | 116 | 55 | 115 | 66 | 81 | 73 | 91 |
| [U] Intracellular trafficking, secretion, and vesicular transport | 1496 | 638 | 1276 | 1142 | 1367 | 1188 | 2210 |
| [O] Posttranslational modification, protein turnover, chaperones | 2886 | 1486 | 2691 | 2396 | 3227 | 2726 | 4451 |
| **METABOLISM** | | | | | | | |
| [C] Energy production and conversion | 1119 | 657 | 973 | 749 | 1555 | 982 | 1585 |
| [G] Carbohydrate transport and metabolism | 1665 | 804 | 1354 | 1102 | 1817 | 1336 | 1993 |
| [E] Amino acid transport and metabolism | 1119 | 613 | 962 | 809 | 1266 | 1011 | 1565 |
| [F] Nucleotide transport and metabolism | 299 | 157 | 232 | 215 | 243 | 281 | 475 |
| [H] Coenzyme transport and metabolism | 289 | 159 | 239 | 212 | 292 | 235 | 393 |
| [I] Lipid transport and metabolism | 1178 | 638 | 1038 | 832 | 1478 | 963 | 1635 |
| [P] Inorganic ion transport and metabolism | 902 | 433 | 848 | 590 | 963 | 673 | 1045 |
| [Q] Secondary metabolites biosynthesis, transport and catabolism | 1470 | 768 | 1171 | 785 | 2007 | 1018 | 1289 |
| **POORLY CHARACTERIZED** | | | | | | | |
| [R] General function prediction only | 3493 | 1834 | 3085 | 2790 | 4156 | 3023 | 4499 |
| [S] Function unknown | 1449 | 723 | 1388 | 1163 | 1675 | 1282 | 2240 |

**Table S9.** Pfam domains in the *H. brasiliensis* genome. Only the most abundant domains (≥10 hits) are listed.

| Pfam Domain | Hits |
|---|---|
| Leucine Rich Repeat | 7259 |
| Ankyrin repeat | 1030 |
| EF hand | 657 |
| GTPase of unknown function | 534 |
| ABC transporter | 518 |
| ATPase family associated with various cellular activities (AAA) | 433 |
| Binding-protein-dependent transport system inner membrane component | 348 |
| ATP synthase A chain | 319 |
| Cytochrome P450 | 292 |
| 7 transmembrane receptor (rhodopsin family) | 290 |
| Cytochrome C and Quinol oxidase polypeptide I | 283 |
| DEAD/DEAH box helicase | 211 |
| Elongation factor Tu GTP binding domain | 188 |
| Helicase conserved C-terminal domain | 147 |
| Homeobox domain | 134 |
| FAD dependent oxidoreductase | 129 |
| alpha/beta hydrolase fold | 128 |
| haloacid dehalogenase-like hydrolase | 114 |
| Elongation factor Tu domain 2 | 103 |
| Glycosyl transferases group 1 | 93 |
| CBS domain pair | 82 |
| His Kinase A (phosphoacceptor) domain | 80 |
| 4Fe-4S binding domain | 78 |
| Aminotransferase class I and II | 78 |
| Calcineurin-like phosphoesterase | 74 |
| ATP synthase alpha/beta family, nucleotide-binding domain | 69 |
| Alcohol dehydrogenase GroES-like domain | 63 |
| Histidine kinase-, DNA gyrase B-, and HSP90-like ATPase | 55 |
| Acetyltransferase (GNAT) family | 52 |
| AMP-binding enzyme | 52 |
| Cytochrome b(C-terminal)/b6/petD | 52 |
| 4Fe-4S iron sulfur cluster binding proteins, NifH/frxC family | 41 |
| Aldehyde dehydrogenase family | 41 |
| Glycosyl transferase family 2 | 39 |
| Cytochrome b(N-terminal)/b6/petB | 32 |
| Cytochrome C oxidase subunit II, transmembrane domain | 32 |
| HAMP domain | 30 |
| Elongation factor Tu C-terminal domain | 27 |
| Bacterial regulatory helix-turn-helix protein, lysR family | 24 |
| ATP synthase alpha/beta family, beta-barrel domain | 23 |
| Bacterial regulatory helix-turn-helix proteins, AraC family | 21 |
| Bacterial regulatory proteins, gntR family | 18 |
| gag gene protein p24 (core nucleocapsid protein) | 18 |
| Helix-turn-helix | 18 |
| Bacterial regulatory proteins, tetR family | 15 |
| gag gene protein p17 (matrix protein) | 15 |

**Table S10.** Predicted subcellular localization of *H. brasiliensis* gene models based on SignalP 3.0 analysis

| Localization Site | Hits | GO Cellular Component ID |
|---|---|---|
| Chloroplast | 1699 | 0005576, 0009543, 0009507 |
| Extracellular | 1367 | 0005618 |
| Plasma Membrane | 554 | 0005886 |
| Vacuolar Membrane | 368 | 0005774(2) |
| Cytosol | 286 | 0005829 |
| Nuclear | 249 | 0005634 |
| Endoplasmic Reticulum | 56 | 0005783 |
| Mitochondria | 47 | 0005739 |
| Golgi Apparatus | 12 | 0005794(1) |
| Peroxisome | 5 | 0005777(2) |
| **Total** | **4643** | |

**Table S11.** tRNA types found in the *H. brasiliensis* genome

| tRNA type | Number | Number of Introns |
|---|---|---|
| Ala | 37 | 1 |
| Arg | 50 | |
| Asn | 24 | |
| Asp | 29 | |
| Cys | 17 | |
| Gln | 19 | |
| Glu | 33 | |
| Gly | 58 | |
| His | 16 | |
| Ile | 33 | |
| Leu | 59 | 1 |
| Lys | 40 | |
| Met | 47 | 11 |
| Phe | 27 | |
| Pro | 14 | |
| Ser | 62 | |
| Thr | 42 | |
| Trp | 13 | |
| Tyr | 21 | 17 |
| Val | 40 | |
| Pseudo | 32 | 3 |
| Sup | 12 | |
| Undet | 4 | |
| **Total** | **729** | **33** |

**Table S12.** Gene Ontology (GO) analysis of *Hevea* specific genes. Only the most abundant GO terms (≥10 hits) are listed.

| GO terms | Hits | Description |
|---|---|---|
| GO:0005524 | 746 | ATP binding |
| GO:0000166 | 409 | Nucleotide binding |
| GO:0004672 | 374 | Protein kinase activity |
| GO:0006468 | 374 | Protein phosphorylation |
| GO:0006915 | 335 | Apoptosis |
| GO:0006952 | 325 | Defense response |
| GO:0004674 | 294 | Protein serine/threonine kinase activity |
| GO:0003676 | 278 | Nucleic acid binding |
| GO:0016301 | 232 | Kinase activity |
| GO:0016740 | 225 | Transferase activity |
| GO:0003677 | 204 | DNA binding |
| GO:0016491 | 198 | Oxidoreductase activity |
| GO:0008270 | 189 | Zinc ion binding |
| GO:0055114 | 189 | Oxidation-reduction process |
| GO:0016020 | 185 | Membrane |
| GO:0016787 | 171 | Hydrolase activity |
| GO:0016021 | 150 | Integral to membrane |
| GO:0008152 | 130 | Metabolic process |
| GO:0007165 | 122 | Signal transduction |
| GO:0004888 | 111 | Transmembrane signaling receptor activity |
| GO:0031224 | 111 | Intrinsic to membrane |
| GO:0045087 | 111 | Innate immune response |
| GO:0032440 | 107 | 2-alkenal reductase[NAD(p)] activity |
| GO:0017111 | 103 | Nucleoside-triphosphatase activity |
| GO:0003824 | 101 | Catalytic activity |
| GO:0015074 | 101 | DNA integration |
| GO:0004721 | 92 | Phosphoprotein phosphatase activity |
| GO:0004872 | 86 | Receptor activity |
| GO:0005488 | 85 | Binding |
| GO:0003723 | 79 | RNA binding |
| GO:0005634 | 76 | Nucleus |
| GO:0003964 | 74 | RNA-directed DNA polymerase activity |
| GO:0006278 | 63 | RNA-dependent DNA replication |
| GO:0005529 | 51 | Sugar binding |
| GO:0045449 | 49 | Regulation of transcription, DNA-dependent |
| GO:0004715 | 48 | Non-membrane spanning protein tyrosine kinase |
| GO:0046872 | 48 | Metal ion binding |
| GO:0006508 | 47 | Proteolysis |
| GO:0005975 | 44 | Carbohydrate metabolic process |
| GO:0006355 | 37 | Regulation of transcription, DNA-dependent |
| GO:0004713 | 36 | Protein tyrosine kinase activity |
| GO:0016310 | 36 | Phosphorylation |
| GO:0004553 | 35 | Hydrolase activity, hydrolyzing O-glycosyl |
| GO:0004523 | 34 | Ribonuclease H activity |
| GO:0005506 | 34 | Iron ion binding |
| GO:0004709 | 33 | MAP kinase kinase kinase activity |
| GO:0005622 | 33 | Intracellular |
| GO:0048544 | 33 | Recognition of pollen |
| GO:0004497 | 30 | Monooxygenase activity |
| GO:0020037 | 29 | Heme binding |
| GO:0005509 | 27 | Calcium ion binding |
| GO:0009055 | 27 | Electron carrier activity |
| GO:0043169 | 26 | Cation binding |
| GO:0006350 | 25 | Transcription, DNA-dependent |
| GO:0016758 | 25 | Transferase activity, transferring hexosyl groups |
| GO:0016798 | 25 | Hydrolase activity, acting on glycosyl bonds |

| | | |
|---|---|---|
| GO:0046983 | 23 | Protein dimerization activity |
| GO:0016829 | 22 | Lyase activity |
| GO:0016757 | 21 | Transferase activity, transferring glycosyl groups |
| GO:0005737 | 20 | Cytoplasm |
| GO:0055085 | 20 | Transmembrane transport |
| GO:0000287 | 19 | Magnesium ion binding |
| GO:0000785 | 19 | Chromatin |
| GO:0003682 | 19 | Chromatin binding |
| GO:0004190 | 19 | Aspartic-type endopeptidase activity |
| GO:0006333 | 19 | Chromatin assembly or disassembly |
| GO:0004697 | 18 | Protein kinase C activity |
| GO:0008233 | 16 | Peptidase activity |
| GO:0006351 | 14 | Transcription, DNA-dependent |
| GO:0008353 | 14 | RNA polymerase II carboxy-terminal domain kinase activity |
| GO:0000160 | 13 | Two-component signal transduction system (phosphorelay) |
| GO:0047213 | 13 | Anthocyanidin 3-O-glucosyltransferase activity |
| GO:0004714 | 12 | Transmembrane receptor protein tyrosine kinase activity |
| GO:0005525 | 12 | GTP binding |
| GO:0008236 | 12 | Serine-type peptidase activity |
| GO:0003700 | 11 | Sequence-specific DNA binding transcription factor activity |
| GO:0004252 | 11 | Serine-type endopeptidase activity |
| GO:0004568 | 11 | Chitinase activity |
| GO:0006032 | 11 | Chitin catabolic process |
| GO:0016998 | 11 | Cell wall macromolecule catabolic process |
| GO:0050660 | 11 | Flavin adenine dinucleotide binding |
| GO:0006810 | 10 | Transport |
| GO:0008168 | 10 | Methyltransferase activity |
| GO:0032259 | 10 | Methylation |
| GO:0044237 | 10 | Cellular metabolic process |
| GO:0050662 | 10 | Coenzyme binding |

**Table S13.** InterPro domains within the *Hevea* specific lineage. Only the most abundant domains (≥10 hits) are listed.

| InterPro ID | Hits | Domain Description |
|---|---|---|
| IPR001611 | 846 | Leucine-rich repeat |
| IPR017442 | 178 | Serine/threonine-protein kinase-like domain |
| IPR002885 | 124 | Pentatricopeptide repeat |
| IPR001245 | 110 | Serine-threonine/tyrosine-protein kinase |
| IPR002182 | 110 | NB-ARC |
| IPR013210 | 86 | Leucine-rich repeat-containing N-terminal, type 2 |
| IPR002110 | 54 | Ankyrin repeat |
| IPR000157 | 49 | Toll-Interleukin receptor |
| IPR005162 | 49 | Retrotransposon gag protein |
| IPR001128 | 35 | Cytochrome P450 |
| IPR004158 | 30 | Protein of unknown function DUF247, plant |
| IPR001480 | 24 | Bulb-type lectin domain |
| IPR013242 | 23 | Retroviral aspartyl protease |
| IPR005508 | 22 | Protein of unknown function DUF313 |
| IPR002902 | 20 | Gnk2-homologous domain |
| IPR003340 | 20 | Transcriptional factor B3 |
| IPR004252 | 18 | Transposase, Ptta/En/Spm, plant |
| IPR003656 | 17 | Zinc finger, BED-type predicted |
| IPR000626 | 16 | Ubiquitin |
| IPR021820 | 16 | S-locus receptor kinase, C-terminal |
| IPR001810 | 15 | F-box domain, cyclin-like |
| IPR002213 | 15 | UDP-glucuronosyl/UDP-glucosyltransferase |
| IPR000858 | 13 | S-locus glycoprotein |
| IPR000504 | 12 | RNA recognition motif domain |
| IPR001878 | 12 | Zinc finger, CCHC-type |
| IPR003676 | 12 | Auxin responsive SAUR protein |
| IPR005630 | 12 | Terpene synthase, metal-binding domain |
| IPR013695 | 12 | Wall-associated kinase |
| IPR001153 | 11 | Barwin |
| IPR005123 | 11 | Oxoglutarate/iron-dependent oxygenase |
| IPR023210 | 11 | NADP-dependent oxidoreductase domain |
| IPR001360 | 10 | Glycoside hydrolase, family 1 |
| IPR011713 | 10 | Leucine-rich repeat 3 |

**Table S14.** Pfam domains within the *Hevea* specific lineage. Only the most abundant domains (≥10 hits) are listed.

| Pfam ID | Hits | Domain Description |
|---|---|---|
| PF00560 | 846 | LRR_1 |
| PF00069 | 178 | Pkinase |
| PF01535 | 124 | PR |
| PF00931 | 110 | NB-ARC |
| PF07714 | 110 | Pkinase_Tyr |
| PF08263 | 86 | LRRNT_2 |
| PF00023 | 54 | Ank |
| PF01582 | 49 | TIR |
| PF03732 | 49 | Retrotrans_gag |
| PF00067 | 35 | p450 |
| PF03140 | 30 | Domain of unknown function |
| PF01453 | 24 | D-mannose binding lectin |
| PF08284 | 23 | Retroviral aspartyl protease |
| PF03754 | 22 | Domain of unknown function (DUF313) |
| PF01657 | 20 | Domain of unknown function |
| PF02362 | 20 | B3 domain |
| PF03004 | 18 | Plant transposase (Ptta/En/Spm family) |
| PF02892 | 17 | ED zinc finger |
| PF00240 | 16 | Ubiquitin |
| PF11883 | 16 | Domain of unknown function |
| PF00201 | 15 | Glucuronosyltransferase |
| PF00646 | 15 | F-box protein |
| PF00954 | 13 | S-locus glycoprotein family |
| PF00076 | 12 | RNA recognition motif |
| PF00098 | 12 | Zinc finger |
| PF02519 | 12 | Auxin responsive protein |
| PF03936 | 12 | Terpene synthase family, metal binding domain |
| PF08488 | 12 | Wall-associated kinase |
| PF00248 | 11 | Aldo-keto reductase |
| PF00967 | 11 | Barwin domain |
| PF03171 | 11 | 2OG-Fe(II) oxygenase superfamily |
| PF00232 | 10 | Glycoside hydrolase family 1 |
| PF07725 | 10 | Leucine Rich Repeat |

**Table S15.** KOG analysis of *Hevea* specific genes

| KOG Functions | Counts |
|---|---|
| *Information and Storage and Processing* | |
| [J] Translation, ribosomal structure and biogenesis | 27 |
| [A] RNA processing and modification | 18 |
| [K] Transcription | 21 |
| [L] Replication, recombination and repair | 30 |
| [B] Chromatin structure and dynamics | 10 |
| *Cellular Processes and Signaling* | |
| [D] Cell cycle control, cell division, chromosome partitioning | 17 |
| [Y] Nuclear structure | 2 |
| [V] Defense mechanisms | 16 |
| [T] Signal transduction mechanisms | 603 |
| [M] Cell wall/membrane/envelope biogenesis | 29 |
| [N] Cell motility | 0 |
| [Z] Cytoskeleton | 116 |
| [W] Extracellular structures | 11 |
| [U] Intracellular trafficking, secretion, and vesicular transport | 13 |
| [O] Posttranslational modification, protein turnover, chaperones | 89 |
| *Metabolism* | |
| [C] Energy production and conversion | 48 |
| [G] Carbohydrate transport and metabolism | 74 |
| [E] Amino acid transport and metabolism | 38 |
| [F] Nucleotide transport and metabolism | 2 |
| [H] Coenzyme transport and metabolism | 12 |
| [I] Lipid transport and metabolism | 32 |
| [P] Inorganic ion transport and metabolism | 22 |
| [Q] Secondary metabolites biosynthesis, transport and catabolism | 80 |
| *Poorly Characterized* | |
| [R] General function prediction only | 140 |
| [S] Function unknown | 67 |

**Table S16.** Rubber biosynthesis related genes in the *H. brasiliensis* genome. Shading indicates the transcript has been validated from leaf and/or latex cDNA pools.

| Gene model | Gene name | EC No. | Members |
|---|---|---|---|
| HBM _contig317144 _s139324c15 _gene2 | 1,4-alpha-glucan branching enzyme | 2.4.1.18 | 1 |
| HBM_contig1307528_s417190c7_gene1<br>HBM_contig343183_s151438c5_gene1 | 1-deoxy-D-xylulose-5-phosphate isomeroreductase | 1.1.1.267 | 2 |
| HBM_contig1288739_s409811c4_gene1<br>HBM_contig529416_s246980c14_gene1<br>HBM_contig199115_s88445_gene1<br>HBM_contig366145_s162700_gene1<br>HBM_contig979245_s352662c2_gene3<br>HBM_contig1343951_s444526c6_gene1<br>HBM_contig1404580_s501548c30_gene1 | 1-deoxyxylulose-5-phosphate synthase | 2.2.1.7 | 7 |
| HBM_contig1289901_s410013c1_gene1<br>HBM_contig1297666_s412376_gene1 | 2,1-fructan:2,1-fructan 1-fructosyltransferase/levansucrase | 2.4.1.10 | 2 |
| HBM_contig1326688_s430296c2_gene2<br>HBM_contig217879_s96943c3_gene1 | 2-C-methyl-D-erythritol 2,4-cyclodiphosphate synthase | 4.6.1.12 | 2 |
| HBM_contig1339539_s440773c5_gene3<br>HBM_contig229209_s101968_gene1 | 2-C-methyl-D-erythritol 4-phosphate cytidylyltransferase | 2.7.7.60 | 2 |
| HBM _contig382299 _s170997c9 _gene2 | 2-oxoglutaratedehydrogenase | 1.2.4.2 | 1 |
| HBM _contig395100 _s177727c26 _gene1 | 4-alpha-glucanotransferase | 2.4.1.25 | 1 |
| HBM_contig1308223_s417615c21_gene1<br>HBM_contig302280_s132669c17_gene1 | 4-diphosphocytidyl-2-C-methyl-d-erythritol kinase | 2.7.1.148 | 2 |
| HBM_contig318055_s139710c8_gene1<br>HBM_contig276900_s122507c5_gene1<br>HBM_contig297371_s130496_gene1 | 4-hydroxy-3-methylbut-2-enyl diphosphate reductase | 1.17.1.2 | 3 |
| HBM _contig1074439 _s372022c11 _gene1<br>HBM _contig1164696 _s393417c29 _gene2<br>HBM _contig396545 _s178492 _gene1<br>HBM _contig434052 _s198679c8 _gene2<br>HBM _contig945098 _s342802c4 _gene3 | 6-phosphofructokinase | 2.7.1.11 | 5 |
| HBM _contig1302790 _s414786c2 _gene1<br>HBM _contig1374823 _s472809c3 _gene1<br>HBM _contig289576 _s127312c17 _gene3 | 6-phosphogluconate dehydrogenase | 1.1.1.44 | 3 |
| HBM _contig368493 _s163834c3 _gene1 | 6-phosphogluconolactonase | 3.1.1.31 | 1 |
| HBM _contig338536 _s149176c3 _gene1<br>HBM_contig407283_s184142c3_gene1<br>HBM_contig440778_s202339_gene1 | Acetyl-CoA C-acetyltransferase | 2.3.1.9 | 3 |
| HBM _contig298278 _s130904c3 _gene3<br>HBM _contig670075 _s281714c1 _gene1<br>HBM _contig134158 _s58687c27 _gene1<br>HBM _contig355008 _s157135c8 _gene1<br>HBM _contig438518 _s201108c18 _gene1<br>HBM _contig1494088 _s582224c2 _gene2 | Acid phosphatase | 3.1.3.2 | 12 |

| | | | |
|---|---|---|---|
| HBM _contig323185 _s142103c31 _gene1<br>HBM _contig348258 _s153791c20 _gene1<br>HBM _contig1409221 _s506095c5 _gene3<br>HBM _contig1016681 _s361260c5 _gene1<br>HBM _contig431766 _s197470c4 _gene1<br>HBM _contig1409222 _s506095c6 _gene1 | | | |
| HBM _contig147570 _s64869c11 _gene1<br>HBM _contig907698 _s335276c27 _gene1 | Aconitate hydratase | 4.2.1.3 | 2 |
| HBM _contig1292498 _s410603c18 _gene2<br>HBM _contig1318907 _s424517c3 _gene1 | Aldose 1-epimerase | 5.1.3.3 | 2 |
| HBM _contig318329 _s139806c20 _gene1<br>HBM_contig1318212_s423994c4_gene1 | Alpha, alpha-trehalase | 3.2.1.28 | 2 |
| HBM _contig285982 _s125966c2 _gene2<br>HBM _contig393825 _s177072c1 _gene1 | Alpha-amylase | 3.2.1.1 | 2 |
| HBM _contig1022739 _s362558c6 _gene2<br>HBM _contig286418 _s126148c2 _gene1 | Alpha-glucosidase | 3.2.1.20 | 2 |
| HBM _contig1357960 _s456977c8 _gene3<br>HBM _contig298249 _s130891c6 _gene1<br>HBM _contig340487 _s150149c5 _gene1<br>HBM _contig756485 _s300037c2 _gene5 | ATP citrate synthase | 2.3.3.8 | 4 |
| HBM_contig1038211_s365857c8_gene3<br>HBM_contig1038212_s365857c9_gene1<br>HBM_contig1161305_s392766c5_gene2<br>HBM_contig1302222_s414481c14_gene1<br>HBM_contig1348390_s448422c26_gene1<br>HBM_contig264692_s117484c7_gene1<br>HBM_contig274496_s121527c2_gene1<br>HBM_contig355900_s157502c9_gene1<br>HBM _contig1302222 _s414481c14 _gene1<br>HBM _contig1348390 _s448422c26 _gene1<br>HBM _contig264692 _s117484c7 _gene1<br>HBM _contig274496 _s121527c2 _gene1<br>HBM _contig313150 _s137410c19 _gene1<br>HBM _contig355900 _s157502c9 _gene1 | Hexokinase/Glucokinase | 2.7.1.1/<br>2.7.1.2 | 14 |
| HBM _contig1289509 _s409944c2 _gene1<br>HBM _contig1303515 _s415144c5 _gene1<br>HBM _contig1324950 _s428974c9 _gene1<br>HBM _contig1345306 _s445741c4 _gene1<br>HBM _contig1352768 _s452278c9 _gene1<br>HBM _contig1357953 _s456977c1 _gene1<br>HBM _contig232810 _s103616c2 _gene3<br>HBM _contig232867 _s103635c14 _gene1<br>HBM _contig299515 _s131427c8 _gene1<br>HBM _contig303742 _s133305c3 _gene1<br>HBM _contig336329 _s148152c12 _gene1 | β-fructofuranosidase/ Fructan β-fructosidase | 3.2.1.26/<br>3.2.1.80 | 34 |

| | | | |
|---|---|---|---|
| HBM _contig425247 _s193932c4 _gene2 | | | |
| HBM_contig09843_s04035c2_gene1 | | | |
| HBM_contig1030687_s364424c2_gene1 | | | |
| HBM_contig1030688_s364424c3_gene1 | | | |
| HBM_contig1119081_s384118c2_gene1 | | | |
| HBM_contig1119082_s384118c3_gene1 | | | |
| HBM_contig1303515_s415144c5_gene1 | | | |
| HBM_contig1308208_s417615c6_gene2 | | | |
| HBM_contig1352768_s452278c9_gene1 | | | |
| HBM_contig1357953_s456977c1_gene1 | | | |
| HBM_contig232810_s103616c2_gene3 | | | |
| HBM_contig279164_s123369c13_gene5 | | | |
| HBM_contig283350_s124975c7_gene1 | | | |
| HBM_contig299515_s131427c8_gene1 | | | |
| HBM_contig303742_s133305c3_gene1 | | | |
| HBM_contig323410_s142207c17_gene1 | | | |
| HBM_contig326170_s143445_gene1 | | | |
| HBM_contig336323_s148152c6_gene1 | | | |
| HBM_contig336327_s148152c10_gene2 | | | |
| HBM_contig336329_s148152c12_gene1 | | | |
| HBM_contig369602_s164404_gene1 | | | |
| HBM_contig425247_s193932c4_gene2 | | | |
| HBM_contig819355_s316235_gene1 | | | |
| HBM _contig1322037 _s426750c3 _gene1 | Carbonate dehydratase | 4.2.1.1 | 5 |
| HBM _contig1352352 _s451908c3 _gene2 | | | |
| HBM _contig285737 _s125861c3 _gene1 | | | |
| HBM _contig343186 _s151438c8 _gene1 | | | |
| HBM _contig383816 _s171800 _gene1 | | | |
| HBM _contig366916 _s163044c2 _gene1 | Citrate (Si) synthase | 2.3.3.1 | 1 |
| HBM_contig1289286_s409907c6_gene1 | Rubber cis-polyprenylcistransferase | 2.5.1.20 | 3 |
| HBM_contig272314_s120676_gene1 | | | |
| HBM_contig919895_s338071c2_gene1 | | | |
| HBM _contig257034 _s114160c16 _gene1 | Dihydrolipoamide dehydrogenase | 1.8.1.4 | 4 |
| HBM _contig316009 _s138773 _gene1 | | | |
| HBM _contig393998 _s177156c10 _gene1 | | | |
| HBM _contig484019 _s225263c9 _gene1 | | | |
| HBM_contig1311115_s419277c5_gene1 | Dimethylallyltranstransferase | 2.5.1.1 | 2 |
| HBM_contig526985_s245906c4_gene1 | | | |
| HBM _contig656003 _s279612c2 _gene3 | Diphosphomevalonate decarboxylase | 4.1.1.33 | 3 |
| HBM_contig1061580_s369994_gene1 | | | |
| HBM_contig1294315_s411141c4_gene1 | | | |
| HBM _contig1305637 _s416164c2 _gene1 | Farnesyl diphosphate synthase | 2.5.1.10 | 4 |
| HBM_contig657916_s279871c2_gene2 | | | |
| HBM_contig290987_s127806c1_gene1 | | | |
| HBM_contig290988_s127806c2_gene1 | | | |

| | | | |
|---|---|---|---|
| HBM _contig1311115 _s419277c5 _gene1<br>HBM_contig253780_s112770c3_gene1<br>HBM_contig1292487_s410603c7_gene1<br>HBM_contig1298993_s412919c4_gene1<br>HBM_contig550434_s255571_gene1<br>HBM_contig324001_gene1<br>HBM_contig324027_gene1 | Geranyl geranyl diphosphate synthase | 2.5.1.29 | 7 |
| HBM _contig1289171 _s409884c2 _gene1<br>HBM _contig1303826 _s415277c9 _gene1<br>HBM _contig1322658 _s427165c3 _gene3<br>HBM _contig1339227 _s440512c5 _gene3<br>HBM _contig180992 _s80187c5 _gene1<br>HBM _contig316188 _s138852c6 _gene2<br>HBM _contig320635 _s140865c2 _gene1<br>HBM _contig339478 _s149632c15 _gene2<br>HBM _contig522414 _s243848c9 _gene1 | Fructose-bisphosphate aldolase | 4.1.2.13 | 9 |
| HBM _contig214134 _s95238c3 _gene1<br>HBM _contig285749 _s125863c8 _gene2<br>HBM _contig391064 _s175615c5 _gene1<br>HBM _contig418176 _s190089c6 _gene1<br>HBM _contig743614 _s296819c6 _gene1 | Glucose 1-phosphate adenyl transferase | 2.7.7.27 | 5 |
| HBM _contig1357958 _s456977c6 _gene3<br>HBM _contig276796 _s122460c2 _gene1 | Glucose-6-phosphate 1-dehydrogenase | 1.1.1.49 | 2 |
| HBM_contig339226_s149517c1_gene1<br>HBM_contig1313987_s421061c3_gene1 | Glucose-6-phosphate 1-epimerase | 5.1.3.15 | 2 |
| HBM_contig362889_s161039c11_gene1 | Glucose-6-phosphate isomerase | 5.3.1.9 | 1 |
| HBM _contig1288678 _s409802c20 _gene2<br>HBM _contig1340694 _s441737c10 _gene1<br>HBM _contig218121 _s97036c9 _gene1<br>HBM _contig342711 _s151221c4 _gene1<br>HBM _contig130132 _s56830c9 _gene1<br>HBM _contig163761 _s72322c28 _gene1<br>HBM _contig474498 _s220352c2 _gene1 | Glyceraldehyde 3-phosphate dehydrogenase | 1.2.1.12 | 7 |
| HBM _contig342780 _s151248c4 _gene1 | Glycogen (starch) phosphorylase | 2.4.1.1 | 1 |
| HBM _contig1290182 _s410061c3 _gene1<br>HBM _contig269042 _s119264c2 _gene1<br>HBM _contig411305 _s186349c9 _gene1<br>HBM _contig454459 _s209676c4 _gene1<br>HBM_contig382803_s171253_gene1 | Hydroxymethyl glutaryl-CoA reductase | 1.1.1.34 | 5 |
| HBM _contig1303558 _s415149c3 _gene1<br>HBM_contig265284_s117727c3_gene4 | Hydroxymethylglutaryl-CoA synthase | 2.3.3.10 | 2 |
| HBM _contig243087 _s108198c7 _gene1<br>HBM _contig354346 _s156820c5 _gene1 | Isocitrate dehydrogenase | 1.1.1.42 | 2 |
| HBM _contig501437 _s233949c28 _gene1<br>HBM_contig326011_s143366_gene1 | Isopentenyl-diphosphate delta-isomerase | 5.3.3.2 | 3 |

| | | | |
|---|---|---|---|
| HBM_contig284636_s125486_gene1 | | | |
| HBM _contig01222 _s00535c3 _gene1 | L-malate hydro-lyase/ fumarate hydratase | 4.2.1.2 | 1 |
| HBM_contig1293296_s410821c9_gene1<br>HBM_contig1295328_s411484c1_gene1<br>HBM_contig284161_s125295c10_gene2<br>HBM_contig332061_s146156c4_gene2<br>HBM_contig675650_s282628_gene1<br>HBM _contig1293296 _s410821c9 _gene1<br>HBM _contig1295328 _s411484c1 _gene1<br>HBM _contig284161 _s125295c10 _gene2<br>HBM _contig332061 _s146156c4 _gene2<br>HBM _contig675650 _s282628 _gene1 | Malate dehydrogenase | 1.1.1.37 | 10 |
| HBM_contig1324605_s428704c13_gene4<br>HBM _contig285159 _s125660c12 _gene1<br>HBM _contig673366 _s282240c3 _gene1<br>HBM _contig721178 _s292728c3 _gene1<br>HBM_contig285159_s125660c12_gene1<br>HBM_contig673366_s282240c3_gene1<br>HBM_contig721178_s292728c3_gene1 | Malate dehydrogenase (decarboxylating)/<br>Malate dehydrogenase (oxaloacetate-decarboxylating) | 1.1.1.39/<br>1.1.1.40 | 7 |
| HBM_contig1295671_s411600c1_gene1<br>HBM_contig1300792_s413756c8_gene1 | Mevalonate kinase | 2.7.1.36 | 2 |
| HBM_contig819783_s316335c3_gene1<br>HBM_contig278880_s123280_gene1<br>HBM_contig362882_s161039c4_gene3 | Mitochondrial dicarboxylate carrier protein (DIC) | na | 3 |
| HBM_contig325647_s143203_gene1 | Monocarboxylate porter (MCP) | na | 1 |
| HBM_contig113175_s49095c7_gene1<br>HBM_contig1289350_s409914c30_gene1<br>HBM_contig1293510_s410890c6_gene1<br>HBM_contig1294848_s411334c2_gene2<br>HBM_contig205504_s91271c2_gene1<br>HBM_contig246731_s109760c5_gene1<br>HBM_contig253707_s112736c8_gene1<br>HBM_contig300103_s131701_gene1<br>HBM_contig301843_s132492c5_gene1<br>HBM_contig335154_s147607c4_gene1<br>HBM_contig338935_s149375c12_gene1<br>HBM_contig369354_s164271_gene1<br>HBM_contig377316_s168363c3_gene1<br>HBM_contig383251_s171496c1_gene1<br>HBM_contig383942_s171864_gene1<br>HBM_contig392526_s176392c5_gene1<br>HBM_contig395725_s178055_gene1<br>HBM_contig408839_s185016_gene1<br>HBM_contig414079_s187868_gene1<br>HBM_contig446109_s205232c1_gene1 | Monosaccharide transporter (MT) | na | 24 |

| | | | |
|---|---|---|---|
| HBM_contig446110_s205232c2_gene2<br>HBM_contig656790_gene1<br>HBM_contig757308_s300336c4_gene2<br>HBM_contig766992_s302901c1_gene1 | | | |
| HBM _contig303759 _s133310c8 _gene1 | NADP-dependentglyceraldehyde-3-phosphatedehydrogenase | 1.2.1.9 | 1 |
| HBM_contig1354496_s453822c10_gene2<br>HBM_contig231307_s102930c15_gene1<br>HBM_contig302254_s132663c8_gene1<br>HBM_contig728764_s293779c7_gene1<br>HBM _contig1354496 _s453822c10 _gene2<br>HBM _contig231307 _s102930c15 _gene1<br>HBM _contig302254 _s132663c8 _gene1 | Oxoglutarate:malate antiporter (OMT) | na | 7 |
| HBM_contig1302285_s414518c3_gene1 | Dicarboxylate/Tricarboxylate carrier protein (DTC) | na | 1 |
| HBM_contig1029566_s364163_gene1<br>HBM_contig1289192_s409889c5_gene1<br>HBM_contig134158_s58687c27_gene1<br>HBM_contig22381_s09035c12_gene1<br>HBM_contig325046_s142945_gene1 | Phosphoenolpyruvate-phosphatase | 3.1.3.60 | 5 |
| HBM_contig1305977_s416315c19_gene1<br>HBM _contig422634 _s192523c3 _gene2 | Phosphoglucomutase | 5.4.2.2 | 2 |
| HBM _contig747716 _s298021c22 _gene1 | Phosphoglycerate kinase | 2.7.2.3 | 1 |
| HBM _contig323198 _s142103c44 _gene1<br>HBM _contig349580 _s154385c27 _gene1<br>HBM _contig389245 _s174714c12 _gene1 | Phosphoglycerate mutase | 5.4.2.1 | 3 |
| HBM _contig433979 _s198650c9 _gene1<br>HBM_contig227302_s101115c1_gene1<br>HBM_contig297356_s130487_gene1 | Phosphomevalonatekinase | 2.7.4.2 | 3 |
| HBM _contig295239 _s129579c5 _gene1 | Phosphopyruvate hydratase (enolase) | 4.2.1.11 | 1 |
| HBM_contig1289256_s409900_gene1<br>HBM_contig298771_s131111c1_gene1 | Oligo-1,6-glucosidase | 3.2.1.10 | 2 |
| HBM _contig1290187 _s410061c8 _gene3<br>HBM _contig334050 _s147096c2 _gene1 | Pyrophosphate--fructose-6-phosphate 1-phosphotransferase | 2.7.1.90 | 2 |
| HBM _contig433980 _s198650c10 _gene1<br>HBM _contig1301528 _s414125c2 _gene1<br>HBM _contig1311068 _s419250c5 _gene1<br>HBM _contig30266 _s12350c4 _gene1<br>HBM _contig1290757 _s410195c9 _gene1<br>HBM _contig1294128 _s411073c7 _gene1 | Pyruvate dehydrogenase | 1.2.4.1 | 6 |
| HBM_contig101953_s43997c3_gene2<br>HBM_contig299370_s131382_gene1<br>HBM_contig397018_s178729c12_gene1<br>HBM_contig402074_s181422c8_gene1 | Dihydrolipoyllysine-residue acetyl transferase | 2.3.1.12 | 4 |
| HBM _contig1026725 _s363504c2 _gene2<br>HBM _contig1297534 _s412313c2 _gene1 | Pyruvate kinase | 2.7.1.40 | 8 |

| | | | |
|---|---|---|---|
| HBM _contig1304126 _s415411c3 _gene1<br>HBM _contig1344950 _s445443c5 _gene5<br>HBM _contig136768 _s59843c7 _gene1<br>HBM _contig290846 _s127748c7 _gene1<br>HBM _contig383462 _s171609c3 _gene1<br>HBM _contig294978 _s129482c1 _gene1 | | | |
| HBM _contig114975 _s49906c13 _gene1<br>HBM _contig1312636 _s420192c3 _gene1<br>HBM _contig346662 _s153048c28 _gene3 | Ribose 5-phosphate isomerase A | 5.3.1.6 | 3 |
| HBM _contig304780 _s133706c10 _gene2 | Ribulose-phosphate 3-epimerase | 5.1.3.1 | 1 |
| HBM_contig1314787_s421584c1_gene1<br>HBM_contig1314789_s421584c3_gene1<br>HBM_contig1352860_s452368_gene1<br>HBM_contig1432969_s529413_gene1<br>HBM_contig210302_s93430_gene1<br>HBM_contig246314_s109563_gene1<br>HBM_contig285004_s125605_gene1<br>HBM_contig289068_s127107_gene1<br>HBM_contig331924_s146093c6_gene1<br>HBM_contig331929_s146093c11_gene1<br>HBM_contig420863_s191570c9_gene1<br>HBM_contig953976_gene1 | Rubber elongation factor (REF) | na | 12 |
| HBM_contig1291168_s410297_gene2<br>HBM_contig1314791_s421584c5_gene1<br>HBM_contig1351062_s450759c14_gene1<br>HBM_contig1445641_s541695c60_gene1<br>HBM_contig314926_s138259_gene1<br>HBM_contig331920_s146093c2_gene1<br>HBM_contig331921_s146093c3_gene1<br>HBM_contig331923_s146093c5_gene1<br>HBM_contig364556_s161856_gene1<br>HBM_contig878396_gene1 | Small rubber particle protein (SRPP) | na | 10 |
| HBM _contig164849 _s72856c4 _gene2<br>HBM_contig230057_s102351c21_gene 1<br>HBM_contig306687_s134504c10_gene 1<br>HBM_contig332846_s146521c1_gene 1<br>HBM_contig943860_s342523c1_gene 1<br>HBM_contig1289127_s409874c9_gene 1<br>HBM_contig1318935_s424517c31_gene 1 | Starch synthase | 2.4.1.21 | 7 |
| HBM _contig1348382 _s448422c18 _gene1<br>HBM _contig309950 _s135961c7 _gene1<br>HBM _contig329252 _s144873c5 _gene3 | Phosphoenolpyruvate carboxykinase | 4.1.1.49 | 3 |
| HBM _contig290756 _s127709c10 _gene1<br>HBM _contig509596 _s237846c7 _gene1 | Succinate dehydrogenase | 1.3.5.1 | 2 |
| HBM_contig348246_s153791c8_gene2 | Succinyl-CoA ligase | 6.2.1.5 | 2 |

| | | | |
|---|---|---|---|
| HBM_contig363653_s161426_gene1 | | | |
| HBM_contig227922_s101408c5_gene1 | Pyruvate decarboxylase | 4.1.1.1 | 2 |
| HBM_contig369579_s164395c1_gene1 | | | |
| HBM_contig213287_s94844c20_gene1 | Aldehyde dehydrogenase | 1.2.1.3 | 3 |
| HBM_contig325083_s142963_gene1 | | | |
| HBM_contig378330_s168914c2_gene2 | | | |
| HBM_contig378047_s168772_gene1 | Alcohol dehydrogenase | 1.1.1.1 | 2 |
| HBM_contig1292652_s410638c8_gene2 | | | |
| HBM_contig63343_s26589c5_gene1 | l-lactate dehydrogenase | 1.1.1.27 | 2 |
| HBM_contig1254051_s405310c12_gene1 | | | |
| HBM_contig202157_s89781c2_gene1 | Acetyl-CoA ligase | 6.2.1.1 | 2 |
| HBM_contig770884_s304118c4_gene1 | | | |
| HBM_contig403677_s182258c2_gene1 | Trehalose phosphatase | 3.1.3.12 | 10 |
| HBM_contig1289511_s409944c4_gene3 | | | |
| HBM_contig371632_s165417c5_gene1 | | | |
| HBM_contig1324951_s428974c10_gene1 | | | |
| HBM_contig1420163_s516850c9_gene1 | | | |
| HBM_contig451554_s208132c13_gene1 | | | |
| HBM_contig353116_s156220_gene1 | | | |
| HBM_contig431760_s197468c3_gene3 | | | |
| HBM_contig1288955_s409848c4_gene1 | | | |
| HBM_contig1332985_s435332c13_gene1 | | | |
| HBM_contig1352489_s452038_gene1 | 4-hydroxy-3-methylbut-2-enyl-diphosphate synthase | 1.17.7.1 | 3 |
| HBM_contig328275_s144399c2_gene1 | | | |
| HBM_contig433089_s198173c7_gene1 | | | |
| HBM_contig1289509_s409944c2_gene1 | Sucrose -fructosyltransferase | 2.4.1.99 | 2 |
| HBM_contig1345306_s445741c4_gene1 | | | |
| HBM _contig1288687 _s409803c1 _gene1 | Sucrose phosphate phosphatase | 3.1.3.24 | 2 |
| HBM_contig272605_s120794c27_gene1 | | | |
| HBM _contig757629 _s300436c6 _gene1 | Sucrose phosphate synthase | 2.4.1.14 | 4 |
| HBM_contig771203_s304239c15_gene1 | | | |
| HBM_contig1463859_s558603c4_gene1 | | | |
| HBM_contig355822_s157485c2_gene1 | | | |
| HBM _contig01226 _s00535c7 _gene1 | Sucrose synthase | 2.4.1.13 | 8 |
| HBM_contig1310976_s419196c4_gene1 | | | |
| HBM_contig281038_s124058c3_gene2 | | | |
| HBM_contig395231_s177780c5_gene1 | | | |
| HBM_contig513220_s239591c6_gene3 | | | |
| HBM_contig314089_s137872_gene1 | | | |
| HBM_contig1294277_s411129_gene1 | | | |
| HBM_contig281039_s124058c4_gene1 | | | |
| HBM _contig47165 _s19567c3 _gene2 | Sucrose transporter (SUT) | na | 14 |
| HBM_contig1109905_s381969_gene1 | | | |
| HBM_contig1289974_s410033c6_gene1 | | | |
| HBM_contig1299594_s413194c9_gene1 | | | |

| | | | |
|---|---|---|---|
| HBM_contig1301388_s414043_gene1 | | | |
| HBM_contig1323260_s427655_gene1 | | | |
| HBM_contig332408_s146322c13_gene1 | | | |
| HBM_contig332409_s146322c14_gene1 | | | |
| HBM_contig380380_s169971_gene1 | | | |
| HBM_contig429984_s196523_gene1 | | | |
| HBM_contig43409_s17931_gene1 | | | |
| HBM_contig47165_s19567c3_gene2 | | | |
| HBM_contig654533_s279397c2_gene1 | | | |
| HBM _contig205504 _s91271c2 _gene1 | | | |
| HBM _contig346208 _s152843c5 _gene1 | Transaldolase | 2.2.1.2 | 1 |
| HBM _contig1125285 _s385576c12 _gene1 | Transketolase | 2.2.1.1 | 2 |
| HBM _contig324592 _s142744 _gene1 | | | |
| HBM _contig277898 _s122911c5 _gene1 | Trehalose-6-phosphate synthase | 2.4.1.15 | 2 |
| HBM_contig273515_s121147c18_gene2 | | | |
| HBM _contig1317309 _s423378c2 _gene1 | Triosephosphate isomerase | 5.3.1.1 | 2 |
| HBM _contig1364363 _s462960c8 _gene1 | | | |
| HBM _contig354344 _s156820c3 _gene1 | UTP-glucose-1-phosphateuridylyltransferase | 2.7.7.9 | 1 |
| HBM_contig1356982_s456101_gene1 | Undecaprenyl-diphosphate synthase | 2.5.1.31 | 5 |
| HBM_contig379004_s169268c2_gene1 | | | |
| HBM_contig1329326_s432359c2_gene3 | | | |
| HBM_contig379006_s169268c4_gene1 | | | |
| HBM_contig232787_s103605c15_gene1 | | | |
| HBM_contig1311114_s419277c4_gene1 | Galtase | 2.4.1.- | 5 |
| HBM_contig1323470_s427819c4_gene1 | | | |
| HBM_contig1013854_s360717c11_gene3 | | | |
| HBM_contig1299176_s413002_gene1 | | | |
| HBM_contig1311113_s419277c3_gene2 | | | |

**Table S17.** Rubber biosynthesis related genes of *H. brasiliensis* in comparison to *Parthenium argentatum* (guayule) ESTs

| Gene model | No. of EST hits[a] | Lowest E-value[b] | Greatest identity %[b] |
|---|---|---|---|
| HBM_contig1297534_s412313c2_gene1 | 8 | 4.63E-133 | 98 |
| HBM_contig362889_s161039c11_gene1 | 2 | 2.43E-24 | 98 |
| HBM_contig1352489_s452038_gene1 | 5 | 1.67E-35 | 97 |
| HBM_contig1288739_s409811c4_gene1 | 9 | 3.72E-95 | 96 |
| HBM_contig1374823_s472809c3_gene1 | 7 | 3.57E-88 | 96 |
| HBM_contig301843_s132492c5_gene1 | 5 | 4.19E-85 | 96 |
| HBM_contig340487_s150149c5_gene1 | 6 | 8.66E-111 | 96 |
| HBM_contig756485_s300037c2_gene5 | 5 | 1.23E-109 | 96 |
| HBM_contig1357960_s456977c8_gene3 | 44 | 8.16E-124 | 95 |
| HBM_contig163761_s72322c28_gene1 | 35 | 5.62E-94 | 95 |
| HBM_contig770884_s304118c4_gene1 | 18 | 6.89E-53 | 95 |
| HBM_contig1288678_s409802c20_gene2 | 37 | 4.02E-122 | 94 |
| HBM_contig1301528_s414125c2_gene1 | 7 | 1.99E-47 | 94 |
| HBM_contig328275_s144399c2_gene1 | 9 | 6.93E-126 | 94 |
| HBM_contig339478_s149632c15_gene2 | 21 | 1.00E-117 | 94 |
| HBM_contig342711_s151221c4_gene1 | 37 | 3.20E-119 | 94 |
| HBM_contig433089_s198173c7_gene1 | 9 | 1.37E-125 | 94 |
| HBM_contig766992_s302901c1_gene1 | 6 | 1.06E-83 | 94 |
| HBM_contig1302285_s414518c3_gene1 | 39 | 2.12E-98 | 93 |
| HBM_contig1302790_s414786c2_gene1 | 8 | 7.77E-118 | 93 |
| HBM_contig147570_s64869c11_gene1 | 16 | 2.14E-114 | 93 |
| HBM_contig294978_s129482c1_gene1 | 8 | 1.78E-96 | 93 |
| HBM_contig338935_s149375c12_gene1 | 5 | 9.15E-77 | 93 |
| HBM_contig346208_s152843c5_gene1 | 11 | 6.42E-113 | 93 |
| HBM_contig522414_s243848c9_gene1 | 23 | 5.14E-135 | 93 |
| HBM_contig675650_s282628_gene1 | 11 | 1.46E-103 | 93 |
| HBM_contig1289171_s409884c2_gene1 | 23 | 3.97E-135 | 92 |
| HBM_contig1299176_s413002_gene1 | 22 | 2.30E-98 | 92 |
| HBM_contig130132_s56830c9_gene1 | 35 | 1.47E-120 | 92 |
| HBM_contig1307528_s417190c7_gene1 | 7 | 1.17E-52 | 92 |
| HBM_contig180992_s80187c5_gene1 | 21 | 1.66E-131 | 92 |
| HBM_contig289576_s127312c17_gene3 | 9 | 5.65E-115 | 92 |
| HBM_contig295239_s129579c5_gene1 | 22 | 3.95E-127 | 92 |
| HBM_contig343183_s151438c5_gene1 | 4 | 1.09E-60 | 92 |
| HBM_contig907698_s335276c27_gene1 | 10 | 3.22E-113 | 92 |
| HBM_contig1326688_s430296c2_gene2 | 10 | 6.66E-89 | 91 |
| HBM_contig1340694_s441737c10_gene1 | 36 | 8.63E-119 | 91 |
| HBM_contig217879_s96943c3_gene1 | 9 | 4.76E-88 | 91 |
| HBM_contig326011_s143366_gene1 | 7 | 8.30E-79 | 91 |
| HBM_contig484019_s225263c9_gene1 | 13 | 1.91E-110 | 91 |
| HBM_contig1324605_s428704c13_gene4 | 7 | 5.58E-106 | 90 |
| HBM_contig1339227_s440512c5_gene3 | 22 | 1.09E-130 | 90 |

| | | | |
|---|---|---|---|
| HBM_contig136768_s59843c7_gene1 | 10 | 4.18E-111 | 90 |
| HBM_contig290988_s127806c2_gene1 | 2 | 9.62E-11 | 90 |
| HBM_contig354346_s156820c5_gene1 | 7 | 3.22E-95 | 90 |
| HBM_contig382299_s170997c9_gene2 | 4 | 1.16E-130 | 90 |
| HBM_contig411305_s186349c9_gene1 | 8 | 1.02E-96 | 90 |
| HBM_contig418176_s190089c6_gene1 | 5 | 8.17E-103 | 90 |
| HBM_contig454459_s209676c4_gene1 | 17 | 2.33E-93 | 90 |
| HBM_contig743614_s296819c6_gene1 | 15 | 1.76E-107 | 90 |
| HBM_contig1292652_s410638c8_gene2 | 34 | 6.96E-104 | 89 |
| HBM_contig1322658_s427165c3_gene3 | 22 | 1.09E-129 | 89 |
| HBM_contig1352768_s452278c9_gene1 | 2 | 7.10E-123 | 89 |
| HBM_contig284161_s125295c10_gene2 | 10 | 1.93E-107 | 89 |
| HBM_contig297371_s130496_gene1 | 15 | 7.42E-89 | 89 |
| HBM_contig303742_s133305c3_gene1 | 3 | 1.25E-122 | 89 |
| HBM_contig323410_s142207c17_gene1 | 5 | 2.93E-123 | 89 |
| HBM_contig354344_s156820c3_gene1 | 12 | 1.65E-97 | 89 |
| HBM_contig378330_s168914c2_gene2 | 12 | 4.61E-103 | 89 |
| HBM_contig1290187_s410061c8_gene3 | 19 | 2.89E-63 | 88 |
| HBM_contig1293296_s410821c9_gene1 | 10 | 3.69E-107 | 88 |
| HBM_contig1310976_s419196c4_gene1 | 5 | 6.73E-111 | 88 |
| HBM_contig1311068_s419250c5_gene1 | 6 | 1.13E-71 | 88 |
| HBM_contig257034_s114160c16_gene1 | 13 | 4.10E-114 | 88 |
| HBM_contig269042_s119264c2_gene1 | 9 | 1.60E-106 | 88 |
| HBM_contig281038_s124058c3_gene2 | 5 | 8.27E-111 | 88 |
| HBM_contig303759_s133310c8_gene1 | 10 | 6.79E-100 | 88 |
| HBM_contig425247_s193932c4_gene2 | 2 | 5.80E-124 | 88 |
| HBM_contig474498_s220352c2_gene1 | 10 | 7.74E-101 | 88 |
| HBM_contig1290182_s410061c3_gene1 | 9 | 2.08E-90 | 87 |
| HBM_contig1311114_s419277c4_gene1 | 11 | 1.90E-46 | 87 |
| HBM_contig1344950_s445443c5_gene5 | 14 | 3.55E-51 | 87 |
| HBM_contig243087_s108198c7_gene1 | 5 | 7.55E-102 | 87 |
| HBM_contig285159_s125660c12_gene1 | 8 | 1.43E-123 | 87 |
| HBM_contig01222_s00535c3_gene1 | 3 | 4.14E-91 | 86 |
| HBM_contig1295328_s411484c1_gene1 | 10 | 6.91E-105 | 86 |
| HBM_contig1305637_s416164c2_gene1 | 4 | 5.90E-104 | 86 |
| HBM_contig1343951_s444526c6_gene1 | 9 | 8.48E-82 | 86 |
| HBM_contig227922_s101408c5_gene1 | 4 | 3.99E-44 | 86 |
| HBM_contig298249_s130891c6_gene1 | 7 | 4.29E-107 | 86 |
| HBM_contig334050_s147096c2_gene1 | 9 | 1.38E-121 | 86 |
| HBM_contig382803_s171253_gene1 | 10 | 4.62E-104 | 86 |
| HBM_contig945098_s342802c4_gene3 | 16 | 1.12E-112 | 86 |
| HBM_contig01226_s00535c7_gene1 | 8 | 8.19E-119 | 85 |
| HBM_contig272605_s120794c27_gene1 | 3 | 9.23E-26 | 85 |
| HBM_contig278880_s123280_gene1 | 51 | 1.65E-46 | 85 |
| HBM_contig304780_s133706c10_gene2 | 4 | 2.42E-98 | 85 |
| HBM_contig349580_s154385c27_gene1 | 4 | 6.90E-104 | 85 |

| | | | |
|---|---|---|---|
| HBM_contig407283_s184142c3_gene1 | 4 | 1.70E-59 | 85 |
| HBM_contig434052_s198679c8_gene2 | 16 | 8.92E-116 | 85 |
| HBM_contig440778_s202339_gene1 | 17 | 2.69E-57 | 85 |
| HBM_contig501437_s233949c28_gene1 | 7 | 2.25E-95 | 85 |
| HBM_contig1061580_s369994_gene1 | 5 | 5.86E-53 | 84 |
| HBM_contig1074439_s372022c11_gene1 | 16 | 2.14E-114 | 84 |
| HBM_contig300103_s131701_gene1 | 6 | 7.80E-33 | 84 |
| HBM_contig402074_s181422c8_gene1 | 9 | 1.53E-58 | 84 |
| HBM_contig1125285_s385576c12_gene1 | 8 | 4.61E-51 | 83 |
| HBM_contig113175_s49095c7_gene1 | 7 | 1.04E-36 | 83 |
| HBM_contig1254051_s405310c12_gene1 | 8 | 7.39E-101 | 83 |
| HBM_contig1289350_s409914c30_gene1 | 8 | 1.31E-32 | 83 |
| HBM_contig1294315_s411141c4_gene1 | 5 | 9.78E-28 | 83 |
| HBM_contig1303558_s415149c3_gene1 | 25 | 1.78E-117 | 83 |
| HBM_contig1303826_s415277c9_gene1 | 26 | 1.49E-119 | 83 |
| HBM_contig1312636_s420192c3_gene1 | 5 | 1.40E-96 | 83 |
| HBM_contig1339539_s440773c5_gene3 | 2 | 2.63E-53 | 83 |
| HBM_contig213287_s94844c20_gene1 | 3 | 5.18E-109 | 83 |
| HBM_contig276900_s122507c5_gene1 | 4 | 5.90E-101 | 83 |
| HBM_contig318055_s139710c8_gene1 | 16 | 4.62E-120 | 83 |
| HBM_contig338536_s149176c3_gene1 | 20 | 6.37E-111 | 83 |
| HBM_contig43409_s17931_gene1 | 14 | 2.25E-85 | 83 |
| HBM_contig656003_s279612c2_gene3 | 6 | 5.54E-90 | 83 |
| HBM_contig819783_s316335c3_gene1 | 53 | 1.76E-57 | 83 |
| HBM_contig114975_s49906c13_gene1 | 10 | 6.08E-100 | 82 |
| HBM_contig316188_s138852c6_gene2 | 22 | 5.59E-115 | 82 |
| HBM_contig320635_s140865c2_gene1 | 23 | 2.35E-116 | 82 |
| HBM_contig323198_s142103c44_gene1 | 3 | 1.43E-97 | 82 |
| HBM_contig63343_s26589c5_gene1 | 8 | 4.23E-98 | 82 |
| HBM_contig673366_s282240c3_gene1 | 9 | 2.10E-123 | 82 |
| HBM_contig721178_s292728c3_gene1 | 8 | 3.99E-105 | 82 |
| HBM_contig1294128_s411073c7_gene1 | 11 | 3.54E-28 | 81 |
| HBM_contig285749_s125863c8_gene2 | 8 | 2.54E-89 | 81 |
| HBM_contig302280_s132669c17_gene1 | 4 | 9.39E-68 | 81 |
| HBM_contig314089_s137872_gene1 | 7 | 5.06E-26 | 81 |
| HBM_contig316009_s138773_gene1 | 15 | 4.26E-90 | 81 |
| HBM_contig369579_s164395c1_gene1 | 3 | 6.98E-66 | 81 |
| HBM_contig1289192_s409889c5_gene1 | 4 | 5.09E-50 | 80 |
| HBM_contig1299594_s413194c9_gene1 | 18 | 3.10E-88 | 80 |
| HBM_contig1304126_s415411c3_gene1 | 10 | 6.68E-106 | 80 |
| HBM_contig1404580_s501548c30_gene1 | 7 | 1.93E-78 | 80 |
| HBM_contig279164_s123369c13_gene5 | 3 | 3.59E-63 | 80 |
| HBM_contig377316_s168363c3_gene1 | 9 | 1.39E-92 | 80 |
| HBM_contig290846_s127748c7_gene1 | 10 | 1.05E-105 | 79 |
| HBM_contig378047_s168772_gene1 | 37 | 4.09E-72 | 79 |
| HBM_contig392526_s176392c5_gene1 | 11 | 2.15E-91 | 79 |

| | | | |
|---|---|---|---|
| HBM_contig943860_s342523c1_gene1 | 5 | 2.89E-35 | 79 |
| HBM_contig1290757_s410195c9_gene1 | 10 | 1.39E-29 | 78 |
| HBM_contig202157_s89781c2_gene1 | 10 | 4.11E-45 | 78 |
| HBM_contig283350_s124975c7_gene1 | 3 | 9.10E-109 | 78 |
| HBM_contig299370_s131382_gene1 | 7 | 4.62E-59 | 78 |
| HBM_contig380380_s169971_gene1 | 9 | 3.19E-81 | 78 |
| HBM_contig529416_s246980c14_gene1 | 9 | 7.77E-79 | 78 |
| HBM_contig1364363_s462960c8_gene1 | 8 | 3.48E-104 | 77 |
| HBM_contig265284_s117727c3_gene4 | 6 | 7.87E-61 | 77 |
| HBM_contig1038212_s365857c9_gene1 | 3 | 1.41E-32 | 76 |
| HBM_contig274496_s121527c2_gene1 | 2 | 3.50E-53 | 76 |
| HBM_contig30266_s12350c4_gene1 | 7 | 1.66E-70 | 76 |
| HBM_contig355900_s157502c9_gene1 | 2 | 1.26E-53 | 76 |
| HBM_contig1302222_s414481c14_gene1 | 2 | 6.06E-53 | 75 |
| HBM_contig433979_s198650c9_gene1 | 4 | 3.18E-51 | 75 |
| HBM_contig1289974_s410033c6_gene1 | 7 | 3.90E-61 | 74 |
| HBM_contig1318907_s424517c3_gene1 | 13 | 1.30E-54 | 74 |
| HBM_contig332409_s146322c14_gene1 | 3 | 3.50E-07 | 74 |
| HBM_contig1295671_s411600c1_gene1 | 5 | 7.97E-38 | 73 |
| HBM_contig264692_s117484c7_gene1 | 4 | 1.58E-38 | 73 |
| HBM_contig290987_s127806c1_gene1 | 5 | 7.29E-73 | 73 |
| HBM_contig393998_s177156c10_gene1 | 12 | 1.89E-74 | 73 |
| HBM_contig420863_s191570c9_gene1 | 11 | 4.77E-50 | 73 |
| HBM_contig1317309_s423378c2_gene1 | 5 | 3.38E-95 | 72 |
| HBM_contig414079_s187868_gene1 | 13 | 1.14E-81 | 72 |
| HBM_contig513220_s239591c6_gene3 | 4 | 1.41E-90 | 72 |
| HBM_contig657916_s279871c2_gene2 | 8 | 5.12E-86 | 72 |
| HBM_contig232787_s103605c15_gene1 | 15 | 2.81E-62 | 71 |
| HBM_contig1288687_s409803c1_gene1 | 4 | 3.57E-64 | 70 |
| HBM_contig1300792_s413756c8_gene1 | 4 | 1.56E-38 | 70 |
| HBM_contig227302_s101115c1_gene1 | 3 | 7.09E-48 | 70 |
| HBM_contig329252_s144873c5_gene3 | 3 | 5.29E-85 | 70 |
| HBM_contig366145_s162700_gene1 | 4 | 9.31E-68 | 70 |
| HBM_contig397018_s178729c12_gene1 | 3 | 3.88E-13 | 70 |
| HBM_contig757308_s300336c4_gene2 | 8 | 3.82E-72 | 70 |
| HBM_contig1294277_s411129_gene1 | 4 | 3.20E-29 | 69 |
| HBM_contig1294848_s411334c2_gene2 | 7 | 1.46E-49 | 69 |
| HBM_contig1311115_s419277c5_gene1 | 16 | 1.93E-37 | 69 |
| HBM_contig1323470_s427819c4_gene1 | 10 | 3.58E-91 | 69 |
| HBM_contig1348382_s448422c18_gene1 | 1 | 1.84E-84 | 69 |
| HBM_contig1357953_s456977c1_gene1 | 3 | 1.64E-79 | 69 |
| HBM_contig309950_s135961c7_gene1 | 4 | 1.68E-83 | 69 |
| HBM_contig362882_s161039c4_gene3 | 33 | 3.01E-30 | 69 |
| HBM_contig281039_s124058c4_gene1 | 2 | 1.44E-28 | 68 |
| HBM_contig395231_s177780c5_gene1 | 3 | 2.68E-88 | 68 |
| HBM_contig654533_s279397c2_gene1 | 8 | 2.04E-65 | 68 |

| | | | |
|---|---|---|---|
| HBM_contig09843_s04035c2_gene1 | 4 | 5.80E-73 | 67 |
| HBM_contig1026725_s363504c2_gene2 | 5 | 8.11E-65 | 67 |
| HBM_contig1308223_s417615c21_gene1 | 3 | 5.26E-69 | 67 |
| HBM_contig1324950_s428974c9_gene1 | 38 | 2.67E-86 | 67 |
| HBM_contig1332985_s435332c13_gene1 | 3 | 1.57E-90 | 67 |
| HBM_contig306687_s134504c10_gene1 | 5 | 1.38E-41 | 67 |
| HBM_contig332061_s146156c4_gene2 | 7 | 8.04E-72 | 67 |
| HBM_contig363653_s161426_gene1 | 6 | 3.47E-28 | 67 |
| HBM_contig550434_s255571_gene1 | 13 | 2.98E-25 | 67 |
| HBM_contig1345306_s445741c4_gene1 | 32 | 1.01E-76 | 66 |
| HBM_contig1351062_s450759c14_gene1 | 10 | 1.95E-09 | 66 |
| HBM_contig273515_s121147c18_gene2 | 7 | 2.38E-91 | 66 |
| HBM_contig335154_s147607c4_gene1 | 5 | 1.60E-42 | 66 |
| HBM_contig1289509_s409944c2_gene1 | 32 | 3.70E-74 | 65 |
| HBM_contig1305977_s416315c19_gene1 | 4 | 1.58E-62 | 65 |
| HBM_contig299515_s131427c8_gene1 | 5 | 3.92E-80 | 64 |
| HBM_contig332846_s146521c1_gene1 | 5 | 3.84E-70 | 64 |
| HBM_contig396545_s178492_gene1 | 12 | 2.32E-88 | 64 |
| HBM_contig1030688_s364424c3_gene1 | 6 | 2.80E-75 | 63 |
| HBM_contig1119081_s384118c2_gene1 | 5 | 7.00E-76 | 63 |
| HBM_contig276796_s122460c2_gene1 | 5 | 2.19E-81 | 63 |
| HBM_contig919895_s338071c2_gene1 | 5 | 7.52E-44 | 63 |
| HBM_contig369602_s164404_gene1 | 3 | 7.71E-14 | 62 |
| HBM_contig1329326_s432359c2_gene3 | 9 | 2.21E-62 | 61 |
| HBM_contig246731_s109760c5_gene1 | 5 | 2.11E-48 | 61 |
| HBM_contig325046_s142945_gene1 | 5 | 3.10E-56 | 61 |
| HBM_contig47165_s19567c3_gene2 | 7 | 1.13E-55 | 61 |
| HBM_contig1303515_s415144c5_gene1 | 8 | 1.12E-79 | 60 |
| HBM_contig1352860_s452368_gene1 | 15 | 6.45E-15 | 60 |
| HBM_contig379006_s169268c4_gene1 | 15 | 1.48E-61 | 60 |
| HBM_contig383462_s171609c3_gene1 | 11 | 2.71E-72 | 60 |
| HBM_contig1308208_s417615c6_gene1 | 13 | 4.54E-10 | 59 |
| HBM_contig272314_s120676_gene1 | 8 | 8.30E-23 | 59 |
| HBM_contig325083_s142963_gene1 | 2 | 6.21E-65 | 59 |
| HBM_contig332408_s146322c13_gene1 | 8 | 1.76E-42 | 59 |
| HBM_contig1289286_s409907c6_gene1 | 10 | 9.39E-48 | 58 |
| HBM_contig232867_s103635c14_gene1 | 32 | 3.83E-65 | 58 |
| HBM_contig253780_s112770c3_gene1 | 3 | 1.78E-17 | 58 |
| HBM_contig364556_s161856_gene1 | 10 | 3.95E-56 | 58 |
| HBM_contig369354_s164271_gene1 | 5 | 1.80E-32 | 58 |
| HBM_contig446109_s205232c1_gene1 | 7 | 1.22E-69 | 58 |
| HBM_contig878396_gene1 | 9 | 9.53E-12 | 58 |
| HBM_contig1030687_s364424c2_gene1 | 5 | 5.68E-13 | 57 |
| HBM_contig246314_s109563_gene1 | 12 | 1.50E-17 | 57 |
| HBM_contig285737_s125861c3_gene1 | 8 | 6.96E-37 | 57 |
| HBM_contig324592_s142744_gene1 | 3 | 5.30E-29 | 57 |

| | | | |
|---|---|---|---|
| HBM_contig368493_s163834c3_gene1 | 5 | 2.31E-65 | 57 |
| HBM_contig379004_s169268c2_gene1 | 7 | 6.53E-56 | 57 |
| HBM_contig395725_s178055_gene1 | 3 | 1.38E-09 | 57 |
| HBM_contig819355_s316235_gene1 | 25 | 1.89E-59 | 57 |
| HBM_contig286418_s126148c2_gene1 | 12 | 3.38E-35 | 56 |
| HBM_contig336329_s148152c12_gene1 | 32 | 1.07E-70 | 56 |
| HBM_contig422634_s192523c3_gene1 | 8 | 4.66E-47 | 56 |
| HBM_contig1292487_s410603c7_gene1 | 11 | 6.86E-41 | 55 |
| HBM_contig253707_s112736c8_gene1 | 12 | 9.16E-69 | 55 |
| HBM_contig331923_s146093c5_gene1 | 12 | 1.48E-48 | 55 |
| HBM_contig348258_s153791c20_gene1 | 5 | 1.32E-20 | 55 |
| HBM_contig383816_s171800_gene1 | 8 | 6.39E-51 | 55 |
| HBM_contig979245_s352662c2_gene3 | 3 | 2.90E-20 | 55 |
| HBM_contig205504_s91271c2_gene1 | 10 | 1.70E-66 | 54 |
| HBM_contig391064_s175615c5_gene1 | 4 | 1.40E-42 | 54 |
| HBM_contig1314787_s421584c1_gene1 | 11 | 3.93E-19 | 53 |
| HBM_contig1323260_s427655_gene1 | 7 | 4.52E-65 | 53 |
| HBM_contig1352352_s451908c3_gene2 | 9 | 5.74E-49 | 53 |
| HBM_contig289068_s127107_gene1 | 13 | 2.44E-15 | 53 |
| HBM_contig313150_s137410c19_gene1 | 3 | 1.80E-33 | 53 |
| HBM_contig331921_s146093c3_gene1 | 9 | 2.31E-20 | 53 |
| HBM_contig331924_s146093c6_gene1 | 10 | 1.65E-22 | 53 |
| HBM_contig331929_s146093c11_gene1 | 14 | 1.74E-21 | 53 |
| HBM_contig429984_s196523_gene1 | 11 | 7.07E-65 | 53 |
| HBM_contig757629_s300436c6_gene1 | 5 | 1.48E-08 | 53 |
| HBM_contig1022739_s362558c6_gene2 | 12 | 8.39E-37 | 52 |
| HBM_contig343186_s151438c8_gene1 | 10 | 2.09E-55 | 52 |
| HBM_contig134158_s58687c27_gene1 | 6 | 1.10E-38 | 51 |
| HBM_contig1357958_s456977c6_gene3 | 4 | 7.03E-59 | 51 |
| HBM_contig22381_s09035c12_gene1 | 2 | 1.50E-39 | 51 |
| HBM_contig1314789_s421584c3_gene1 | 10 | 9.51E-24 | 50 |
| HBM_contig1356982_s456101_gene1 | 7 | 7.60E-12 | 50 |
| HBM_contig230057_s102351c21_gene1 | 4 | 7.89E-50 | 50 |
| HBM_contig336327_s148152c10_gene1 | 7 | 6.58E-06 | 50 |
| HBM_contig348246_s153791c8_gene1 | 5 | 6.54E-48 | 49 |
| HBM_contig526985_s245906c4_gene1 | 10 | 3.28E-44 | 49 |
| HBM_contig1322037_s426750c3_gene1 | 9 | 2.23E-35 | 48 |
| HBM_contig1445641_s541695c60_gene1 | 13 | 1.13E-28 | 48 |
| HBM_contig325647_s143203_gene1 | 10 | 3.19E-55 | 48 |
| HBM_contig336323_s148152c6_gene1 | 15 | 8.39E-33 | 48 |
| HBM_contig408839_s185016_gene1 | 13 | 3.68E-07 | 48 |
| HBM_contig431766_s197470c4_gene1 | 8 | 2.73E-37 | 48 |
| HBM_contig1016681_s361260c5_gene1 | 5 | 6.90E-38 | 47 |
| HBM_contig1494088_s582224c2_gene2 | 4 | 4.49E-28 | 47 |
| HBM_contig771203_s304239c15_gene1 | 3 | 5.61E-08 | 47 |
| HBM_contig1109905_s381969_gene1 | 7 | 2.84E-17 | 45 |

| | | | |
|---|---|---|---|
| HBM_contig1348390_s448422c26_gene1 | 3 | 4.27E-25 | 45 |
| HBM_contig331920_s146093c2_gene1 | 22 | 9.62E-30 | 45 |
| HBM_contig355008_s157135c8_gene1 | 4 | 9.30E-36 | 45 |
| HBM_contig1314791_s421584c5_gene1 | 12 | 2.24E-35 | 44 |
| HBM_contig1409221_s506095c5_gene3 | 2 | 5.04E-39 | 44 |
| HBM_contig164849_s72856c4_gene2 | 4 | 7.03E-20 | 44 |
| HBM_contig1409222_s506095c6_gene1 | 2 | 1.05E-33 | 43 |
| HBM_contig314926_s138259_gene1 | 12 | 1.29E-35 | 43 |
| HBM_contig1291168_s410297_gene2 | 9 | 1.43E-34 | 42 |
| HBM_contig446110_s205232c2_gene1 | 7 | 6.70E-08 | 42 |
| HBM_contig323185_s142103c31_gene1 | 4 | 1.69E-23 | 40 |
| HBM_contig1119082_s384118c3_gene1 | 1 | 4.23E-26 | 37 |
| HBM_contig1289127_s409874c9_gene1 | 1 | 1.50E-38 | 36 |
| HBM_contig1288955_s409848c4_gene1 | 2 | 7.10E-06 | 35 |
| HBM_contig1311113_s419277c3_gene1 | 6 | 5.30E-09 | 35 |
| HBM_contig403677_s182258c2_gene1 | 3 | 2.00E-07 | 34 |
| HBM_contig1289511_s409944c4_gene3 | 3 | 1.15E-07 | 33 |
| HBM_contig1318935_s424517c31_gene1 | 3 | 7.59E-18 | 32 |
| HBM_contig371632_s165417c5_gene1 | 4 | 5.42E-08 | 29 |
| HBM_contig1420163_s516850c9_gene1 | 1 | 1.88E-08 | 26 |
| HBM_contig353116_s156220_gene1 | 2 | 2.09E-07 | 26 |
| HBM_contig451554_s208132c13_gene1 | 1 | 9.18E-06 | 26 |

[a]TBLASTN hits with E-value < $10^{-5}$

[b]In cases where multiple hits occur, the reported value is for the top hit

**Table S18.** Lignocellulose biosynthetic genes of *H. brasiliensis* in comparison to other sequenced genomes

| | *Hevea* | *Ricinus* | *Populus* | *Arabidopsis* | *Oryza* | *Selaginella* |
|---|---|---|---|---|---|---|
| **Lignin synthesis related genes** | | | | | | |
| PAL | 4 | 4 | 5 | 4 | 6 | 1 |
| C4H | 7 | 1 | 2 | 1 | 2 | - |
| 4CL | 4 | 3 | 3 | 4 | 5 | 2 |
| HCT | 6 | 1 | 1 | 1 | 2 | 3 |
| COMT | 10 | 46 | 41 | 17 | 49 | 17 |
| C3H | 6 | 2 | 3 | 1 | 2 | - |
| CCoAOMT | 3 | 3 | 2 | 1 | 1 | 2 |
| CCR | 3 | | 3 | 2 | 3 | |
| F5H | 2 | 1 | 3 | 1 | 1 | - |
| CAD | 5 | 10 | 24 | 9 | 20 | 9 |
| **Cellulose synthesis related genes** | | | | | | |
| CesA | 36 | 13 | 28 | 10 | 20 | 6 |
| COBRA | 10 | 6 | 4 | 1 | 9 | 2 |
| SuSy | 8 | 5 | 7 | 6 | 5 | - |
| FRA2 | 4 | 1 | 2 | 1 | 1 | 1 |
| KOR | 7 | 3 | 4 | 3 | 3 | 1 |
| α-L-fucosidases | 12 | 23 | 38 | 25 | 67 | 45 |

**Table S19.** Putative NBS-coding R genes of *H. brasiliensis* in comparsion to other sequenced genomes

| Predicted Protein | *Hevea* | *Populus* | *Papaya* | *Arabidopsis* | *Oryza* |
|---|---|---|---|---|---|
| ***Without LRRs*** | | | | | |
| TIR-NBS | 17 | 10 | 2 | 21 | 3 |
| CC-NBS | 134 | 19 | 2 | 5 | 7 |
| NBS | 350 | 49 | 17 | 1 | 45 |
| ***With LRRs*** | | | | | |
| TIR-NBS-LRR | 25 | 64 | 6 | 93 | 0 |
| CC-NBS-LRR | 45 | 119 | 4 | 51 | 160 |
| NBS-LRR | 47 | 90 | 23 | 3 | 304 |
| **Total** | **618** | **351** | **54** | **174** | **519** |

**Table S20.** Pathogenesis-related proteins of *H. brasiliensis* in comparison with other genomes

| Pathogenesis-Related Proteins (PRs) | *Hevea* | *Ricinus* | *Populus* | *Arabidopsis* | *Oryza* | *Selaginella* |
|---|---|---|---|---|---|---|
| Pr1 | 12 | 7 | 16 | 10 | 10 | 3 |
| Pr2 | 22 | 61 | 125 | 95 | 96 | 60 |
| Pr3 | 7 | 9 | 23 | 14 | 19 | 23 |
| Pr4 | 27 | 3 | 8 | 1 | 5 | 1 |
| Pr5 | 17 | 26 | 42 | 22 | 40 | 18 |
| Pr6 | 5 | 4 | 20 | 6 | - | - |
| Pr8 | 14 | 8 | 14 | 1 | 31 | 8 |
| Pr10 | 11 | 38 | 34 | 26 | 2 | 5 |
| Lectins | 32 | 48 | 64 | 102 | 127 | 16 |
| **Total** | **147** | **204** | **346** | **277** | **330** | **134** |

**Table S21.** Systemic acquired resistance (SAR) and hypersensitive response (HR) related genes found in the *H. brasiliensis* genome

| Gene Model | Gene name |
|---|---|
| HBM_contig06421_s02656c2_gene2 | Metallothionein |
| HBM_contig1037845_s365794c13_gene3 | Non race-specific disease resistance 1 |
| HBM_contig105575_s45633c21_gene1 | F-box protein |
| HBM_contig1074434_s372022c6_gene1 | Serine-threonine protein kinase |
| HBM_contig117141_s50884c14_gene1 | Ethylene insensitive 3-like protein |
| HBM_contig1288682_s409802c24_gene1 | Bromodomain-containing protein |
| HBM_contig1288976_s409852c2_gene2 | Glycerol kinase |
| HBM_contig1289068_s409864c2_gene2 | WRKY-like transcription |
| HBM_contig1289631_s409964c4_gene2 | Alternative oxidase 2 |
| HBM_contig1290638_s410161c14_gene1 | Xanthine dehydrogenase 1 |
| HBM_contig1291402_s410361c4_gene2 | Serine/threonine protein kinase PSB1 |
| HBM_contig1291889_s410454c6_gene1 | Monoxygenase |
| HBM_contig1292364_s410573c2_gene1 | Oxalate oxidase |
| HBM_contig1292450_s410594c2_gene1 | Zinc finger protein |
| HBM_contig1292626_s410633c16_gene1 | Lipoxygenase |
| HBM_contig1295205_s411448c4_gene2 | Skp1-Cullin–F-box protein |
| HBM_contig1295495_s411532c6_gene1 | Protein 5'->3' exoribonuclease, AIN1 |
| HBM_contig1295989_s411738c1_gene1 | Peroxidase 17 |
| HBM_contig1296639_s411971c1_gene1 | Early ethylene-responsive genes |
| HBM_contig1296662_s411975c11_gene2 | Acyl-protein thioesterase |
| HBM_contig1296871_s412049c3_gene1 | Omega-3 fatty acid desaturase, FAD8 |
| HBM_contig1298013_s412552c3_gene1 | Ethylene response sensor 2 |
| HBM_contig1300802_s413756c18_gene1 | Mitogen-activated protein kinase kinase kinase 2 |
| HBM_contig1304427_s415555c19_gene4 | Isochorismate synthase 1 |
| HBM_contig1304763_s415719c13_gene1 | Omega-3 fatty acid desaturase |
| HBM_contig1307472_s417170c13_gene1 | Dimethylaniline monooxygenase |
| HBM_contig1308601_s417813c25_gene1 | Serine/threonine-protein kinase CTR1 |
| HBM_contig1315631_s422196c17_gene1 | Mitogen activated protein1 |
| HBM_contig1324775_s428843c2_gene1 | Allene oxide synthase |
| HBM_contig1325708_s429519c25_gene2 | Calmodulin-binding heat-shock protein, |
| HBM_contig1327523_s430947c6_gene1 | Regulatory protein NPR1 |
| HBM_contig1328657_s431840c3_gene3 | Wall-associated receptor kinase 2 |
| HBM_contig1333064_s435374c4_gene1 | Enhanced disease susceptibility 1 |
| HBM_contig1340690_s441737c6_gene1 | DNA binding protein GT-1 |
| HBM_contig1341105_s442084c7_gene1 | Beta glucosidase |
| HBM_contig1342092_s442954c9_gene2 | Phospholipase |
| HBM_contig1345388_s445788c3_gene1 | Zeaxanthin epoxidase (1.14.13.90) |
| HBM_contig1347991_s448099c5_gene1 | Hydroxynitrile lyase |
| HBM_contig1422110_s518739c4_gene1 | Serine/threonine protein kinase |
| HBM_contig1465004_s559611c14_gene2 | Pectate lyase |
| HBM_contig173710_s76922c5_gene1 | CCoAMT; caffeoyl-CoA O-methyltransferase |
| HBM_contig17421_s07081c9_gene1 | Heat shock protein |

| | |
|---|---|
| HBM_contig188767_s83719c8_gene1 | cytochrome P450 probable ent-kaurenoic acid oxidase |
| HBM_contig200851_s89231c10_gene1 | Receptor protein kinase |
| HBM_contig226693_s100838c4_gene1 | Serine/threonine phosphatase |
| HBM_contig228172_s101510c3_gene3 | Brassinosteroid insensitive 1 |
| HBM_contig238412_s106118_gene1 | Eukaryotic translation initiation factor 2c |
| HBM_contig242210_s107817c13_gene1 | Glutathione peroxidas |
| HBM_contig252939_s112426c6_gene2 | Zeamatin precursor |
| HBM_contig262953_s116732c1_gene1 | Mitogen-activated protein kinase 2 |
| HBM_contig263035_s116770c1_gene1 | Flavine-containing monoxygenase |
| HBM_contig264998_s117599_gene1 | Rhicadhesin receptor precursor |
| HBM_contig265562_s117837_gene1 | Coronatine-insensitive 1 |
| HBM_contig274137_s121393c1_gene1 | Leucine-rich repeat-containing protein |
| HBM_contig274142_s121393c6_gene1 | OUT domain protein |
| HBM_contig283262_s124944c9_gene1 | Brassinosteroid insensitive 1 associated receptor kinase 1 |
| HBM_contig285476_s125769c6_gene1 | Ethylene receptor 2 |
| HBM_contig296913_s130307_gene1 | Ethylene-responsive transcription factor |
| HBM_contig315927_s138736c1_gene1 | Pathogenesis-related protein 1 |
| HBM_contig322575_s141866c13_gene1 | Mitogen activated kinase-like protein |
| HBM_contig322635_s141887c15_gene2 | Sphingoid base Hydroxylase 1 |
| HBM_contig329111_s144804_gene1 | Nucleoporin |
| HBM_contig333187_s146668c1_gene1 | Disease resistance protein |
| HBM_contig339286_s149545c5_gene1 | Latex beta-cyanoalanine synthase |
| HBM_contig341044_s150417c9_gene4 | 2-nitropropane dioxygenase family |
| HBM_contig346636_s153048c2_gene1 | Map3k delta-1 protein kinase |
| HBM_contig349742_s154477c6_gene1 | Disease resistance protein 2 |
| HBM_contig351365_s155313c3_gene2 | Poly(rC)-binding protein |
| HBM_contig369959_s164591c6_gene1 | ethylene-responsive element bining factor 5 |
| HBM_contig39977_s16454c9_gene1 | Transcription factor HBP-1b |
| HBM_contig406858_s183928c16_gene1 | APX1 (ascorbate peroxidase 1) |
| HBM_contig406860_s183928c18_gene1 | Mitogen-activated protein kinase |
| HBM_contig409421_s185323_gene1 | WRKY transcription factor, putative |
| HBM_contig423248_s192871c2_gene2 | Calmodulin |
| HBM_contig423596_s193057c15_gene1 | Ubiquitin ligase |
| HBM_contig427107_s194975c3_gene1 | Mitogen-activated protein kinase kinase |
| HBM_contig442498_s203277c4_gene1 | Stearoyl-acyl-carrier protein desaturase |
| HBM_contig455602_s210290c11_gene1 | Ethylene receptor 1 |
| HBM_contig469575_s217788c5_gene2 | Phytoalexin deficient4 |
| HBM_contig483419_s224948c2_gene1 | Disease resistance protein 8 |
| HBM_contig494277_s230381c9_gene2 | GH3 family protein |
| HBM_contig509911_s237995c8_gene1 | Copine |
| HBM_contig523425_s244330_gene1 | Transcription factor MYC2 |
| HBM_contig536436_s250034c21_gene1 | Jasmonate-insensitive 3 |
| HBM_contig549096_s255049c1_gene2 | DNA-damage-inducible protein |
| HBM_contig644472_s277880c1_gene1 | Mitogen-activated protein kinase kinase kinase1 |
| HBM_contig701896_s287934c9_gene1 | Ein3-binding f-box protein 3 |
| HBM_contig732471_s294373c14_gene3 | Ethylene- insensitive 4 |

| | |
|---|---|
| HBM_contig73508_s31074c6_gene1 | Gamma-glutamylcysteine synthetase |
| HBM_contig735668_s295000c2_gene1 | Catalytic/ glucuronoxylan glucuronosyltransferase |
| HBM_contig764172_s302159c11_gene1 | Mitogen-activated protein kinase kinase 2 |
| HBM_contig764172_s302159c11_gene2 | protein phosphatase 2C |
| HBM_contig821365_s316780c31_gene1 | Ethylene-insensitive protein 2 |
| HBM_contig829332_s318669c4_gene1 | ABL Interactor-Like Protein 2 |
| HBM_contig863487_s324777c6_gene2 | S-phase kinase-associated protein 1 |
| HBM_contig958308_s346865c1_gene1 | Phospholipase d beta |

**Table S22.** Latex allergens in the *H. brasiliensis* genome

| Allergen type | Names and contig number |
|---|---|
| **Hevb 1** | **Rubber elongation factor (REF)** |
| | HBM_contig1314787_s421584c1_gene1 |
| | HBM_contig1314789_s421584c3_gene1 |
| | HBM_contig1352860_s452368_gene1 |
| | HBM_contig1432969_s529413_gene1 |
| | HBM_contig210302_s93430_gene1 |
| | HBM_contig246314_s109563_gene1 |
| | HBM_contig285004_s125605_gene1 |
| | HBM_contig289068_s127107_gene1 |
| | HBM_contig331924_s146093c6_gene1 |
| | HBM_contig331929_s146093c11_gene1 |
| | HBM_contig420863_s191570c9_gene1 |
| | HBM_contig953976_gene1 |
| **Hevb 2** | **β-1,3-glucanase** |
| | HBM_contig1053939_gene1 |
| | HBM_contig1288699_s409805c4_gene6 |
| | HBM_contig1288700_s409805c5_gene2 |
| | HBM_contig1331601_s434259_gene1 |
| | HBM_contig1383052_s480661_gene1 |
| | HBM_contig1383052_s480661_gene2 |
| | HBM_contig1493847_gene1 |
| | HBM_contig1542420_gene1 |
| | HBM_contig181937_s80601_gene1 |
| | HBM_contig484011_s225263c1_gene1 |
| | HBM_contig49288_s20505_gene1 |
| **Hevb 3** | **Small rubber particle** |
| | HBM_contig1291168_s410297_gene2 |
| | HBM_contig1314791_s421584c5_gene1 |
| | HBM_contig1351062_s450759c14_gene1 |
| | HBM_contig1445641_s541695c60_gene1 |
| | HBM_contig878396_gene1 |
| | HBM_contig364556_s161856_gene1 |
| | HBM_contig331921_s146093c3_gene1 |
| | HBM_contig331923_s146093c5_gene1 |
| | HBM_contig314926_s138259_gene1 |
| | HBM_contig331920_s146093c2_gene1 |
| **Hevb 4** | **Lecithinase homolog** |
| | HBM_contig367975_s163585_gene1 |
| | HBM_contig1311038_s419233c1_gene1 |
| | HBM_contig1302448_s414608_gene1 |
| | HBM_contig128030_s55849c2_gene1 |
| | HBM_contig128029_s55849c1_gene1 |

| | |
|---|---|
| **Hevb 5** | **Latex major allergen** |
| | HBM_contig279320_s123416c2_gene1 |
| **Hevb 6** | **Hevein** |
| | HBM_contig1099636_s379205_gene1 |
| | HBM_contig1326398_s430072_gene1 |
| | HBM_contig1332655_s435061c1_gene1 |
| | HBM_contig1404480_s501478_gene1 |
| | HBM_contig1438942_s535269_gene1 |
| | HBM_contig1463769_s558530_gene1 |
| | HBM_contig1485741_s576443_gene1 |
| | HBM_contig1487445_s577627_gene1 |
| | HBM_contig1511743_gene1 |
| | HBM_contig1523411_s597290_gene1 |
| | HBM_contig1544593_s603451_gene1 |
| | HBM_contig1551202_s604715_gene1 |
| | HBM_contig1580472_gene1 |
| | HBM_contig293053_s128596c1_gene1 |
| | HBM_contig36291_gene1coding |
| | HBM_contig795181_s310723_gene1 |
| **Hevb 7** | **Patatin-like** |
| | HBM_contig274460_s121513c2_gene1 |
| | HBM_contig269214_s119330c6_gene1 |
| | HBM_contig604530_s271122c2_gene1 |
| | HBM_contig1301350_s414027c3_gene2 |
| | HBM_contig1301351_s414027c4_gene1 |
| **Hevb 8** | **Profilin** |
| | HBM_contig415131_s188411_gene1 |
| | HBM_contig1330838_s433634_gene1 |
| | HBM_contig367050_s163105c2_gene1 |
| | HBM_contig1164698_s393417c31_gene3 |
| | HBM_contig1074430_s372022c2_gene1 |
| | HBM_contig1329259_s432305_gene1 |
| **Hevb 9** | **Enolase** |
| | HBM_contig260697_s115748c12_gene |
| | HBM_contig295239_s129579c5_gene1 |
| | HBM_contig319636_s140387c5_gene1 |
| | HBM_contig383573_s171660c8_gene1 |
| **Hevb 10** | **Superoxide dismutase (Mn)** |
| | HBM_contig1380717_s478448c12_gene1 |
| | HBM_contig1299900_s413337c4_gene3 |
| **Hevb 11** | **Chitinase** |
| | HBM_contig1331506_s434174c2_gene1 |
| | HBM_contig1374580_s472577_gene1 |
| | HBM_contig1434962_s531376_gene1 |
| | HBM_contig376395_s167879c1_gene1 |
| | HBM_contig380563_s170073_gene1 |

| | |
|---|---|
| | HBM_contig412336_s186887c8_gene1 |
| | HBM_contig594755_gene1 |
| | HBM_contig782692_s307260_gene1 |
| | HBM_contig951921_s344934c1_gene1 |
| | HBM_contig952555_gene1 |
| | HBM_contig980709_s353149_gene1 |
| **Hevb 12** | **Lipid transfer precursor** |
| | HBM_contig323161_s142103c7_gene3 |
| | HBM_contig349554_s154385c1_gene1 |
| **Hevb 13** | **Esterase** |
| | HBM_contig371165_s165162c13_gene1 |
| | HBM_contig371163_s165162c11_gene3 |
| | HBM_contig1317196_s423303_gene1 |
| | HBM_contig134028_s58638_gene1 |
| | HBM_contig899653_s333225_gene1 |
| | HBM_contig371167_s165162c15_gene1 |
| | HBM_contig370747_s164953c24_gene1 |
| | HBM_contig371169_s165162c17_gene1 |
| | HBM_contig464308_s214932_gene1 |
| **Hevb 14** | **Hevamine** |
| | HBM_contig1389101_s486528c4_gene1 |
| | HBM_contig1371073_s469275_gene1 |
| | HBM_contig1344950_s445443c5_gene6 |
| | HBM_contig1321967_s426707c2_gene2 |
| | HBM_contig1310281_s418802c6_gene1 |
| | HBM_contig1303495_s415132_gene1 |

**Table S23.** Non-latex allergens in the *H. brasiliensis* genome

| Non-latex allergen | Gene model |
|---|---|
| Pollen allergen | HBM_contig623408_s274630c9_gene1 |
| | HBM_contig1074430_s372022c2_gene2 |
| α-expansin | HBM_contig289660_s127337c2_gene1 |
| | HBM_contig311528_s136675c1_gene1 |
| | HBM_contig1291975_s410466c42_gene1 |
| | HBM_contig1369889_s468147_gene1 |
| | HBM_contig302755_s132887_gene1 |
| | HBM_contig350467_s154865_gene1 |
| | HBM_contig350403_s154837_gene1 |
| | HBM_contig446231_s205297_gene1 |
| β-expansin | HBM_contig903040_s334087c9_gene1 |
| | HBM_contig818284_s315950c3_gene1 |
| Isoflavone reductase | HBM_contig334692_s147398c6_gene1 |
| | HBM_contig252987_s112426c54_gene1 |
| | HBM_contig1305222_s415946c2_gene1 |
| | HBM_contig212170_s94313_gene1 |

**Table S24.** Transcription factors present in *H. brasiliensis* in comparison to other sequenced plant genomes

| **Transcription Factors** | ***Hevea*** | ***Ricinus*** | ***Populus*** | ***Arabidopsis*** | ***Oryza*** | ***Selaginella*** |
|---|---|---|---|---|---|---|
| bHLH | 752 | 121 | 226 | 194 | 178 | 85 |
| MYB | 570 | 63 | 212 | 159 | 124 | 41 |
| C3H | 470 | 36 | 69 | 56 | 50 | 36 |
| G2-like | 461 | 33 | 74 | 55 | 56 | 32 |
| WRKY | 445 | 60 | 119 | 89 | 116 | 25 |
| MYB_related | 397 | 99 | 104 | 85 | 94 | 63 |
| NAC | 336 | 97 | 182 | 135 | 166 | 38 |
| ERF | 246 | 94 | 176 | 132 | 143 | 68 |
| HD-ZIP | 182 | 31 | 67 | 56 | 46 | 14 |
| C2H2 | 164 | 69 | 115 | 104 | 91 | 44 |
| AP2 | 139 | 18 | 30 | 25 | 27 | 16 |
| TALE | 139 | 16 | 35 | 23 | 28 | 5 |
| HSF | 136 | 19 | 32 | 27 | 44 | 14 |
| B3 | 122 | 41 | 116 | 71 | 55 | 24 |
| ARR-B | 116 | 11 | 21 | 16 | 9 | 9 |
| MADS-box | 112 | 50 | 116 | 156 | 108 | 20 |
| NF-YB | 106 | 14 | 22 | 19 | 14 | 9 |
| GATA | 97 | 19 | 46 | 31 | 32 | 11 |
| GRAS | 96 | 48 | 114 | 36 | 77 | 89 |
| SBP | 88 | 15 | 32 | 18 | 20 | 16 |
| WOX | 78 | 11 | 20 | 17 | 12 | 13 |
| LBD | 63 | 34 | 59 | 43 | 42 | 21 |
| CO-like | 62 | 9 | 16 | 19 | 11 | 7 |
| ZF-HD | 62 | 11 | 24 | 17 | 15 | 15 |
| YABBY | 58 | 6 | 13 | 8 | 15 | 1 |
| ARF | 56 | 17 | 53 | 35 | 34 | 29 |
| NF-YC | 52 | 11 | 16 | 15 | 15 | 7 |
| TCP | 38 | 22 | 35 | 32 | 27 | 9 |
| GRF | 37 | 14 | 19 | 10 | 22 | 6 |
| HB-other | 37 | 10 | 15 | 8 | 15 | 15 |
| CAMTA | 34 | 5 | 9 | 8 | 8 | 12 |
| E2F/DP | 25 | 6 | 10 | 12 | 9 | 7 |
| Nin-like | 25 | 10 | 49 | 20 | 19 | 22 |
| RAV | 25 | 4 | 4 | 6 | 4 | 3 |
| Trihelix | 24 | 34 | 60 | 33 | 32 | 45 |
| DBB | 23 | 7 | 16 | 12 | 11 | 6 |
| BES1 | 16 | 5 | 14 | 11 | 5 | 10 |
| HB-PHD | 15 | 2 | 4 | 2 | 1 | 2 |
| Whirly | 11 | 2 | 4 | 4 | 2 | 2 |
| CPP | 10 | 6 | 19 | 10 | 20 | 8 |
| FAR1 | 9 | 21 | 54 | 20 | 71 | 0 |
| GeBP | 9 | 6 | 11 | 29 | 24 | 4 |

| | | | | | | |
|---|---|---|---|---|---|---|
| BBR/BPC | 7 | 5 | 16 | 17 | 7 | 2 |
| SAP | 7 | 1 | 1 | 1 | 0 | 2 |
| EIL | 6 | 4 | 8 | 6 | 12 | 10 |
| Dof | 5 | 25 | 48 | 45 | 39 | 41 |
| LFY | 3 | 1 | 1 | 1 | 2 | 4 |
| bZIP | 2 | 51 | 106 | 101 | 99 | 39 |
| NZZ/SPL | 2 | 0 | 2 | 1 | 0 | 2 |
| S1Fa-like | 2 | 1 | 2 | 4 | 3 | 0 |
| NF-X1 | 1 | 2 | 5 | 2 | 3 | 4 |
| **Total** | **5978** | **1297** | **2621** | **2036** | **2057** | **1007** |

**Table S25.** Genes involved in phytohormone metabolism, signaling and regulatory events represented in the *H. brasiliensis* genome

| **Biological Processes** | ***Hevea*** | ***Ricinus*** | ***Populus*** | ***Arabidopsis*** | ***Oryza*** | ***Selaginella*** |
|---|---|---|---|---|---|---|
| **Auxin Homeostasis And Signaling** | | | | | | |
| TIR1/AFB auxin receptor protein PintaTIR1 | 11 | 3 | 6 | 4 | 3 | 1 |
| Auxin response factors | 20 | 19 | 43 | 25 | 33 | 14 |
| Aux/IAA repressors | 6 | 18 | 35 | 29 | 35 | 4 |
| Auxin binding proteins | 6 | 1 | 1 | 1 | 2 | 1 |
| PIN auxin efflux carriers | 16 | 7 | 18 | 7 | 6 | 4 |
| AUX1/LAX auxin influx transporters | 12 | 4 | 8 | 4 | 5 | 3 |
| YUCCA/FLOOZY monoxygenases | 13 | 4 | 5 | 7 | 2 | 2 |
| Class II GH3 IAA amidosynthetases | 11 | 5 | 9 | 17 | 8 | 1 |
| IRL1/ILL IAA amidohydrolases | 18 | 7 | 12 | 7 | 11 | 2 |
| Small Auxin-Up RNA (SAUR) | 21 | 9 | 99 | 81 | 56 | 28 |
| Topless/Topless Related | 17 | 6 | 11 | 5 | 3 | 2 |
| TAA/TAR (Tryptophan Aminotransferase-/Related) | 6 | 1 | 1 | 1 | - | - |
| Total | *157* | *84* | *248* | *188* | *164* | *62* |
| **Cytokinin Biosynthetic Pathway** | | | | | | |
| Adenylate Isopentenyltransferase/ Adenosine Phosphate-Isopentenyltransferases (Ipts) | 8 | 5 | 8 | 8 | 10 | - |
| Trans-Zeatin Riboside Monophosphate Phosphoribohydrolase (LOG) | 21 | 34 | 31 | 20 | 32 | 4 |
| Adenosine Kinase (AK) | 11 | 1 | 2 | 2 | 2 | 1 |
| Adenine Phosphoribosyltransferase (APRT) | 10 | 4 | 4 | 5 | 6 | 1 |
| CKX/ Cytokinin Oxidase/ Dehydrogenase | 13 | 6 | 9 | 7 | 11 | 1 |
| Zeatin O-Glucosyltransferase | 37 | 70 | 149 | 60 | 148 | 15 |
| Total | *100* | *120* | *203* | *102* | *209* | *22* |
| **Gibberellin Biosynthetic Pathway** | | | | | | |
| CYP88A | 8 | 1 | 2 | 2 | 1 | 2 |
| GA20ox (17) | 5 | 1 | 2 | 2 | 1 | - |
| GA3ox | 1 | 2 | - | 1 | - | - |
| GA2ox | 9 | 5 | 4 | 2 | 2 | - |
| GA7ox | 1 | - | - | 1 | - | - |
| GID | 4 | 36 | 67 | 20 | 70 | 18 |
| DELLA | 5 | 2 | 4 | 5 | 1 | 1 |
| GASA | 7 | 14 | 21 | 15 | 6 | 6 |
| SPINDLY | 1 | 1 | 2 | 1 | 1 | - |
| Total | *41* | *62* | *102* | *49* | *82* | *27* |
| **Ethylene Biosynthesis** | | | | | | |
| s-adenosylmethionine synthetase | 9 | 5 | 9 | 7 | 10 | 5 |
| 1-aminocyclopropane-1-carboxylate deaminase | 3 | 1 | 1 | 1 | 1 | 1 |
| 1ACC synthase | 5 | 7 | 12 | 13 | 6 | 4 |
| 1-aminocyclopropane-1-carboxylate oxidase | 13 | 8 | 17 | 22 | 12 | 4 |
| Ethylene Receptor | 6 | 16 | 13 | 6 | 13 | 7 |
| constitutive triple response-1 | 11 | 2 | 3 | 1 | 2 | 1 |
| ethylene insensitive 2 | 5 | 1 | 2 | 1 | 2 | 2 |
| ethylene responsive factor (ERF) | 246 | 92 | 172 | 117 | 121 | 39 |
| Ethylene overproducer 1 (ETO1) | 4 | 3 | 8 | 4 | 5 | 4 |
| Total | *302* | *135* | *237* | *172* | *172* | *67* |

| | | | | | | |
|---|---|---|---|---|---|---|
| **Jasmonic Acid Metabolic Pathway** | | | | | | |
| Lox (Lipoxygenase) | 10 | 14 | 26 | 6 | 24 | 34 |
| Aos (Allene oxide synthase) | 4 | 2 | 9 | 2 | 5 | 17 |
| Aoc (allene oxide cyclase) | 5 | 2 | 3 | 4 | 1 | 2 |
| Opr7 | 13 | 10 | 12 | 10 | 11 | 14 |
| Total | *32* | *28* | *50* | *22* | *41* | *67* |
| **Brassinosteroid Signaling** | | | | | | |
| DET2- probable steroid reductase | 2 | 1 | 1 | 1 | 2 | 1 |
| CYP90B1, DWF4 | 4 | 1 | 2 | 1 | 1 | - |
| CYP90A1, CPD | 7 | 1 | 2 | 1 | 2 | 1 |
| CYP90C1, ROT3 | 8 | 1 | 1 | 1 | - | - |
| CYP85A1, BR6OX1 | 4 | 1 | 3 | 2 | 1 | - |
| CYP90D1, D1 | 3 | 1 | 2 | 1 | 2 | 2 |
| CYP85A2 | 7 | 1 | 3 | 2 | 1 | - |
| Total | *35* | *7* | *14* | *9* | *9* | *4* |
| **Salicylic Acid Associated Genes** | | | | | | |
| PAL | 5 | 7 | 6 | 4 | 13 | 2 |
| PBS3 | 2 | 1 | 2 | 3 | 2 | 2 |
| 3-phosphoshikimate 1-carboxyvinyltransferase (EPSP synthase) | 3 | 2 | 3 | 3 | 1 | 4 |
| Total | *10* | *10* | *11* | *10* | *16* | *8* |
| **Nitric Oxide Related Genes** | | | | | | |
| Nos | 4 | 1 | 1 | 1 | 1 | 2 |
| SOD | 9 | 8 | 16 | 18 | 14 | 14 |
| Cat (Catalase) | 7 | 2 | 4 | 7 | 6 | 10 |
| Total | *20* | *11* | *21* | *26* | *21* | *26* |

**Table S26.** Circadian clock and light signaling gene families from *Hevea* in comparison to *Populus* and *Arabidopsis*

| **Gene name** | ***Hevea*** | ***Populus*** | ***Arabidopsis*** |
|---|---|---|---|
| PRR/CCT | 11 | 8 | 7 |
| ZTL/FKF1 | 10 | 5 | 3 |
| CCA1/LHY | 11 | 4 | 3 |
| RVE/ERP1 | 8 | 3 | 5 |
| PHOT | 13 | 3 | 2 |
| ELF3 | 7 | 2 | 1 |
| GI (Gigantea) | 5 | 2 | 1 |
| SRR1 | 24 | 2 | 2 |
| ELF4 | 2 | 5 | 4 |
| TIC/TKL | 4 | 2 | 1 |
| TEJ | 2 | 2 | 1 |
| PHY | 7 | 5 | 5 |
| CRY/cryptochrome/photolyase | 9 | 5 | 3 |
| PIF/PIL | 8 | 5 | 6 |
| Casein kinase | 18 | 15 | 16 |
| COP | 2 | 1 | 1 |
| SPA | 4 | 2 | 2 |
| HY5/HYH | 5 | 2 | 1 |
| DET | 2 | 2 | 1 |
| FUSCA | 2 | 2 | 1 |
| **Total** | **154** | **77** | **66** |

**Table S27.** Major genes involved in carotenoid biosynthesis in *H. brasiliensis* and *A. thaliana*

| **Gene Name** | **Gene Description** | ***Hevea*** | ***Arabidopsis*** |
|---|---|---|---|
| GGPPS | Geranylgeranyl diphosphate synthase | 6 | 13 |
| IDI | Isopentenyl diphosphate isomerase | 3 | 2 |
| PSY | Phytoene synthase | 5 | 1 |
| PDS | Phytoene desaturase | 9 | 1 |
| ZDS | Zeta-carotene desaturase | 2 | 1 |
| Crtlso | Carotene isomerase | 2 | 1 |
| LCYb | Lycopene β-cyclase | 2 | 1 |
| LCYe | Lycopene epsilon cyclase | 3 | 1 |
| CHYeP450 | Carotenoid epsilon ring 3-hydroxylase of the cytochrome P450 type | 2 | 1 |
| CHYbP450 | Carotene beta-ring hydroxylase/ oxygen binding | 3 | 1 |
| CHYb | Carotenoid beta-ring 3-hydroxylase | 5 | 2 |
| ZEP | Zeaxanthin epoxidase | 2 | 1 |
| VDE | Violaxanthin de-epoxidase | 2 | 1 |
| NSY | Neoxanthin synthase | 2 | 1 |
| **Total** | | **48** | **28** |
