# Supplementary material for: Draft genome sequence of the rubber tree Hevea brasiliensis
Source: BMC Genomics. 2013 Feb 2;14:75. doi: 10.1186/1471-2164-14-75 (PMC3575267; doi:10.1186/1471-2164-14-75)
Supplement: Additional file 2: Figure S1 — Linkage map of H. brasiliensis showing 18 linkage groups with 143 anchored scaffolds corresponding to the reported 154 microsatellite markers. Figure S2. Complete network of rubber biosynthesis in H. brasiliensis. Figure S3. Lignin biosynthesis. Figure S4. Systemic acquired resistance pathway. Figure S5. Hypersensitive response. Figure S6. Auxin biosynthesis. Figure S7. Auxin signaling pathway. Figure S8. Zeatin biosynthesis. Figure S9. Cytokinin signaling pathway. Figure S10. Gibberellin biosynthesis. Figure S11. Gibberellin signaling pathway. Figure S12. Ethylene biosynthesis. Figure S13. Ethylene signaling pathway. Figure S14. Brassinosteroid biosynthesis. Figure S15. Brassinosteroid signaling pathway. Figure S16. Jasmonic acid biosynthesis. Figure S17. Salicylic acid biosynthesis. Figure S18. Carotenoid biosynthesis. [file 1471-2164-14-75-S2.pdf]

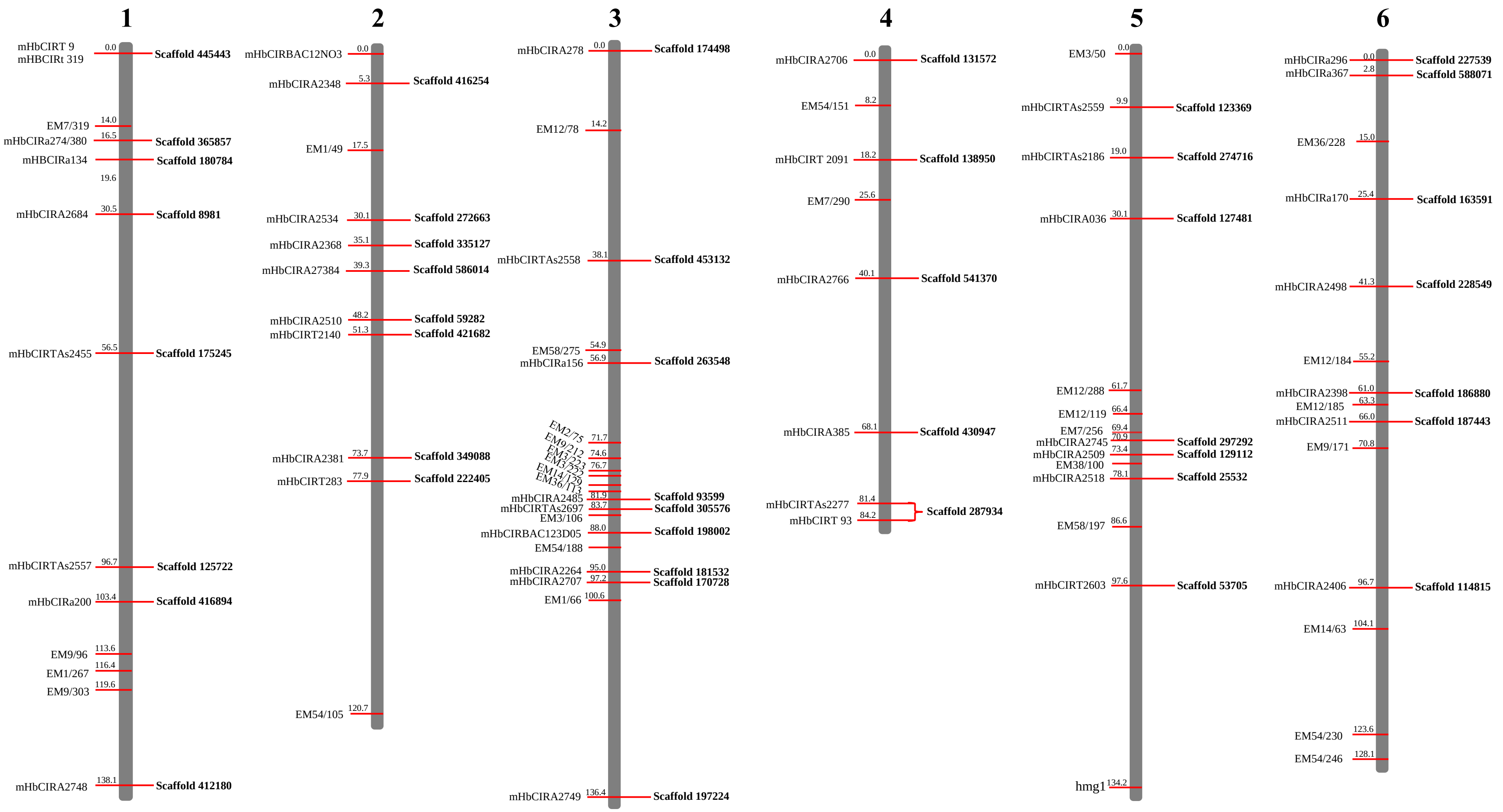

Figure S1. Linkage map of *H. brasiliensis* showing 18 linkage groups with 143 anchored scaffolds corresponding to the reported 154 microsatellite markers [17].

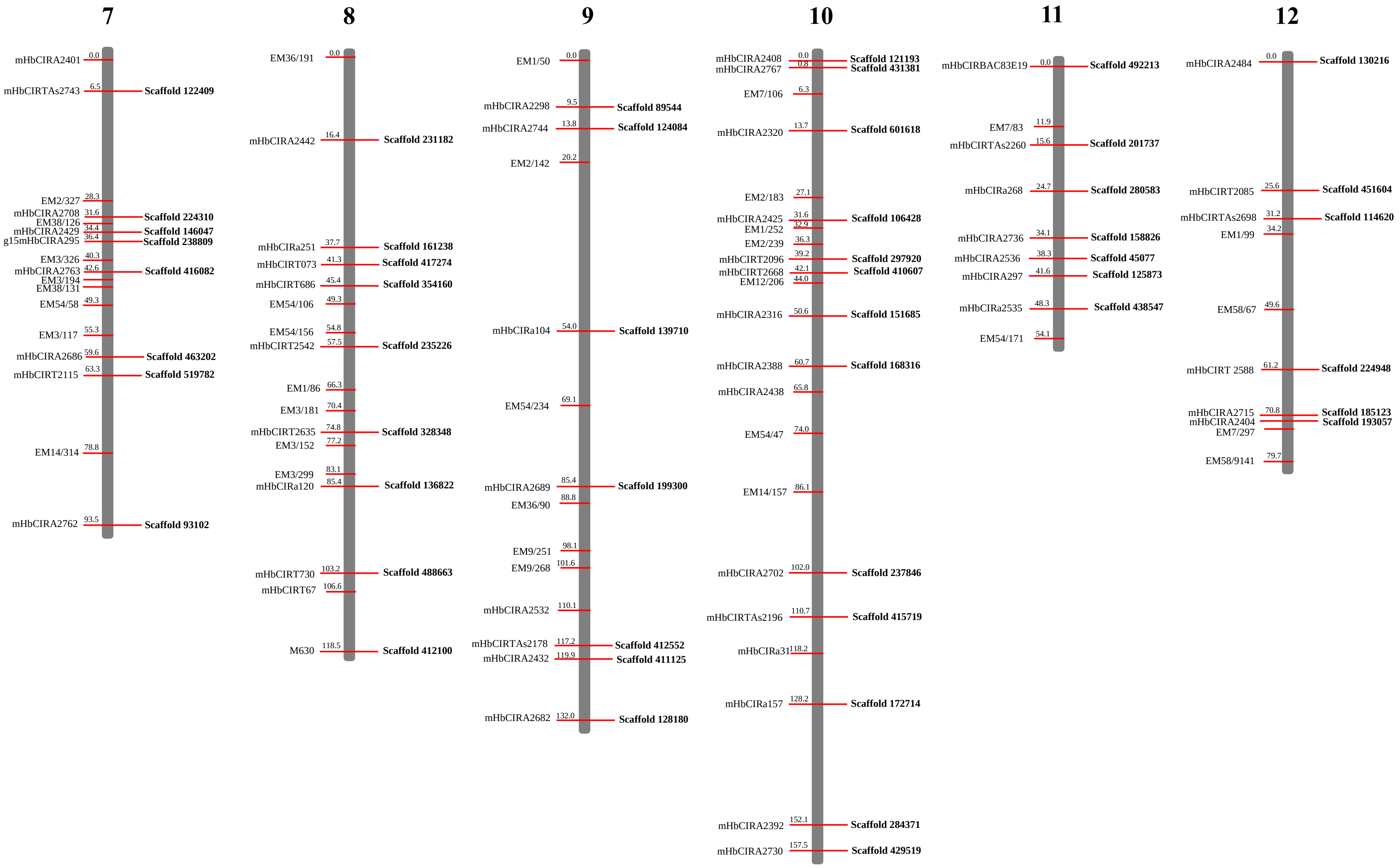

Figure S1. Continued.

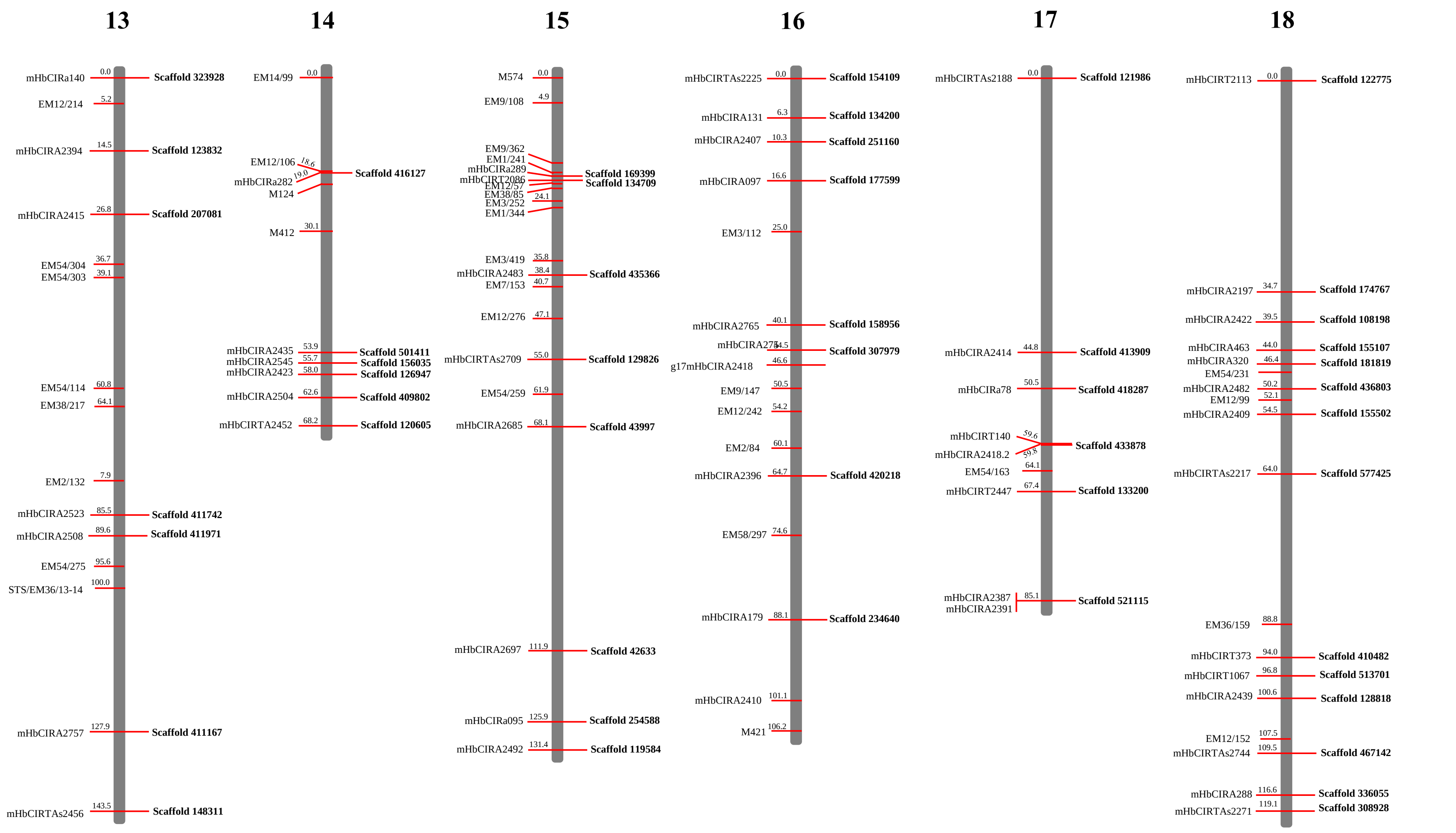

Figure S1. Continued.

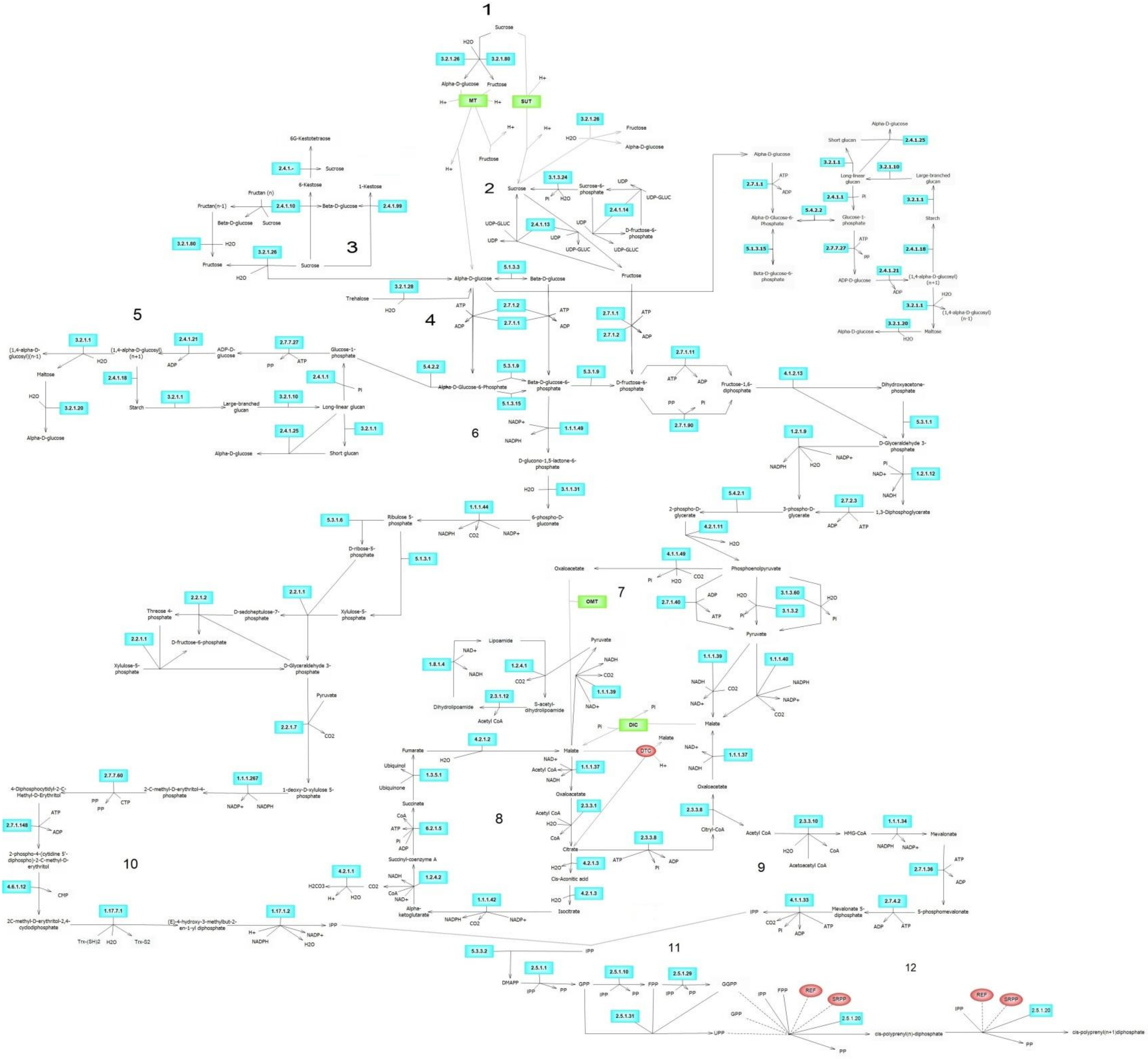

Figure S2. Complete network of rubber biosynthesis in *H. brasiliensis.* Complete metabolic pathways were reconstructed by Pathway Studio using Resnet-Plant 3.0 and metabolic pathway databases. It comprises 12 distinct sub-metabolic pathways: 1) sucrose import, 2) sucrose degradation, 3) fructan synthesis, 4) glycolysis, 5) starch metabolism, 6) pentose phosphate pathway, 7) acetyl CoA biosynthesis, 8) TCA cycle, 9) prenyl diphosphate synthesis via mevalonate (MVA) pathway, 10) 2-C-methyl-D-erythritol-4-phosphate (MEP) pathway, 11) prenyl-PP biosynthesis, and 12) rubber polymerization. DIC, Mitochondrial dicarboxylate carrier protein; DTC, Dicarboxylate/Tricarboxylate carrier protein; MT, monosaccharide transporter; SUT, sucrose transporter; OMT, oxoglutarate:malate antiporter; REF, rubber elongation factor; SRPP, small rubber particle protein.

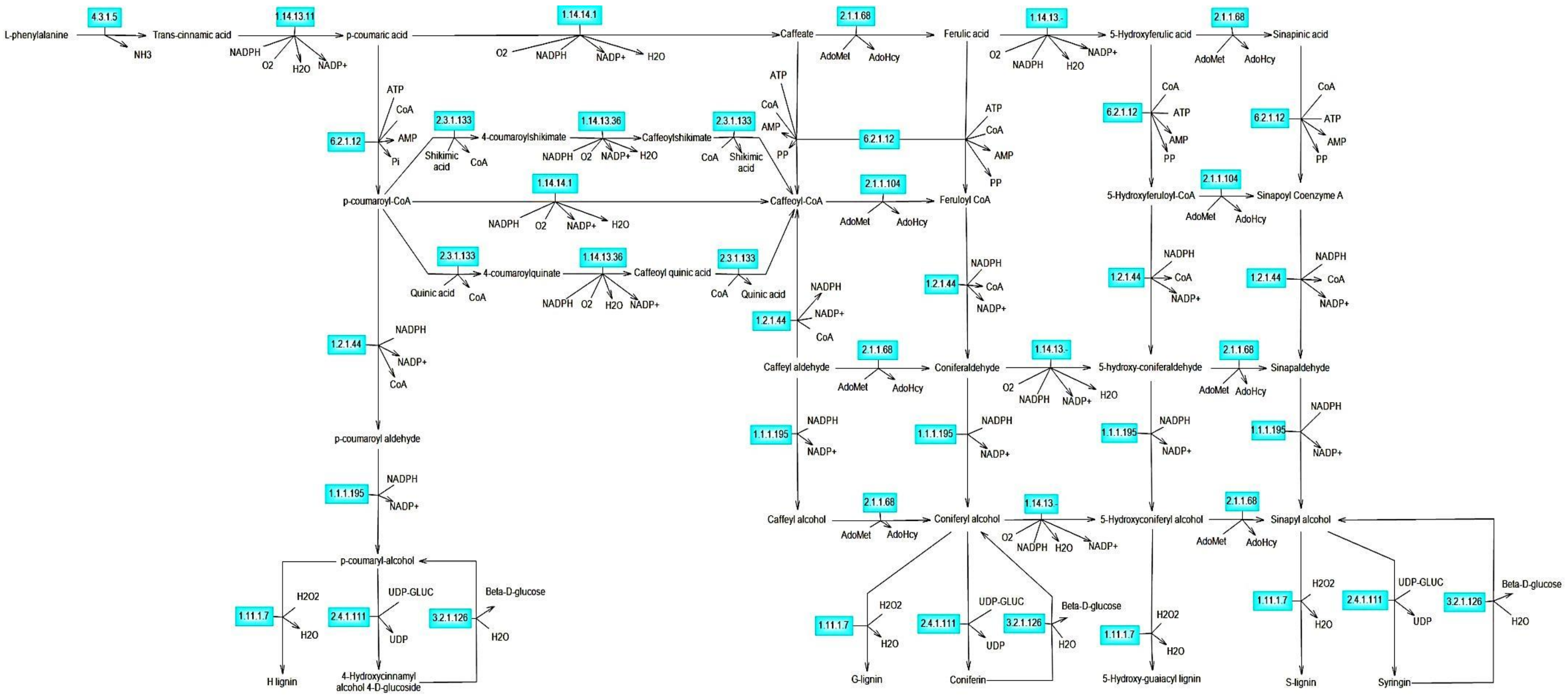

Figure S3. Lignin biosynthesis. Enzymes involved in the reaction are as listed: 6.2.1.12, 4-Coumarate-CoA ligase; 1.14.13.36, 5-O-(4-coumaroyl)-D-quinate 3'-monooxygenase; 2.1.1.68, caffeate O-methyltransferase; 2.4.1.111, coniferyl-alcohol glucosyltransferase; 1.14.13.11, *trans*-cinnamate 4-monooxygenase; 3.2.1.126, coniferin β-glucosidase; 1.1.1.195, cinnamyl-alcohol dehydrogenase; 2.1.1.104, caffeoyl-CoA O-methyltransferase; 4.3.1.5, phenylalanine ammonia-lyase; 1.2.1.44, cinnamoyl-CoA reductase; 1.11.1.7, peroxidase; 2.3.1.133, shikimate O-hydroxycinnamoyltransferase; 1.14.14.1, unspecific monooxygenase.

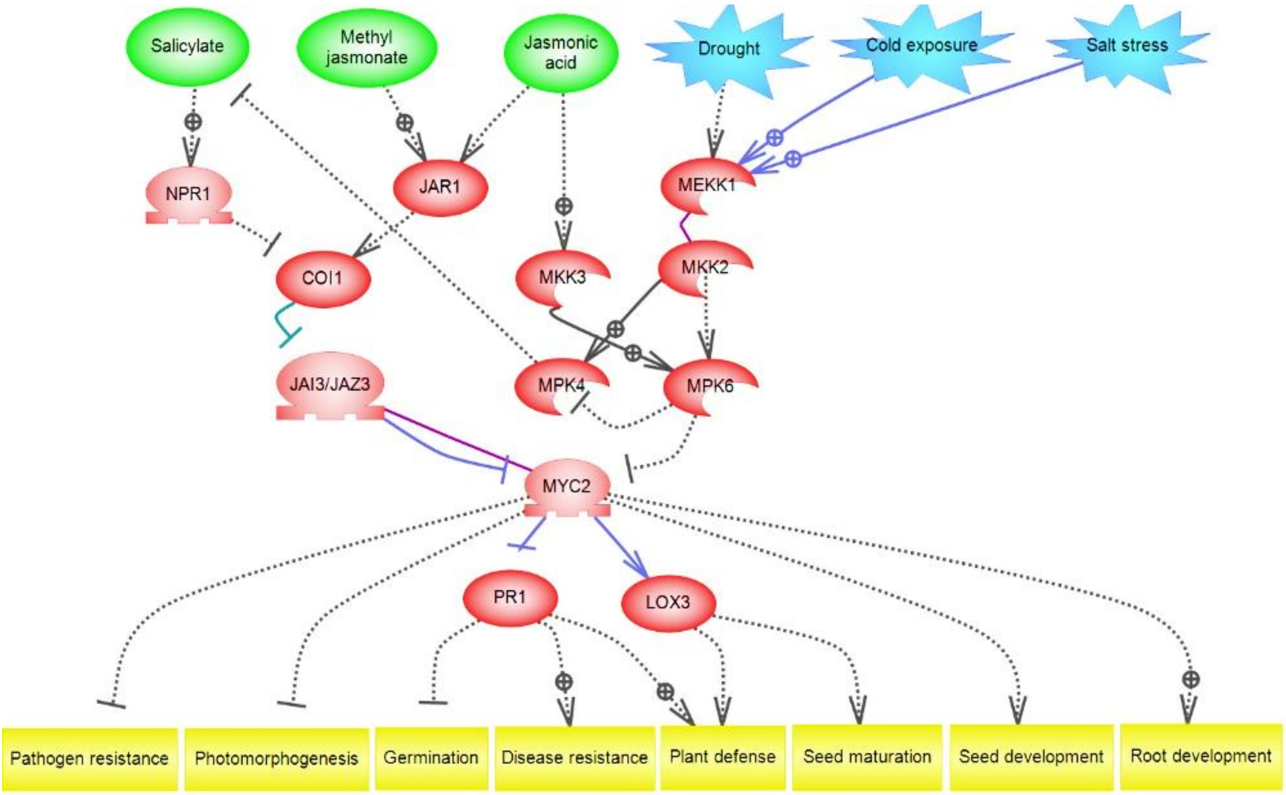

Figure S4. Systemic acquired resistance pathway. This pathway is responsive towards the external stimuli such as drought, cold exposure and salt stress as well as in the presence of salicylate, methyl jasmonate and jasmonic acid. The main regulator of this pathway is MYC2 which regulates the expression of PR1 and LOX3 and plays a major role in the biological process of rubber tree.

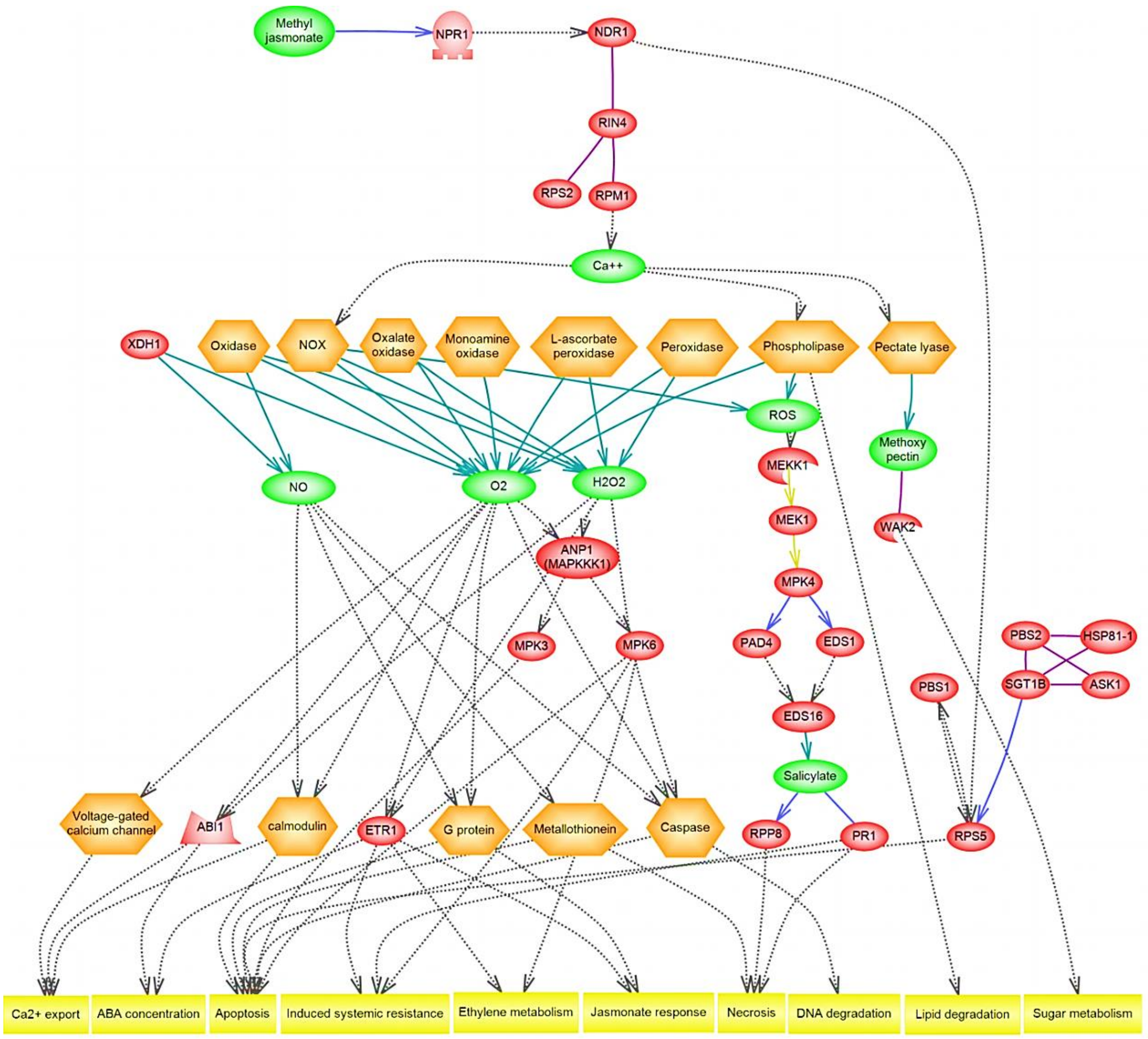

Figure S5. Hypersensitive response. This pathway shows the cross-talk that takes place when the plant is responsive towards the external factors including the small molecules and hormones. Subsequently, the signals lead to the downstream responses in the plant system.

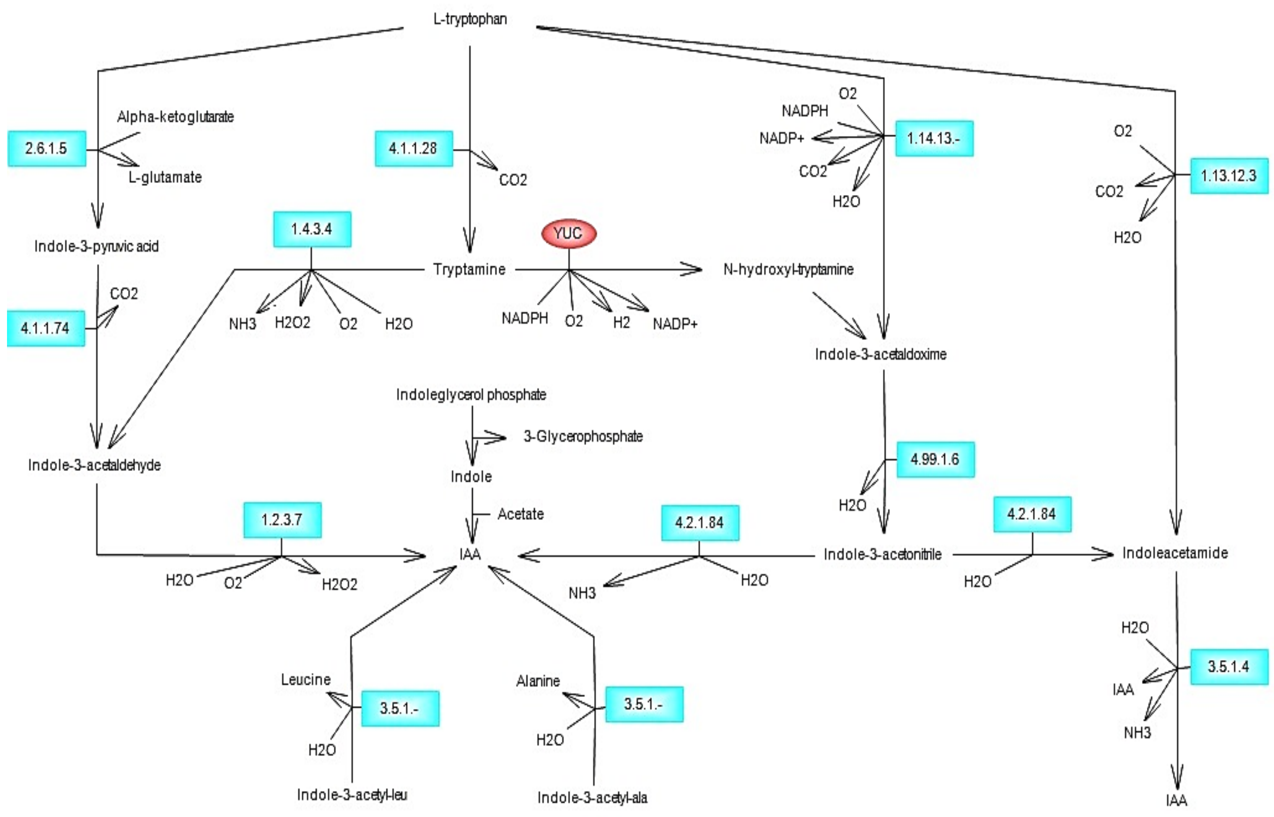

Figure S6. Auxin biosynthesis. Enzymes involved in the reaction are as listed: 1.14.13.-, β-carotene hydroxylase; 1.13.12.3, tryptophan 2-monooxygenase; 3.5.1.4, amidase; 4.2.1.84, nitrile hydratase; 1.2.3.7, indole-3-acetaldehyde oxidase; 1.4.3.4, monoamine oxidase; 4.99.1.6, indoleacetaldoxime dehydratase; 4.1.1.28, aromatic-L-amino-acid decarboxylase; 2.6.1.5, tyrosine transaminase; 4.1.1.74, indolepyruvate decarboxylase; 3.5.1.-, hydrolase.

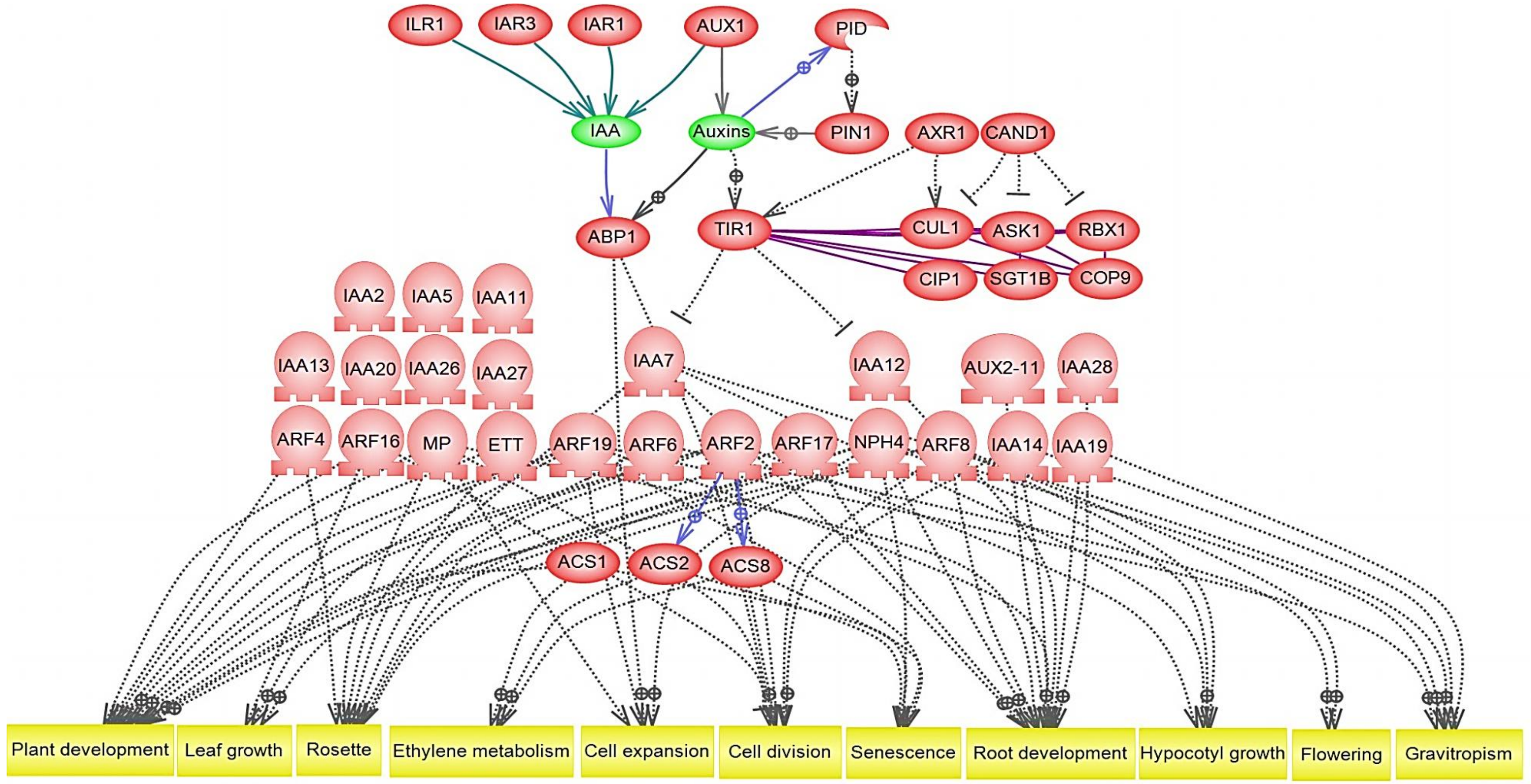

Figure S7. Auxin signaling pathway. The main regulators of this pathway are IAA7 and IAA12. Both of the transcriptional factors are regulated by TIR1 which is responsive towards the presence of auxins.

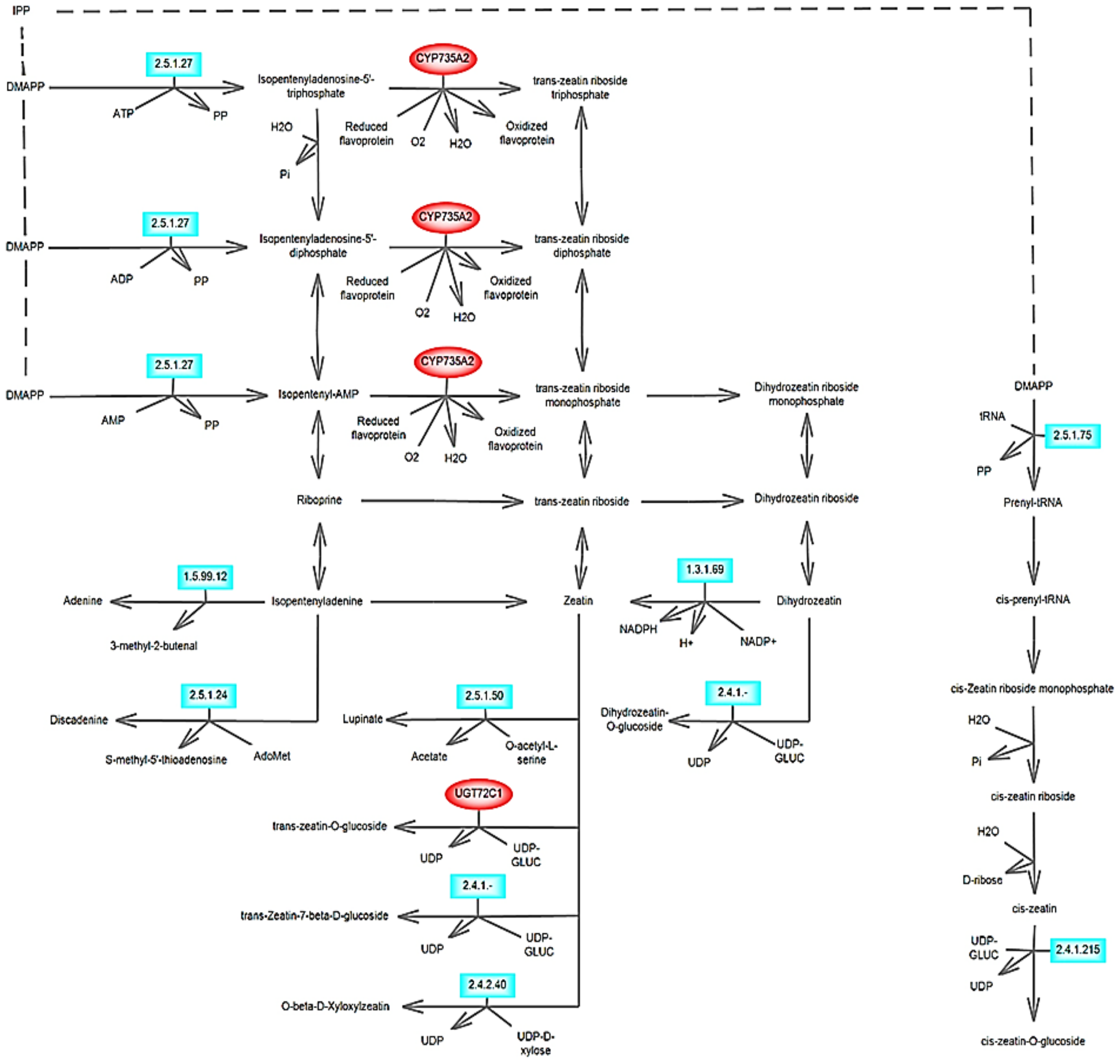

Figure S8. Zeatin biosynthesis. Enzymes involved in the reaction are as listed: 2.4.1.-, galtase; 1.5.99.12, cytokinin dehydrogenase; 2.4.1.215, *cis*-zeatin O-β-D-glucosyltransferase; 2.5.1.24, discadenin synthase; 1.3.1.69, zeatin reductase; 2.5.1.75, tRNA dimethylallyltransferase; 2.5.1.50, zeatin 9-aminocarboxyethyltransferase; 2.5.1.27, adenylate dimethylallyltransferase; 2.4.2.40, zeatin O-β-D-xylosyltransferase.

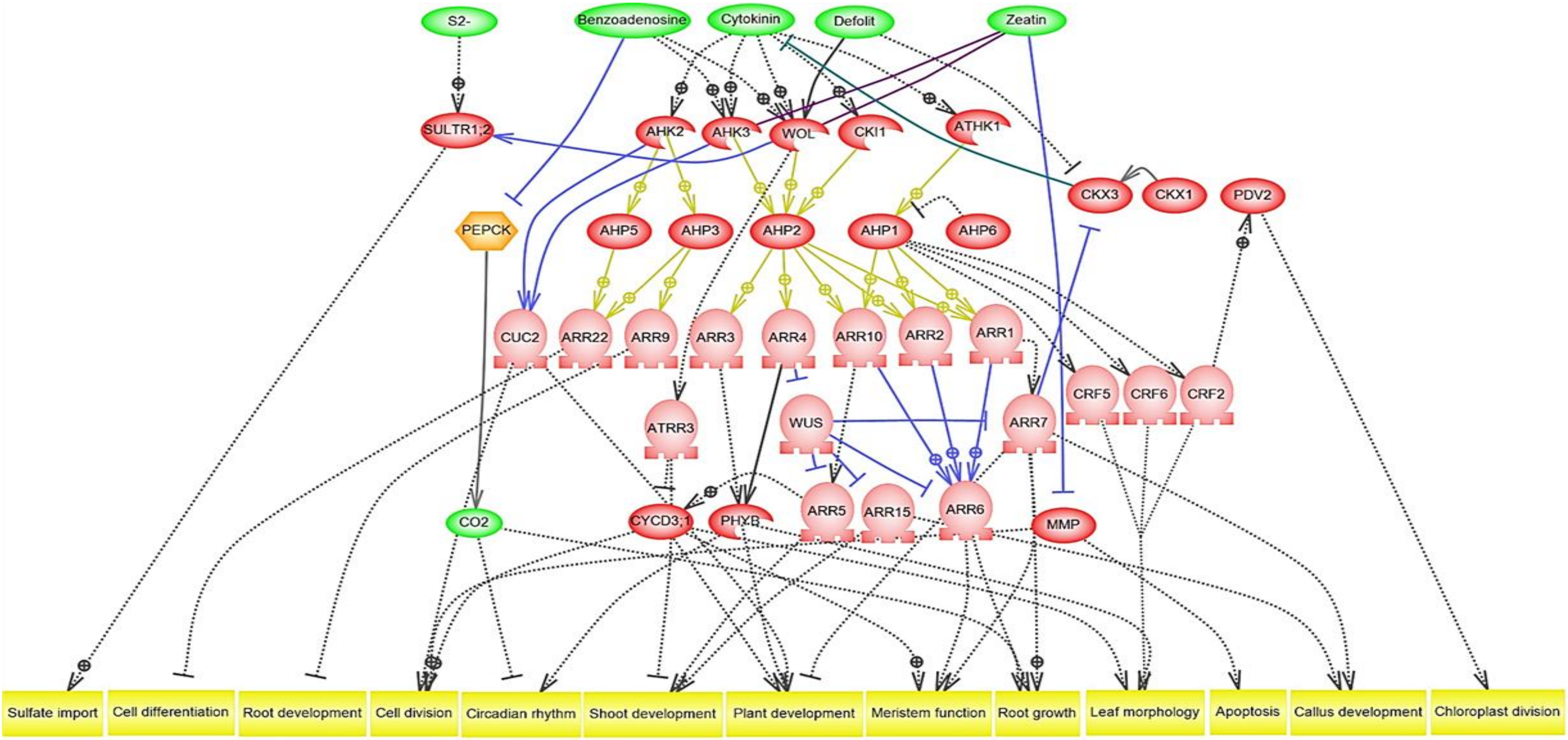

Figure S9. Cytokinin signaling pathway. This pathway shows that cytokinin responses are mediated by two-component systems and the signals are transmitted to the downstream cascade which leads to the biological changes in the plant.

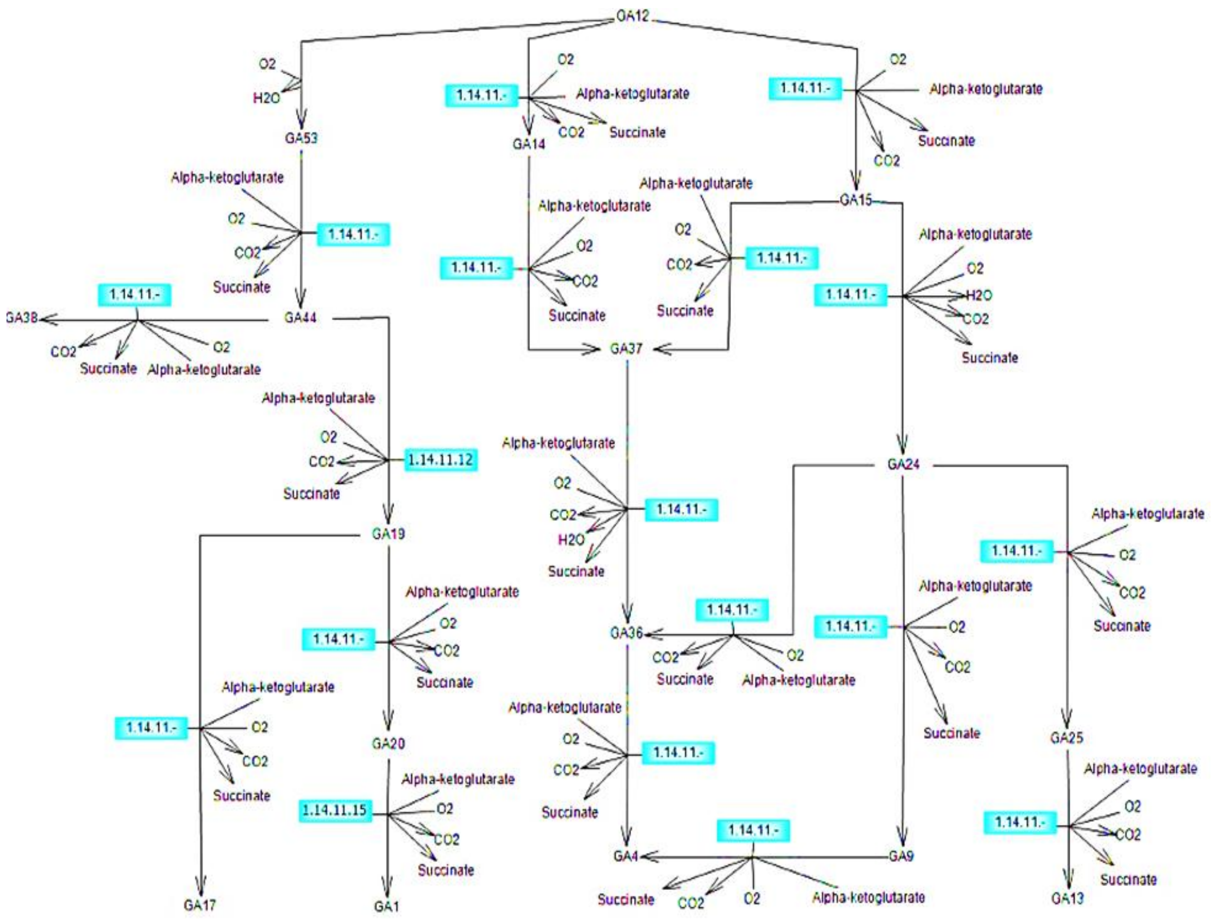

Figure S10. Gibberellin biosynthesis. Enzymes involved in the reaction are as listed: 1.14.11.12, gibberellin-44 dioxygenase; 1.14.11.-, oxidoreductase; 1.14.11.15, gibberellin 3β-dioxygenase.

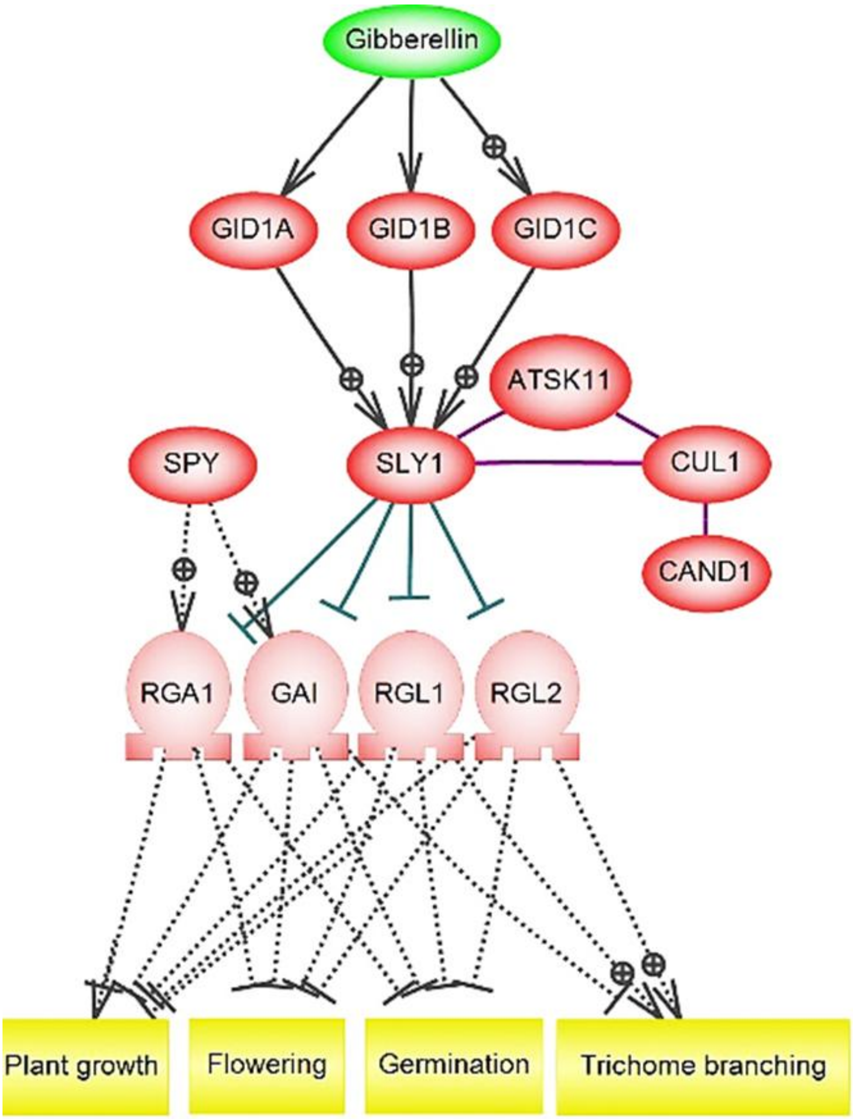

Figure S11. Gibberellin signaling pathway. Proteins involved in this pathway regulates plant growth, flowering, germination, and trichome branching. The central protein of this pathway is SLY1.

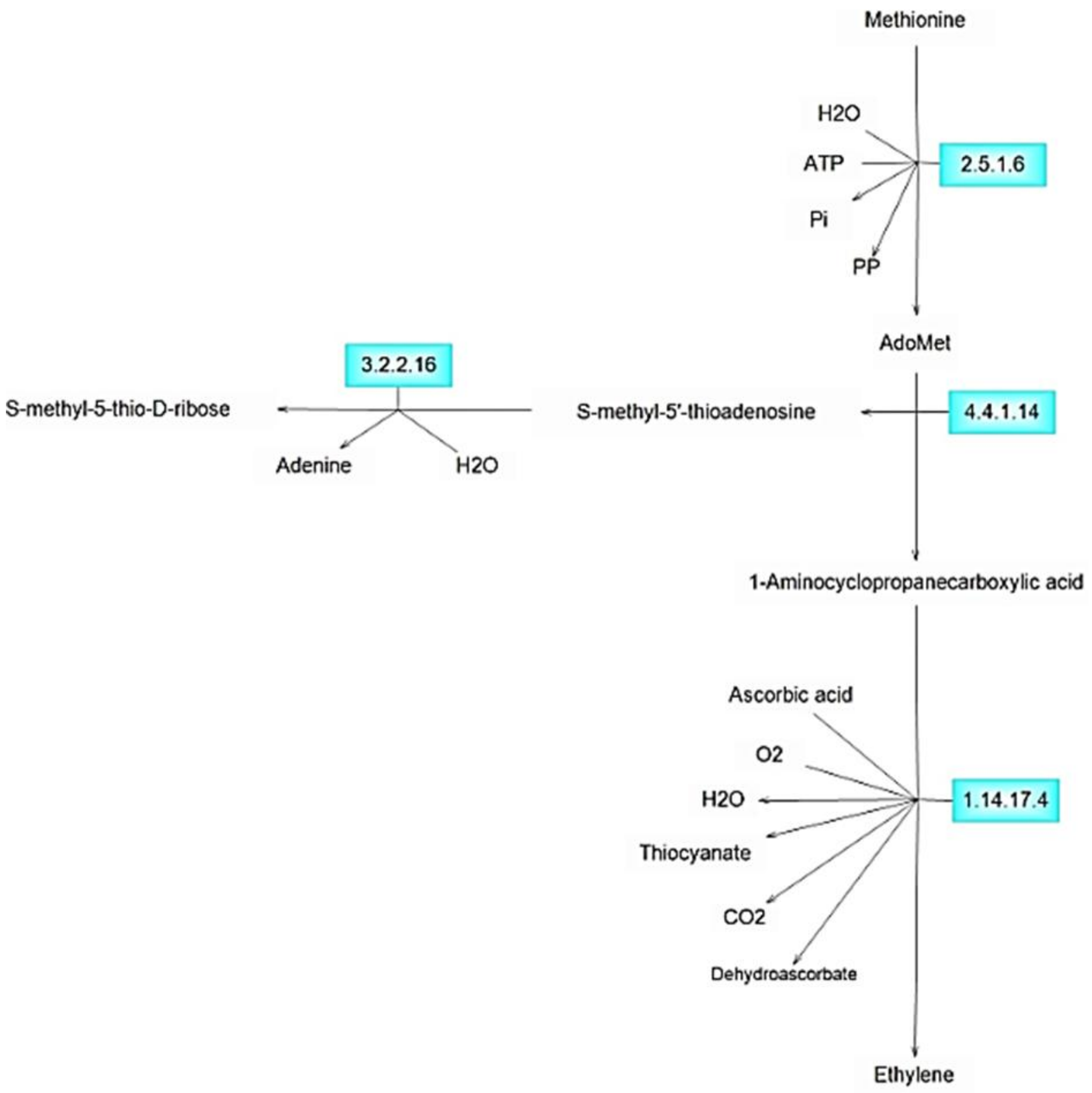

Figure S12. Ethylene biosynthesis. Enzymes involved in the reaction are as listed: 1.14.17.4, aminocyclopropanecarboxylate oxidase; 4.4.1.14, 1-aminocyclopropane-1-carboxylate synthase; 3.2.2.16, methylthioadenosine nucleosidase; 2.5.1.6, methionine adenosyltransferase.

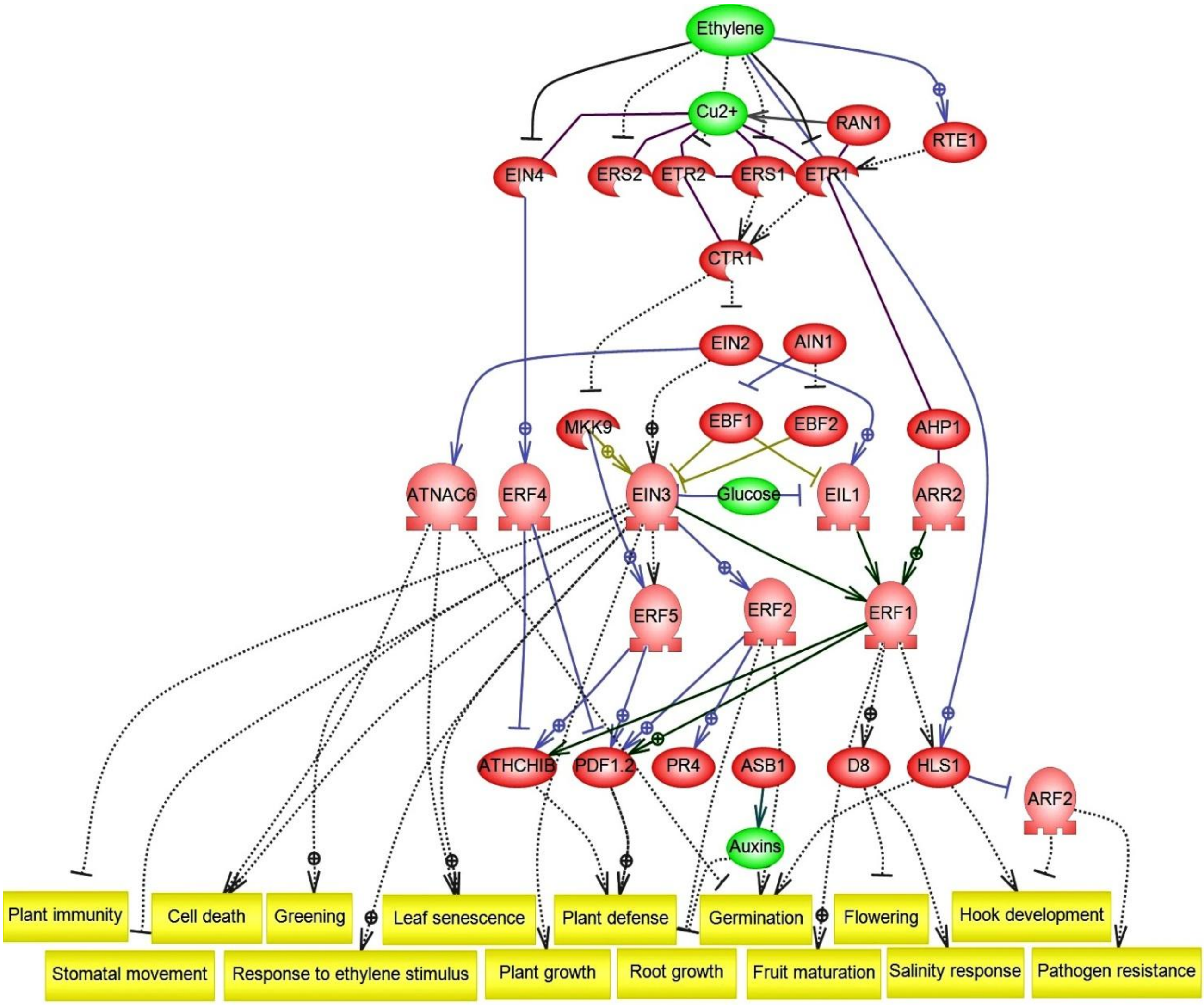

Figure S13. Ethylene signaling pathway. Ethylene regulates a number of proteins which are essential in controlling many aspects of plant growth and development. Some of the major regulators include ATNAC6, ERF4, EIN3, and ERF1.

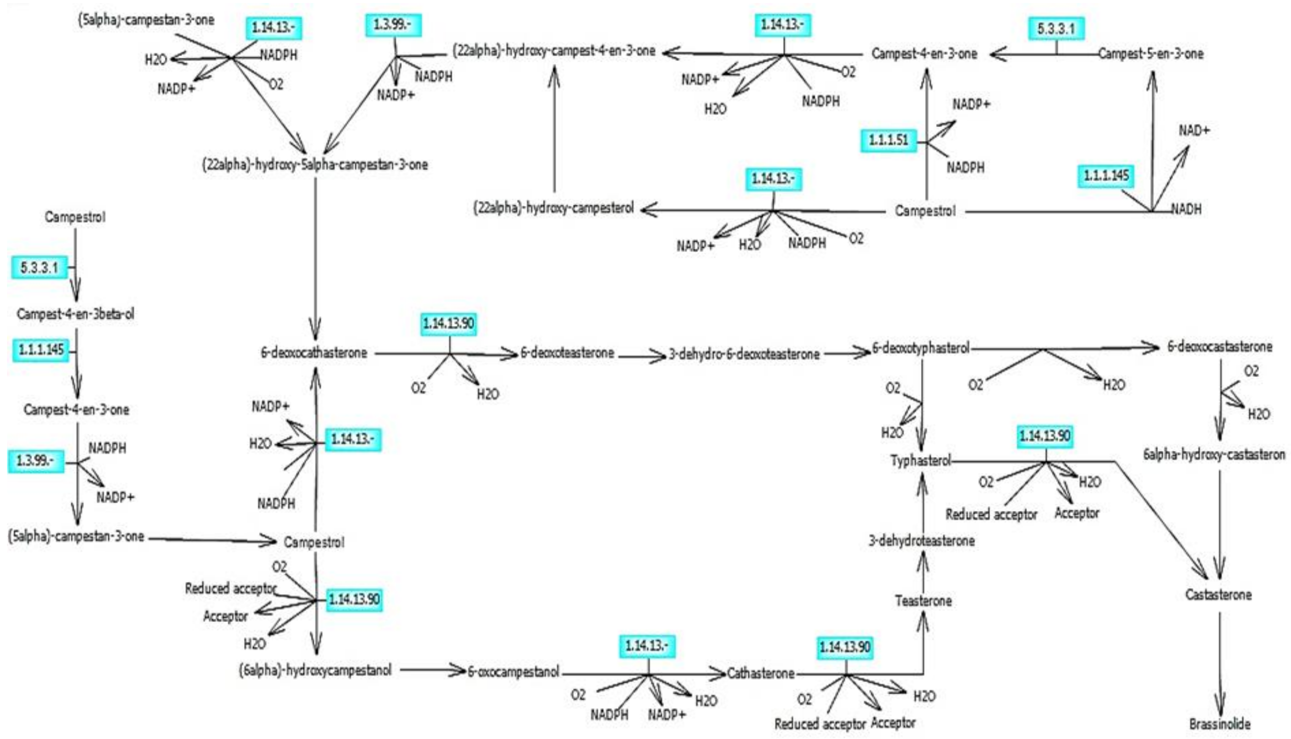

Figure S14. Brassinosteroid biosynthesis. Enzymes involved in the reaction are as listed: 1.14.13.-, β-carotene hydroxylase; 1.14.13.90, zeaxanthin epoxidase; 1.1.1.145, 3β-hydroxy-Δ5-steroid dehydrogenase; 1.1.1.51, 3(or 17)β-hydroxysteroid dehydrogenase; 5.3.3.1, steroid Δ-isomerase; 1.3.99.-, oxidoreductases.

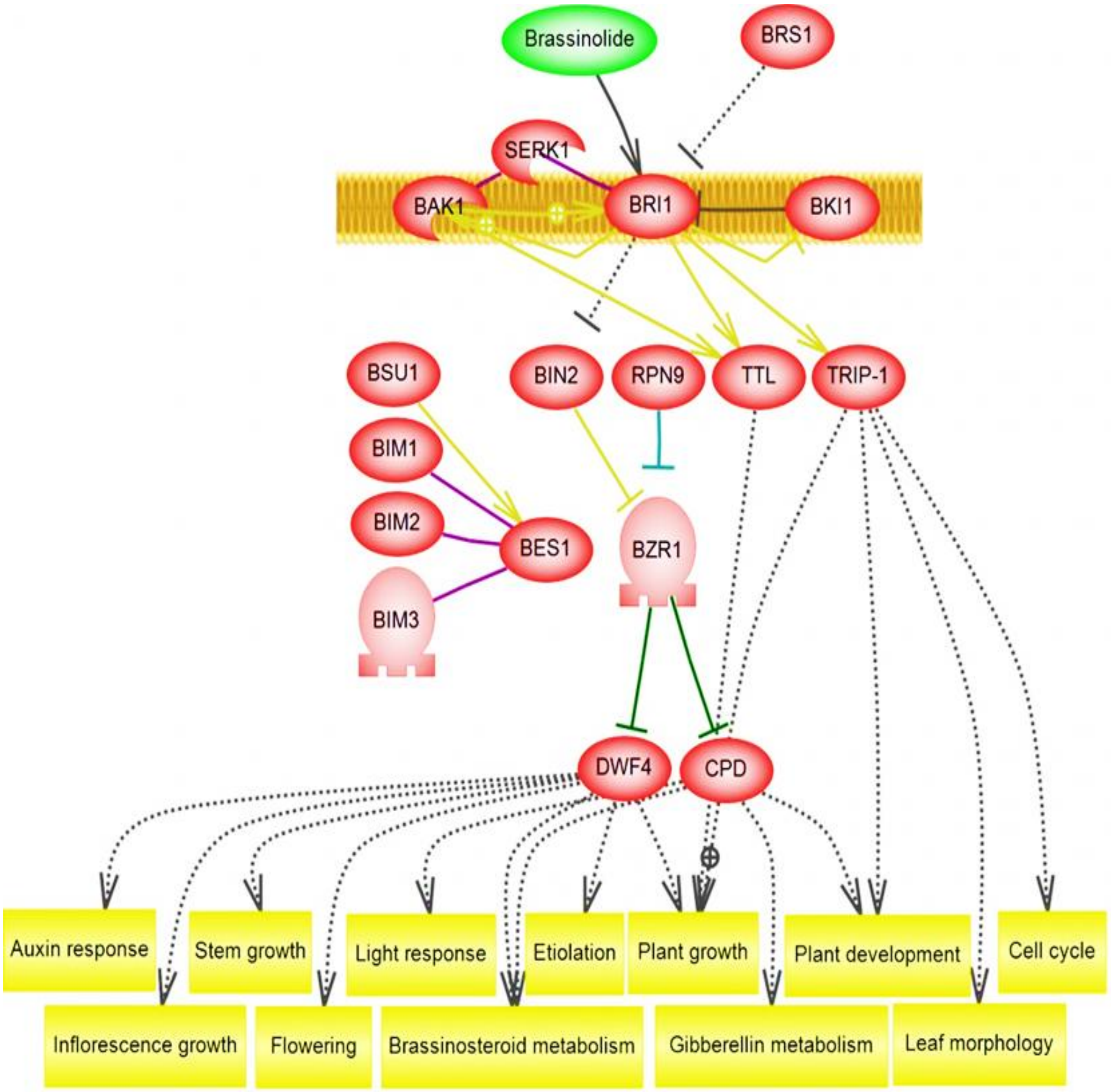

Figure S15. Brassinosteroid signaling pathway. The main protein in this pathway is BRI1. The signal received during the presence of brassinolide initiates intracellular signal transduction cascades which affect a number of biological changes in the plant.

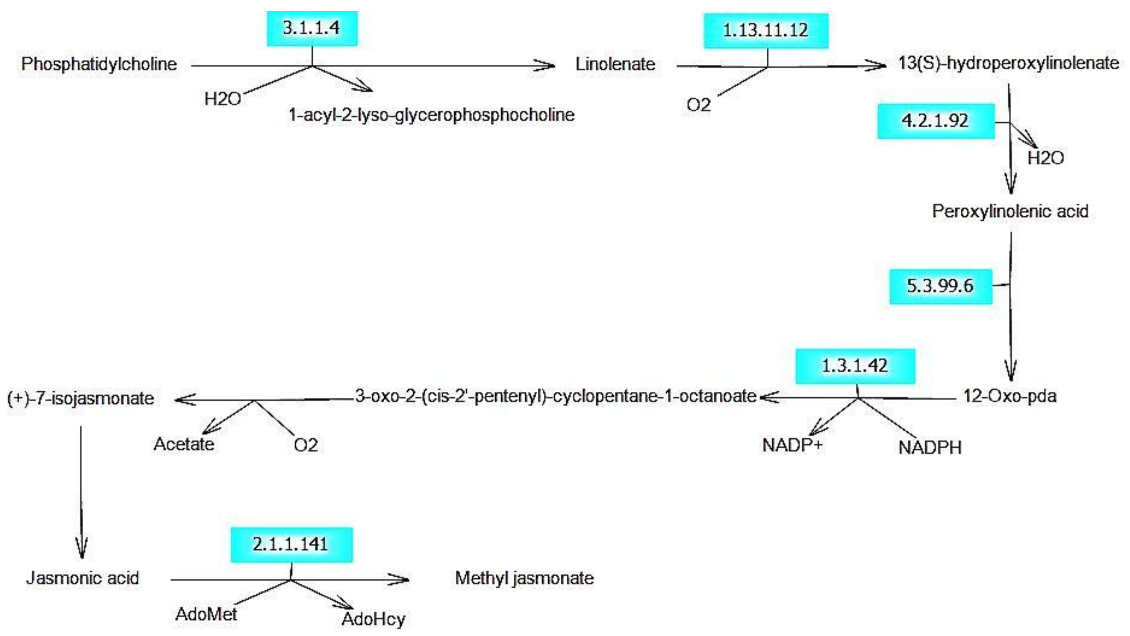

Figure S16. Jasmonic acid biosynthesis. Enzymes involved in the reaction are as listed: 1.13.11.12, linoleate 13S-lipoxygenase; 1.3.1.42, 12-oxophytodienoate reductase; 3.1.1.4, phospholipase A2; 5.3.99.6, allene-oxide cyclase; 2.1.1.141, jasmonate O-methyltransferase; 4.2.1.92, hydroperoxide dehydratase.

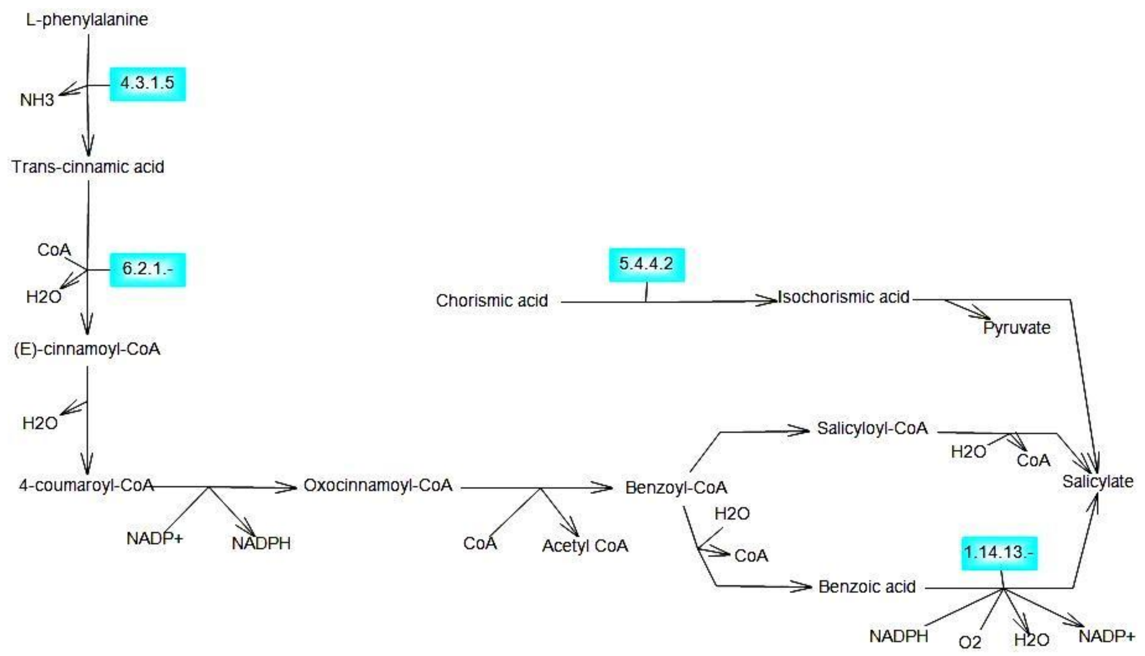

Figure S17. Salicylic acid biosynthesis. Enzymes involved in the reaction are as listed: 6.2.1.-, acid-thiol ligase; 1.14.13.-, β-carotene hydroxylase; 5.4.4.2, isochorismate synthase; 4.3.1.5, phenylalanine ammonia-lyase.

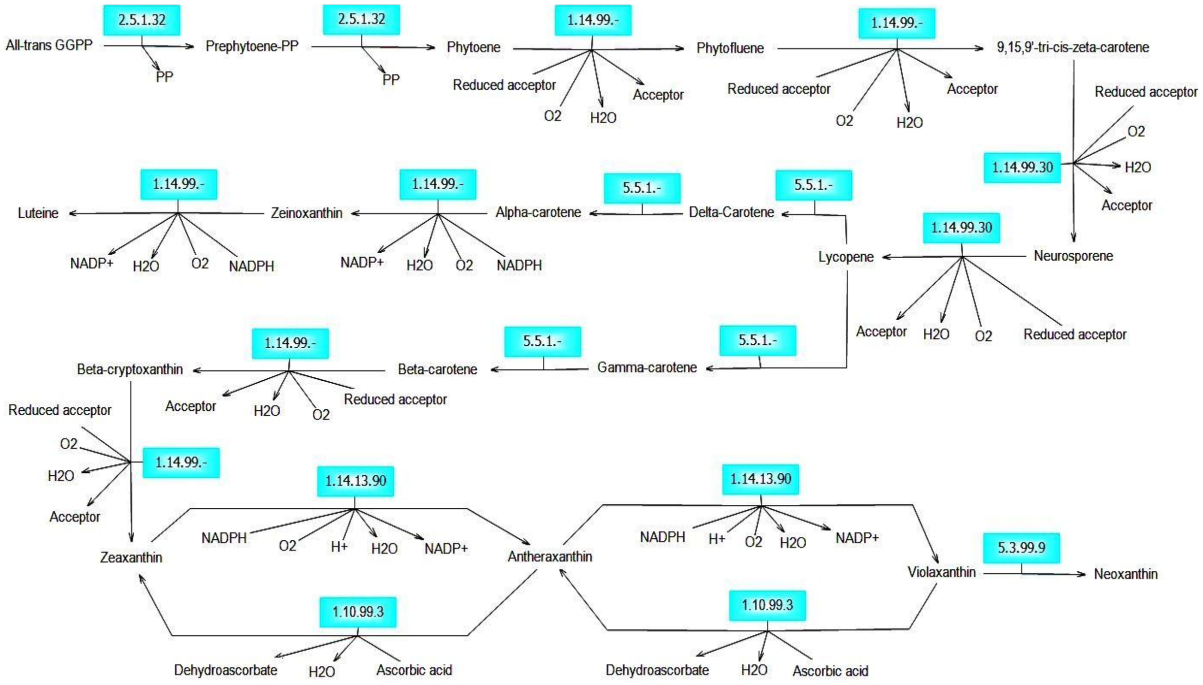

Figure S18. Carotenoid biosynthesis. Enzymes involved in the reaction are as listed: 2.5.1.32, phytoene synthase; 5.3.99.9, neoxanthin synthase; 5.5.1.-, intramolecular lyase; 1.14.13.90, zeaxanthin epoxidase; 1.14.99.-, phytoene dehydrogenase; 1.14.99.30, carotene 7,8-desaturase; 1.10.99.3, violaxanthin de-epoxidase.
